# Supplementary figures and images for: Autophagosomes anchor an AKAP11-dependent regulatory checkpoint that shapes neuronal PKA signaling (part 1 of 2)
Source: EMBO J. 2025 Apr 22;44(11):3150–79. doi: 10.1038/s44318-025-00436-x (PMC12130464; doi:10.1038/s44318-025-00436-x)

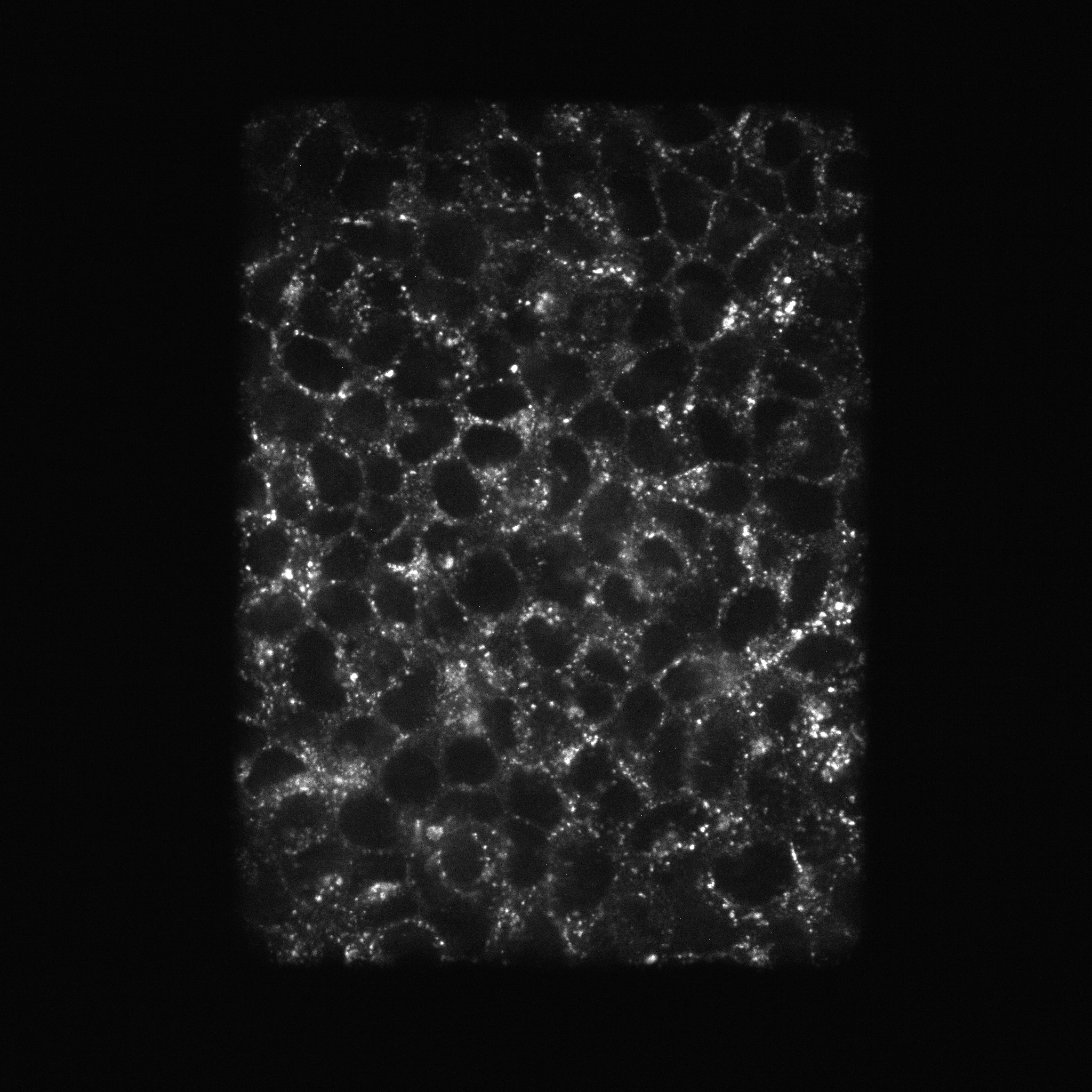

Supplement: Supplementary file 6 — Source data Fig. 2 [file 44318_2025_436_MOESM6_ESM.zip › SD_Figure 2/2C/293T-sgATG7_F-PKA-RI_Torin-BafA_6_20200401_43142 PM/293T-sgATG7_F-PKA-RI_Torin-BafA_6_w0001.tif]

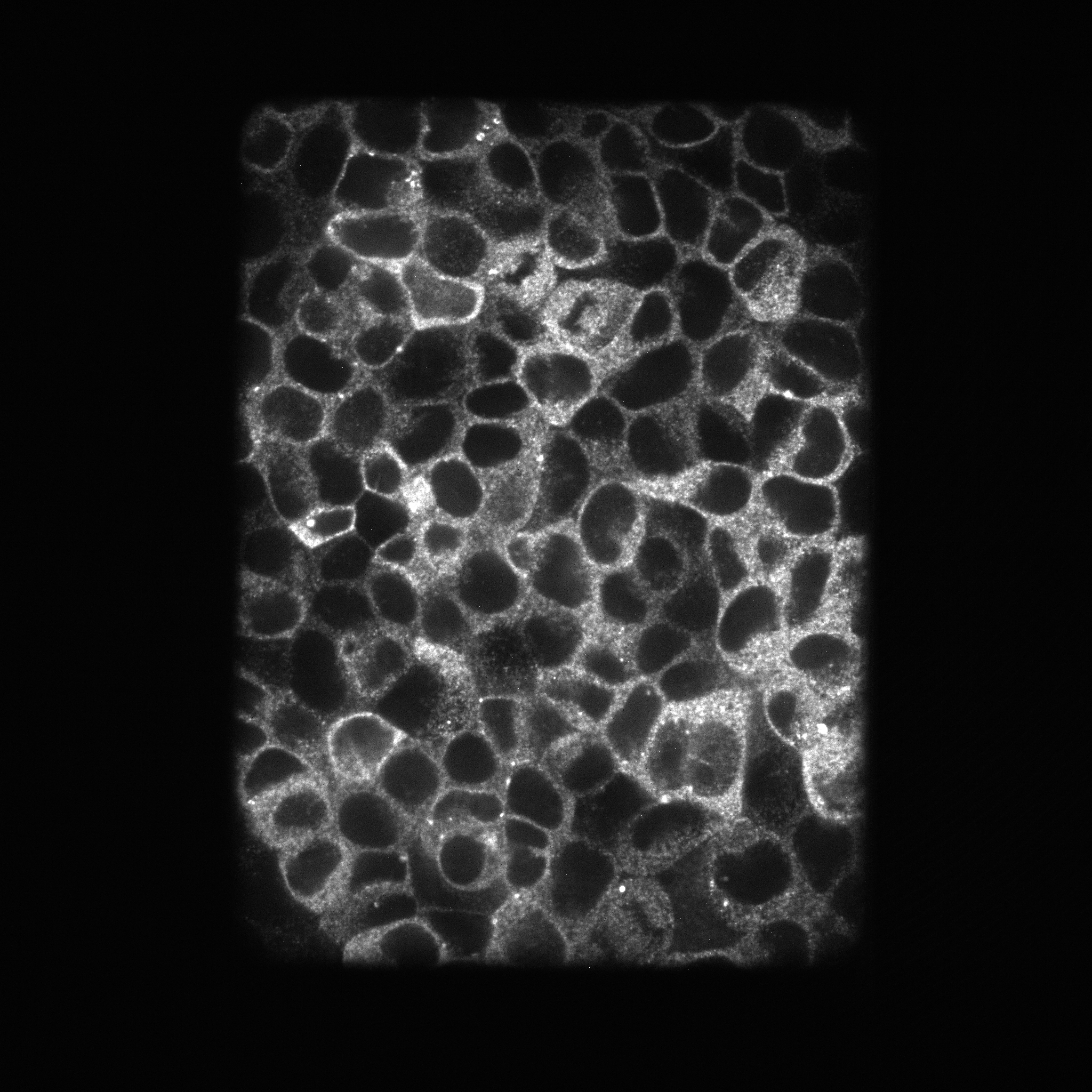

Supplement: Supplementary file 6 — Source data Fig. 2 [file 44318_2025_436_MOESM6_ESM.zip › SD_Figure 2/2C/293T-sgATG7_F-PKA-RI_Torin-BafA_6_20200401_43142 PM/293T-sgATG7_F-PKA-RI_Torin-BafA_6_w0000.tif]

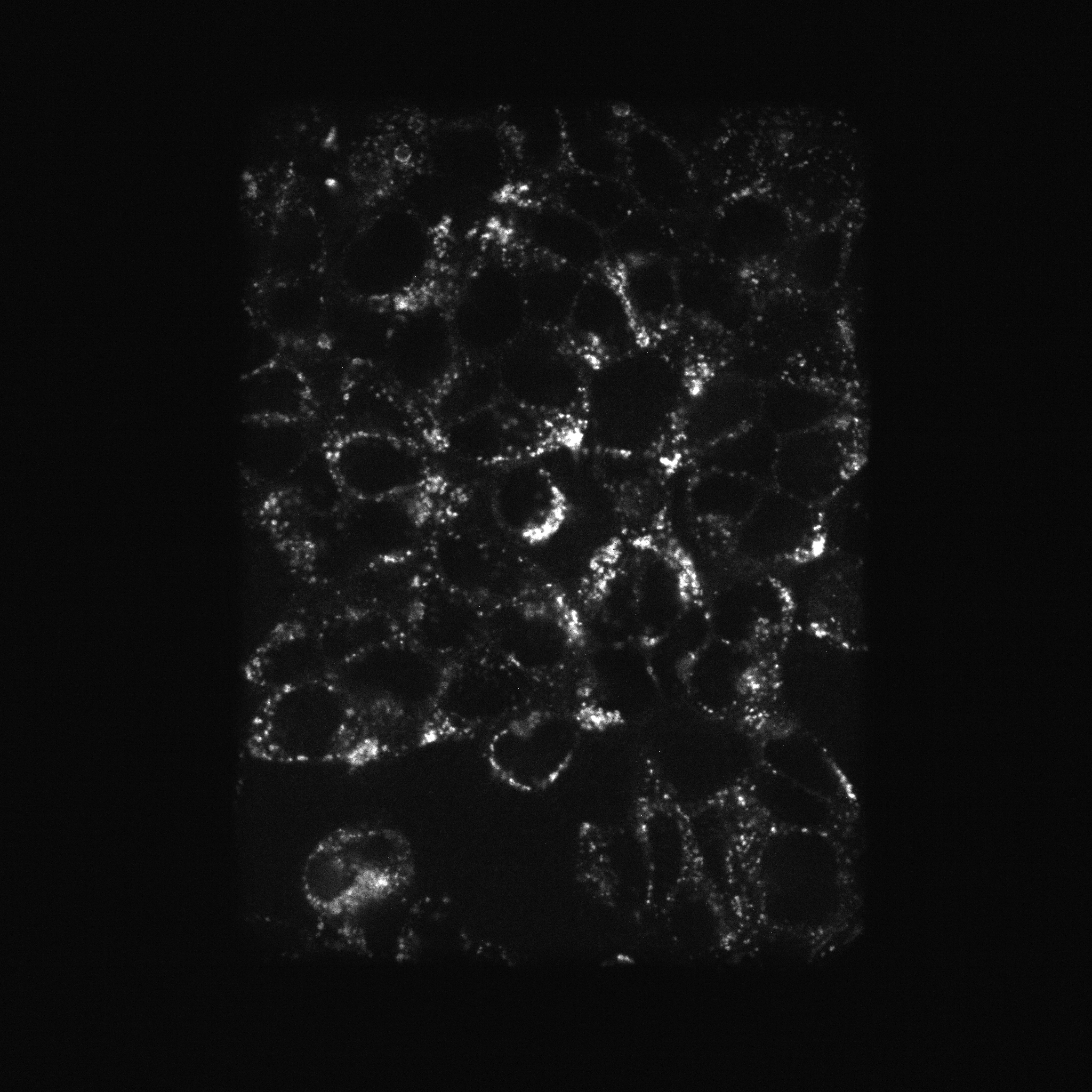

Supplement: Supplementary file 6 — Source data Fig. 2 [file 44318_2025_436_MOESM6_ESM.zip › SD_Figure 2/2C/293T-WT_F-PKA-RI_Torin-BafA_2_20200401_41750 PM/293T-WT_F-PKA-RI_Torin-BafA_2_w0001.tif]

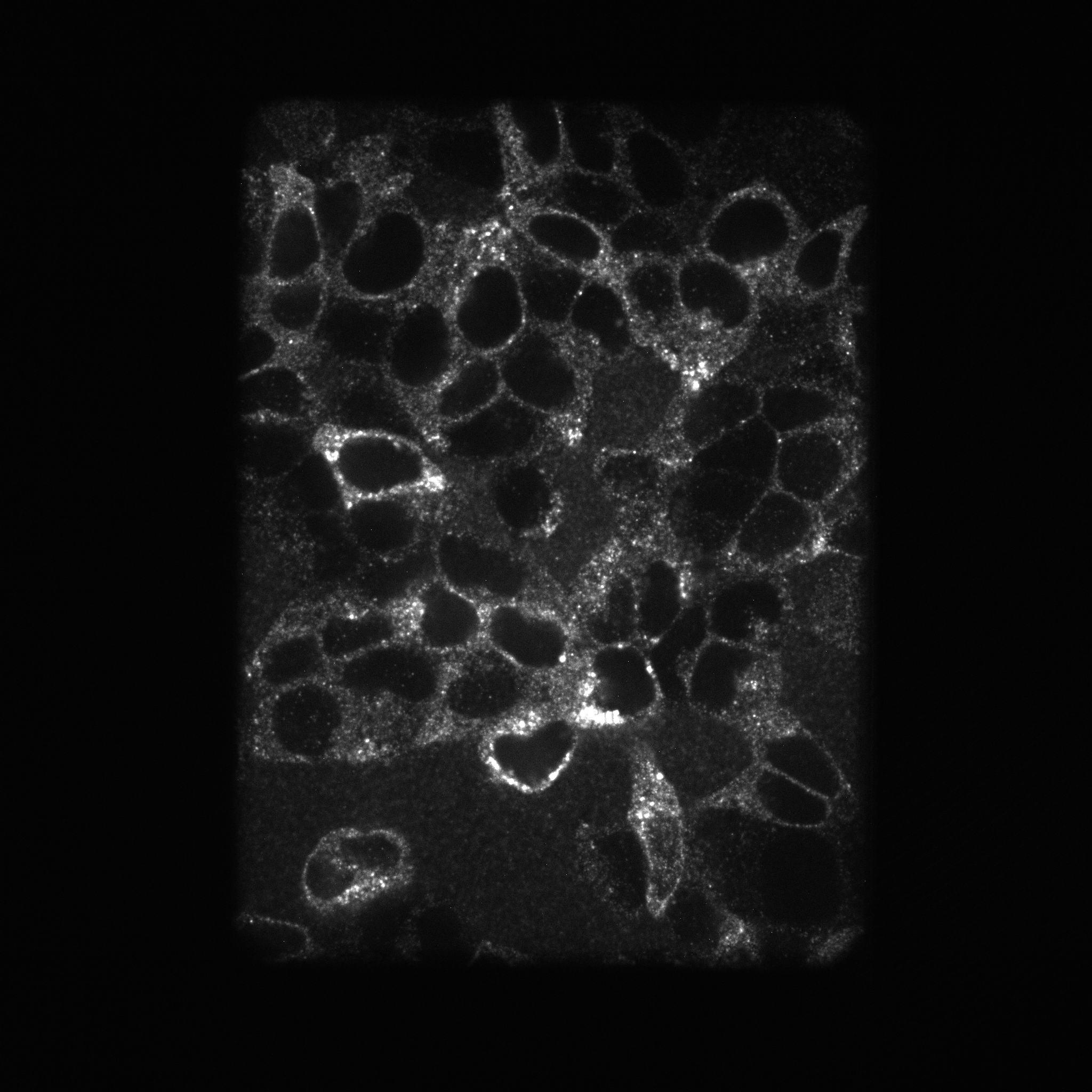

Supplement: Supplementary file 6 — Source data Fig. 2 [file 44318_2025_436_MOESM6_ESM.zip › SD_Figure 2/2C/293T-WT_F-PKA-RI_Torin-BafA_2_20200401_41750 PM/293T-WT_F-PKA-RI_Torin-BafA_2_w0000.tif]

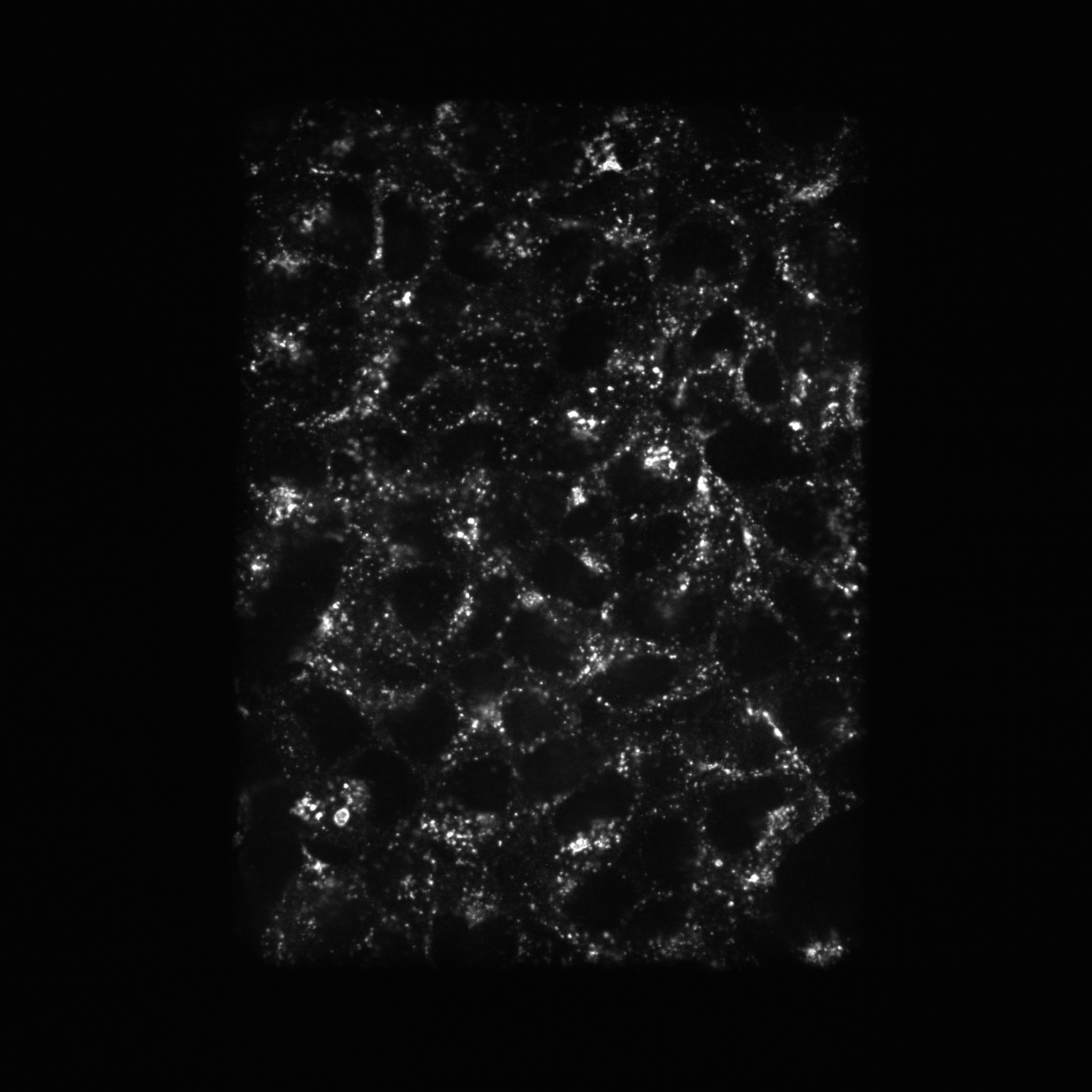

Supplement: Supplementary file 6 — Source data Fig. 2 [file 44318_2025_436_MOESM6_ESM.zip › SD_Figure 2/2C/293T-WT_F-PKA-RI_CM_6_20200401_43629 PM/293T-WT_F-PKA-RI_CM_6_w0001.tif]

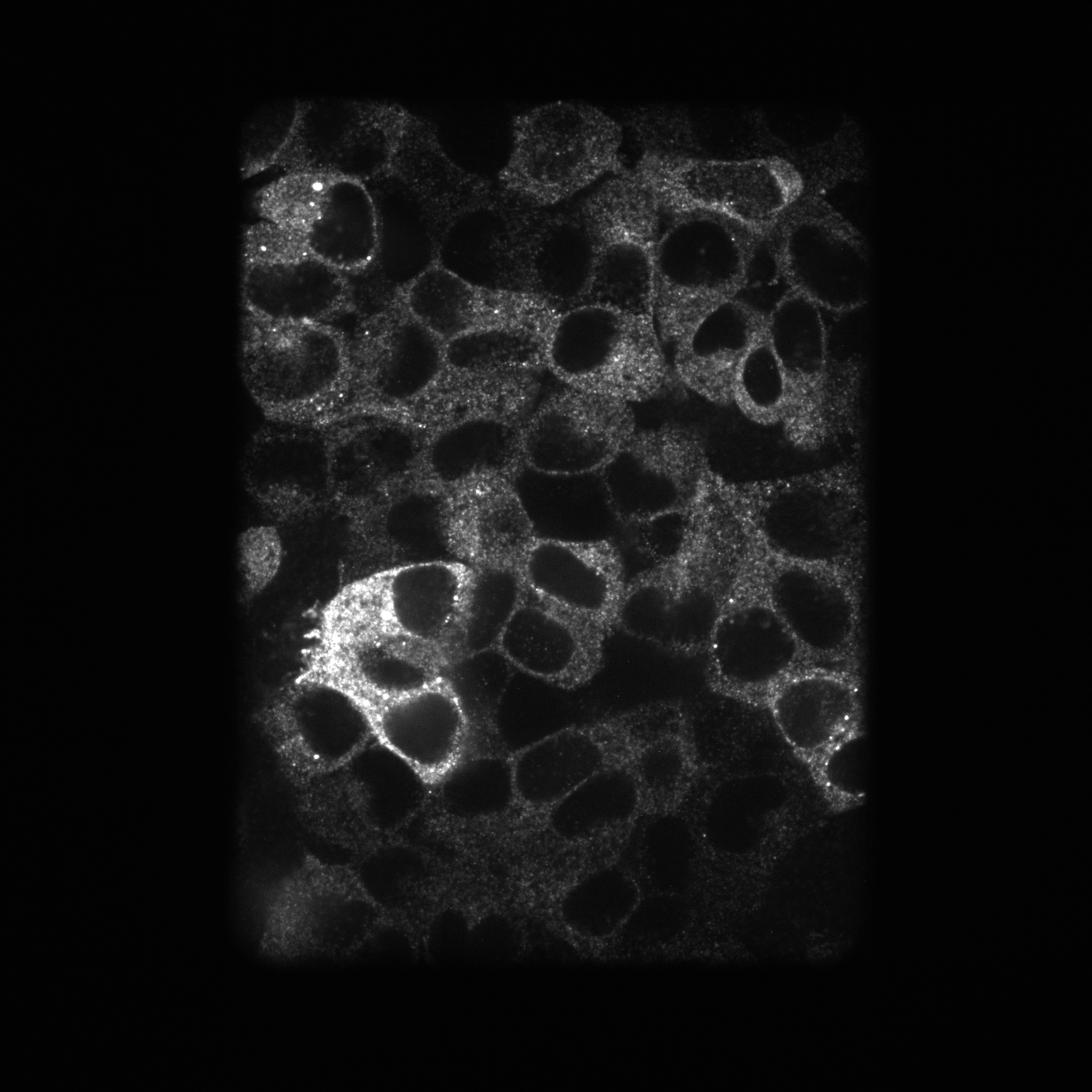

Supplement: Supplementary file 6 — Source data Fig. 2 [file 44318_2025_436_MOESM6_ESM.zip › SD_Figure 2/2C/293T-WT_F-PKA-RI_CM_6_20200401_43629 PM/293T-WT_F-PKA-RI_CM_6_w0000.tif]

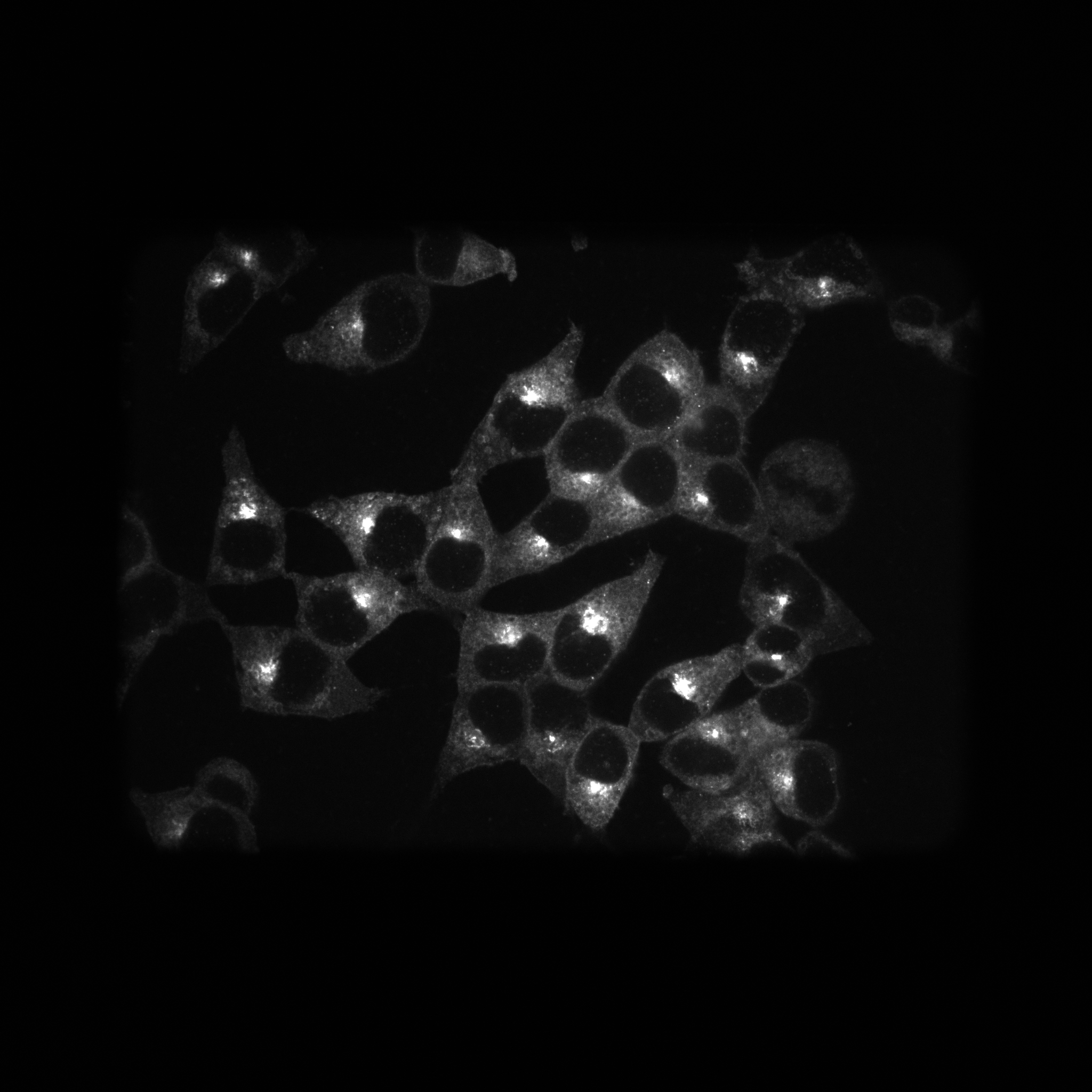

Supplement: Supplementary file 6 — Source data Fig. 2 [file 44318_2025_436_MOESM6_ESM.zip › SD_Figure 2/2E/Fig2E/20240606_shFIP200_BAF/20240606_mNPKACALAMP2_shFIP200_BAF_03.tif]

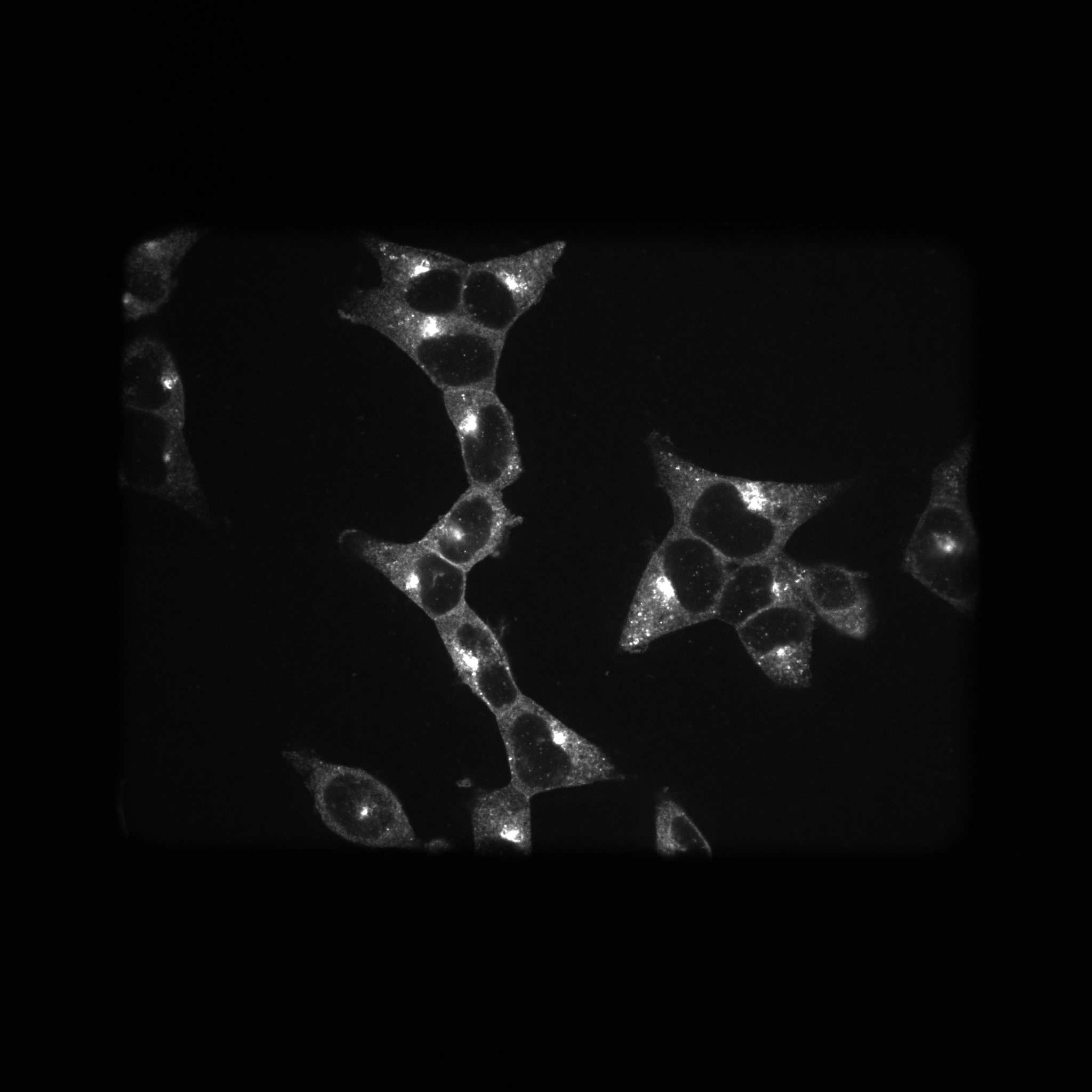

Supplement: Supplementary file 6 — Source data Fig. 2 [file 44318_2025_436_MOESM6_ESM.zip › SD_Figure 2/2E/Fig2E/20240606_shLUC_BAF/20240606_mNPKACALAMP2_shLUC_BAF_010.tif]

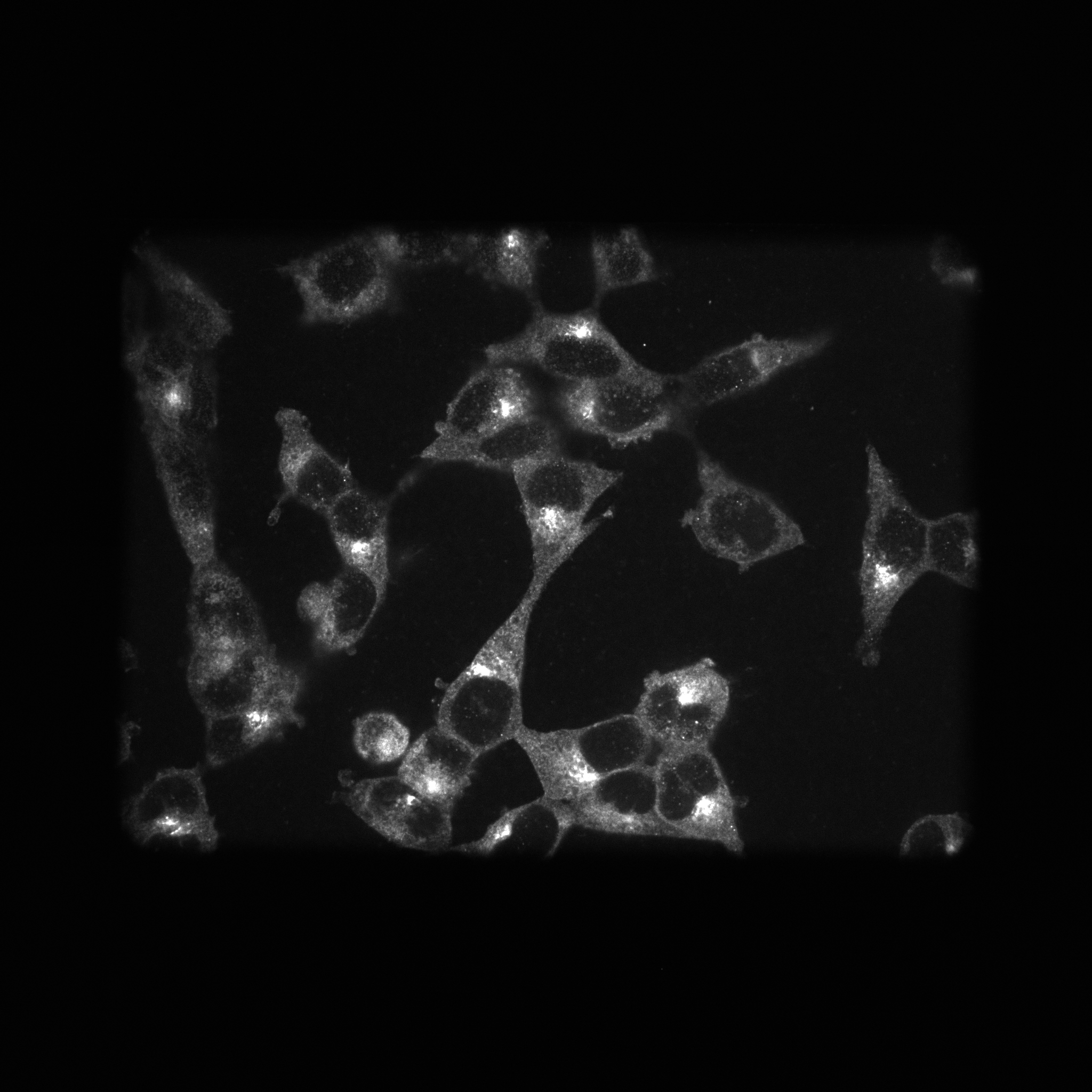

Supplement: Supplementary file 6 — Source data Fig. 2 [file 44318_2025_436_MOESM6_ESM.zip › SD_Figure 2/2E/Fig2E/20240606_shLUC_DMSO/20240606_mNPKACALAMP2_shLUC_DMSO_01.tif]

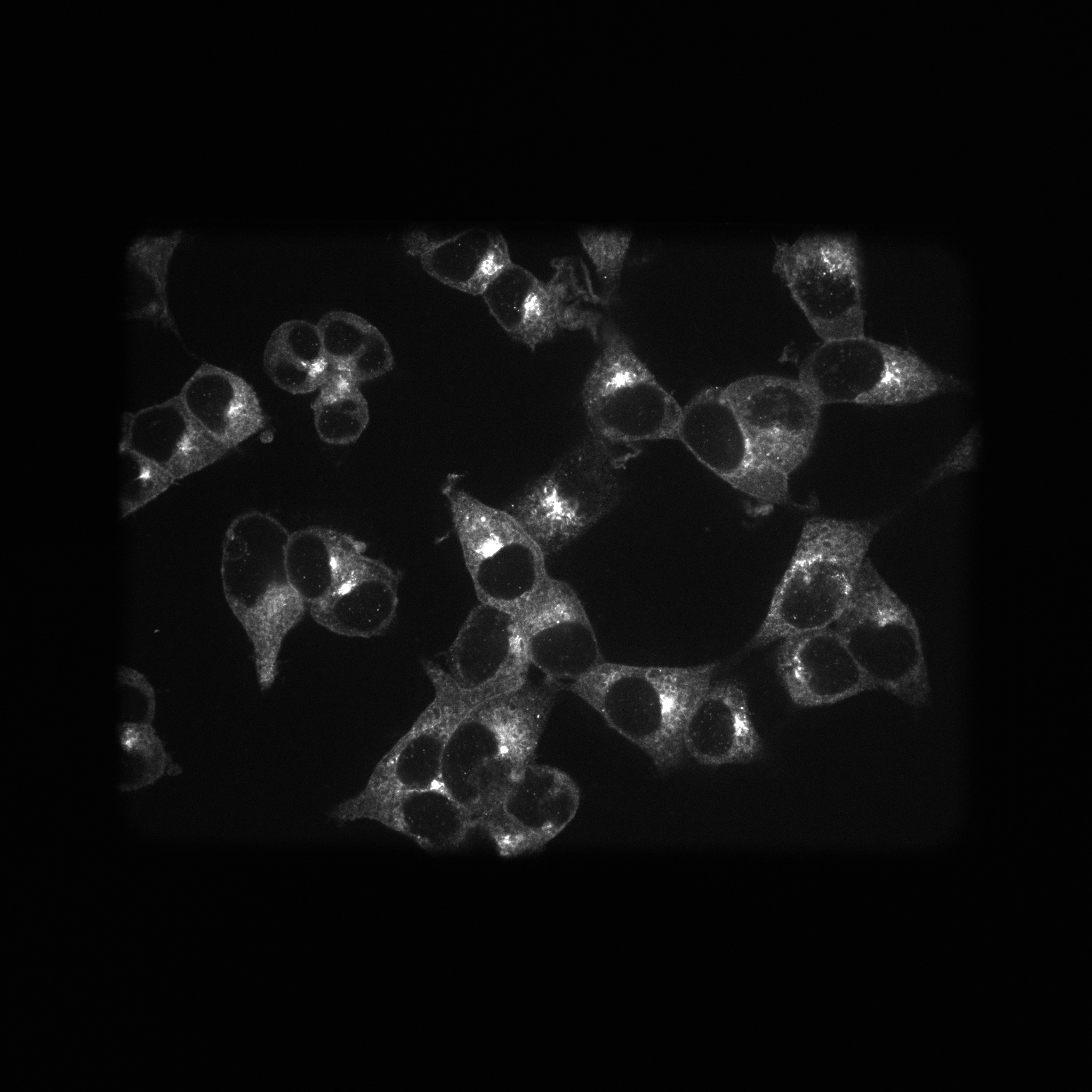

Supplement: Supplementary file 6 — Source data Fig. 2 [file 44318_2025_436_MOESM6_ESM.zip › SD_Figure 2/2E/Fig2E/20240606_shFIP200_DMSO/20240606_mNPKACALAMP2_shFIP200_DMSO_10.tif]

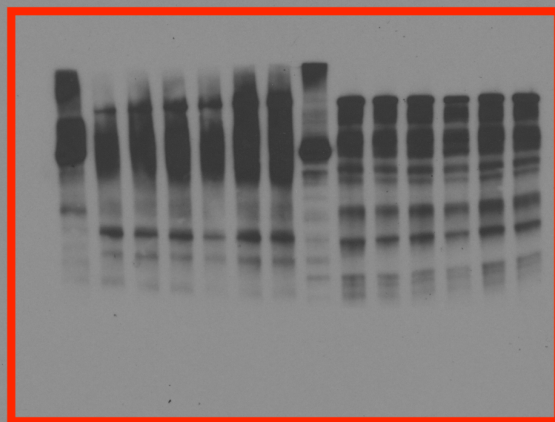

anti-HA

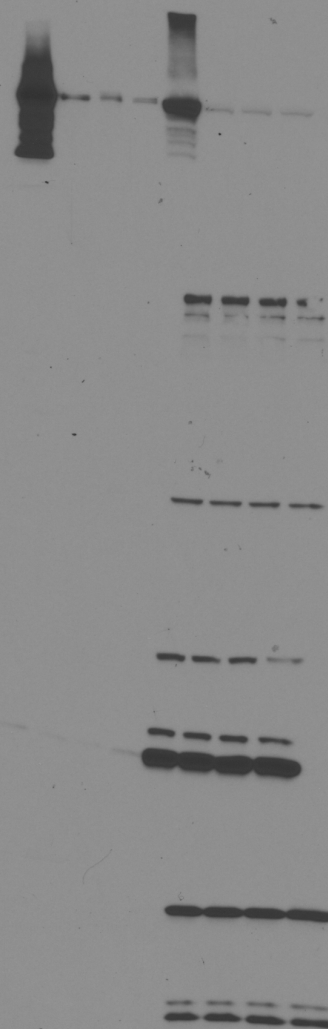

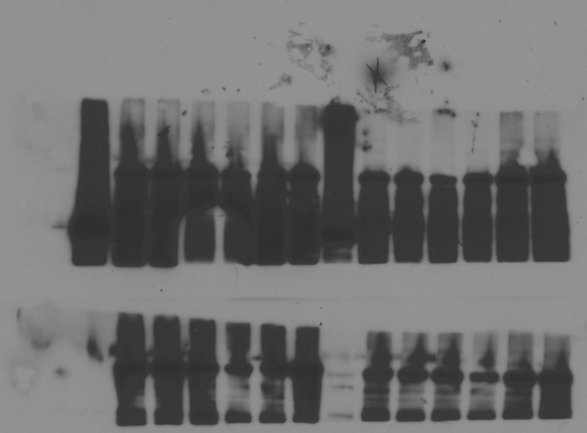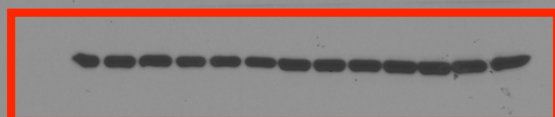

**PKA R1a**

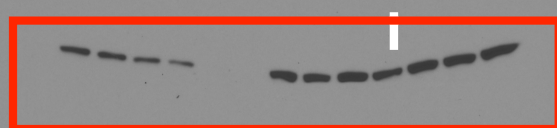

**PKA Ca**

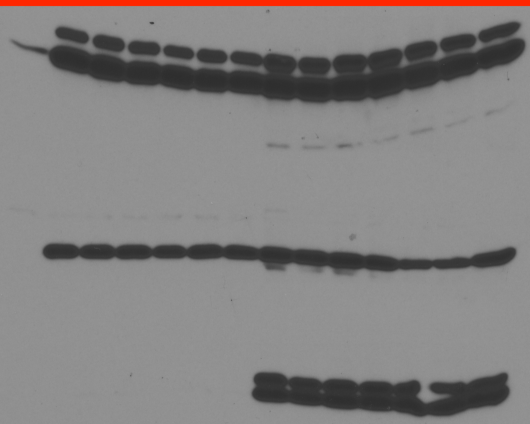

Supplement: Supplementary file 7 — Source data Fig. 3 [file 44318_2025_436_MOESM7_ESM.zip › Figure 3/3B/3B.pdf]

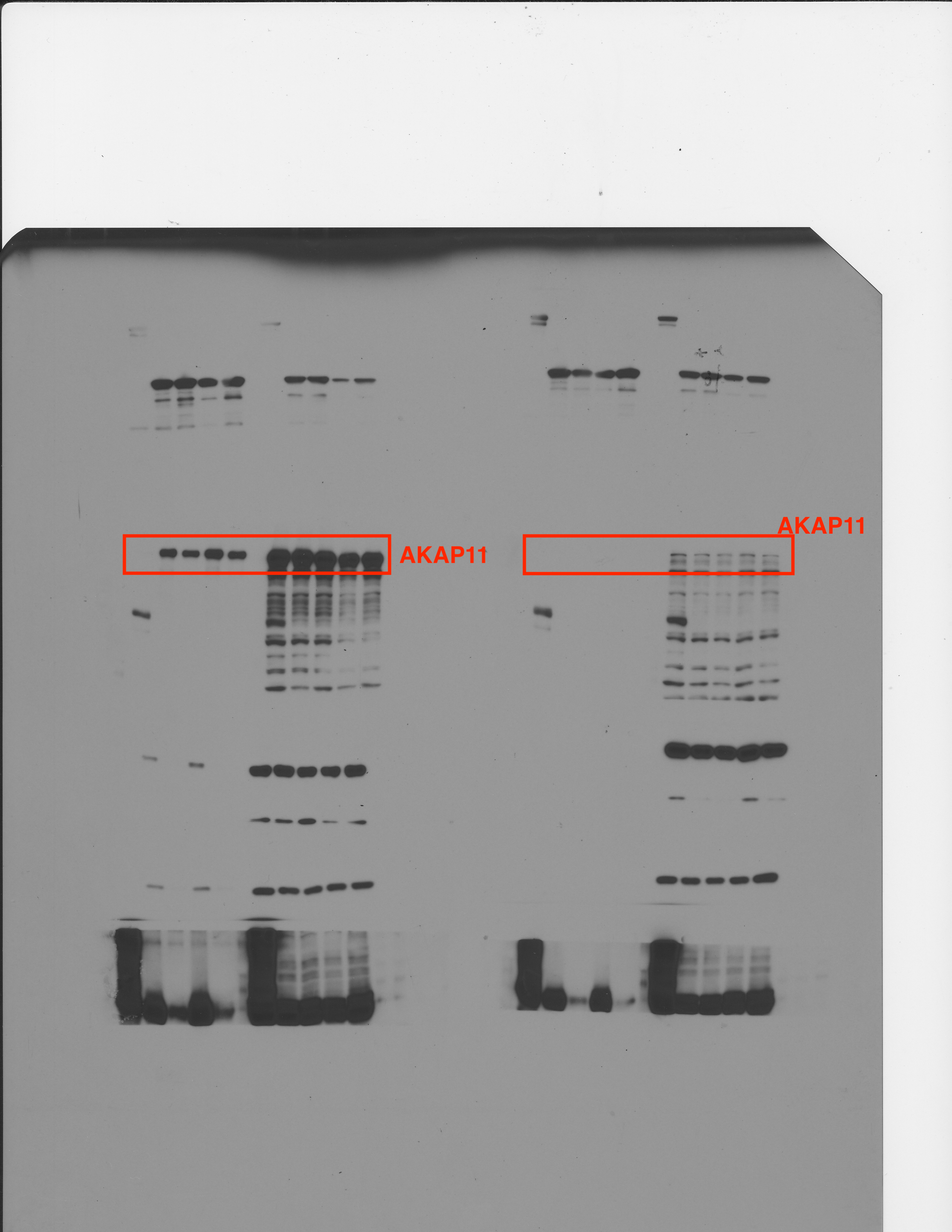

Supplement: Supplementary file 7 — Source data Fig. 3 [file 44318_2025_436_MOESM7_ESM.zip › Figure 3/3C/2021.07.28 AKAP11_WT GFP IP_0005.tif]

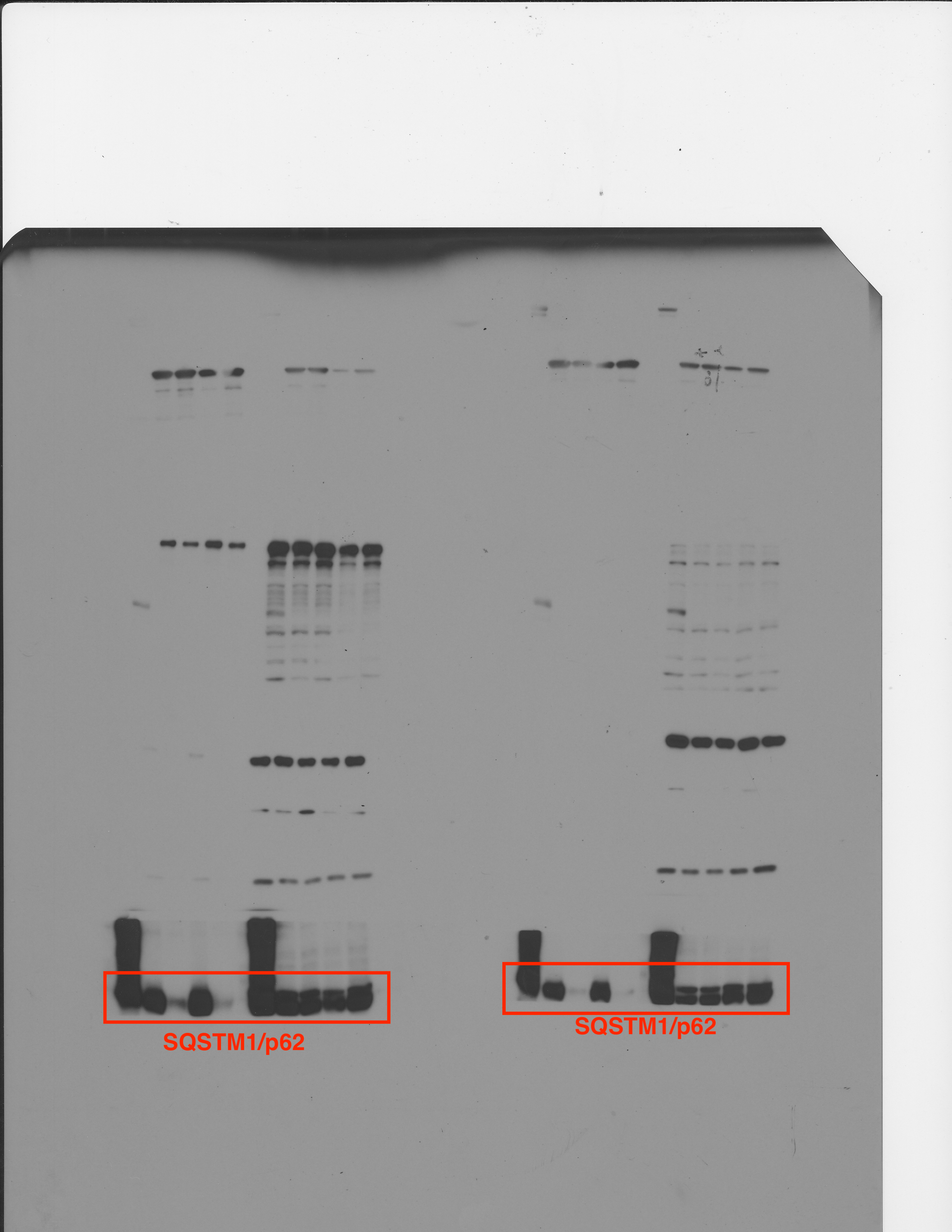

Supplement: Supplementary file 7 — Source data Fig. 3 [file 44318_2025_436_MOESM7_ESM.zip › Figure 3/3C/2021.07.28 AKAP11_WT GFP IP_0003.tif]

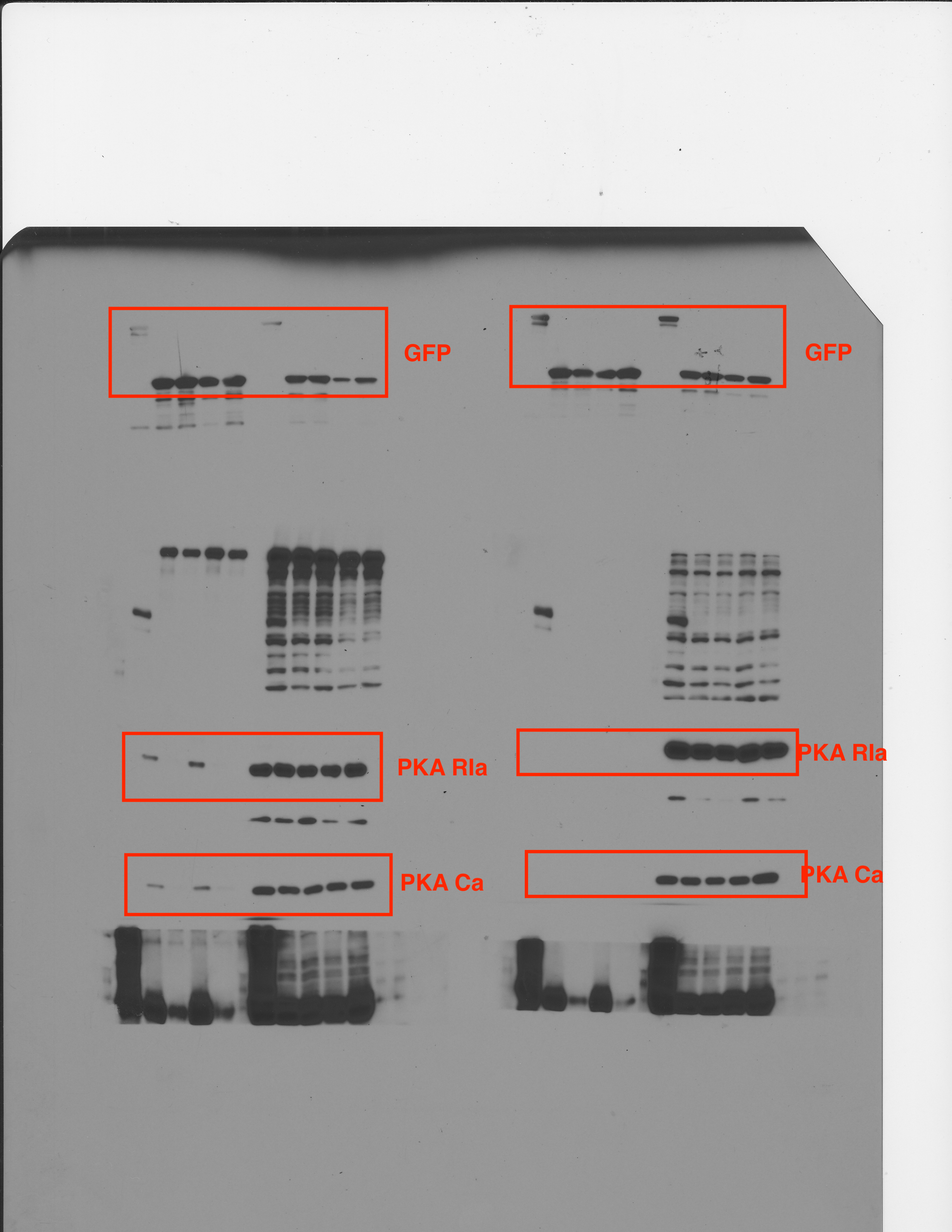

Supplement: Supplementary file 7 — Source data Fig. 3 [file 44318_2025_436_MOESM7_ESM.zip › Figure 3/3C/2021.07.28 AKAP11_WT GFP IP_0002.tif]

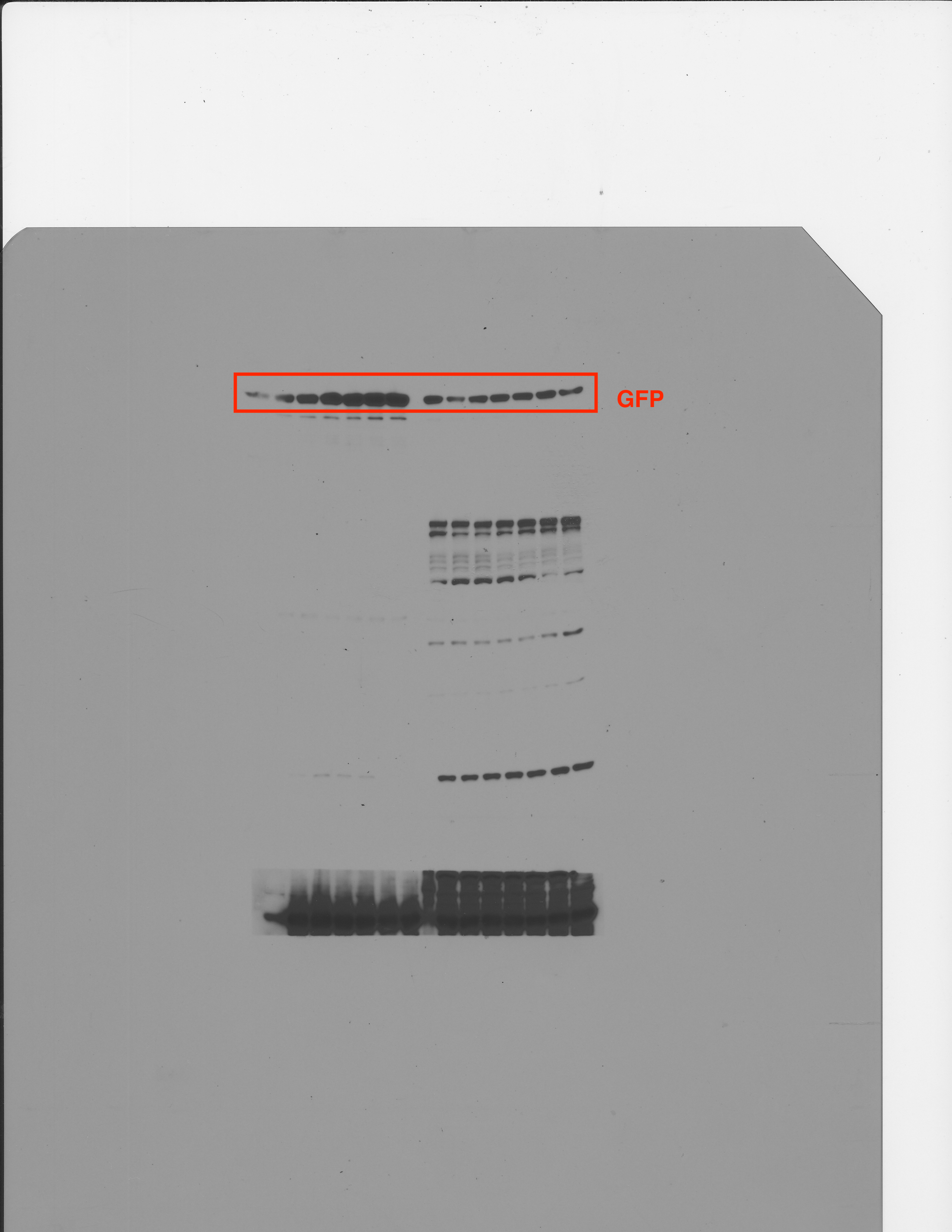

Supplement: Supplementary file 7 — Source data Fig. 3 [file 44318_2025_436_MOESM7_ESM.zip › Figure 3/3D/2022.07.18 GFP-LC3 IP_FSK DOSE _0004.tif]

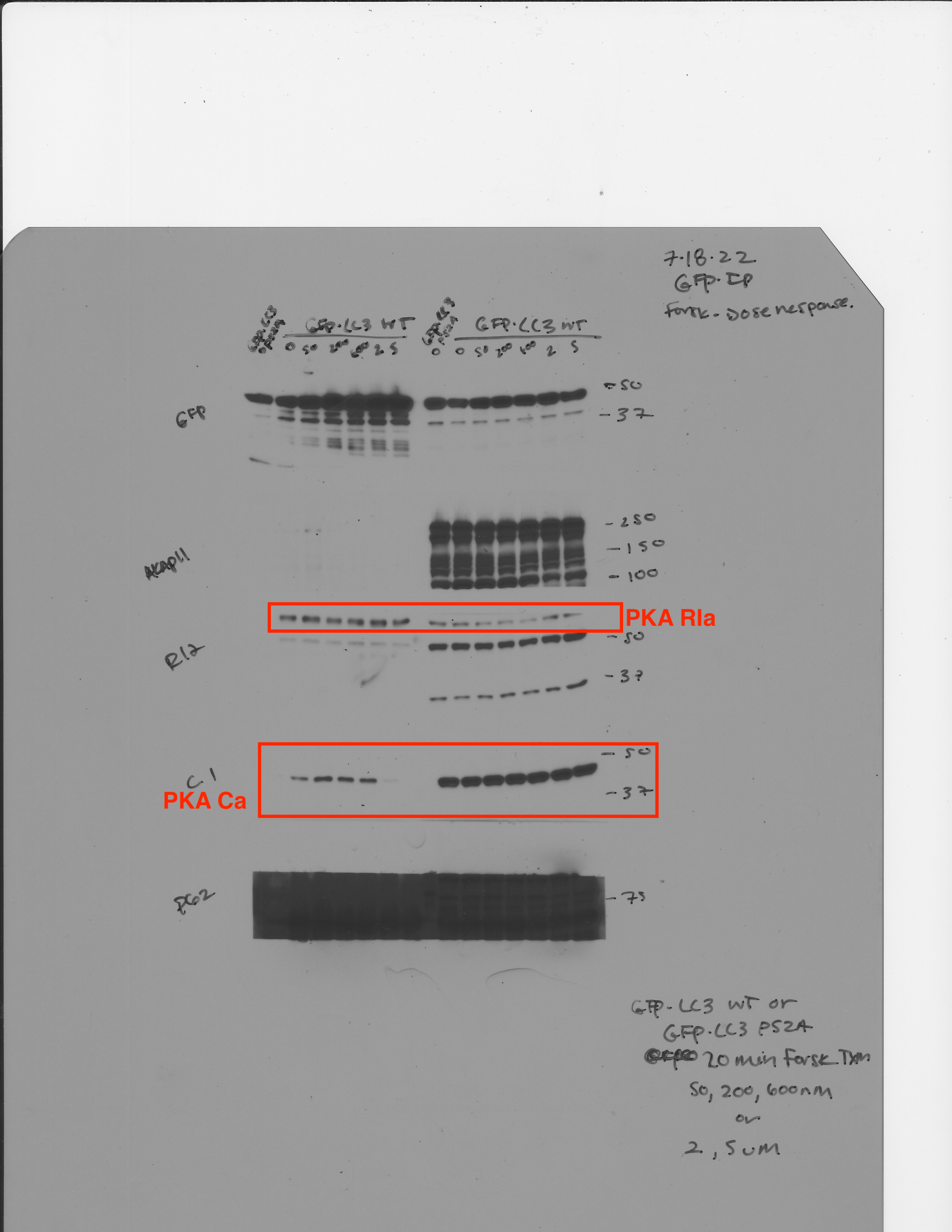

Supplement: Supplementary file 7 — Source data Fig. 3 [file 44318_2025_436_MOESM7_ESM.zip › Figure 3/3D/2022.07.18 GFP-LC3 IP_FSK DOSE _0001.tif]

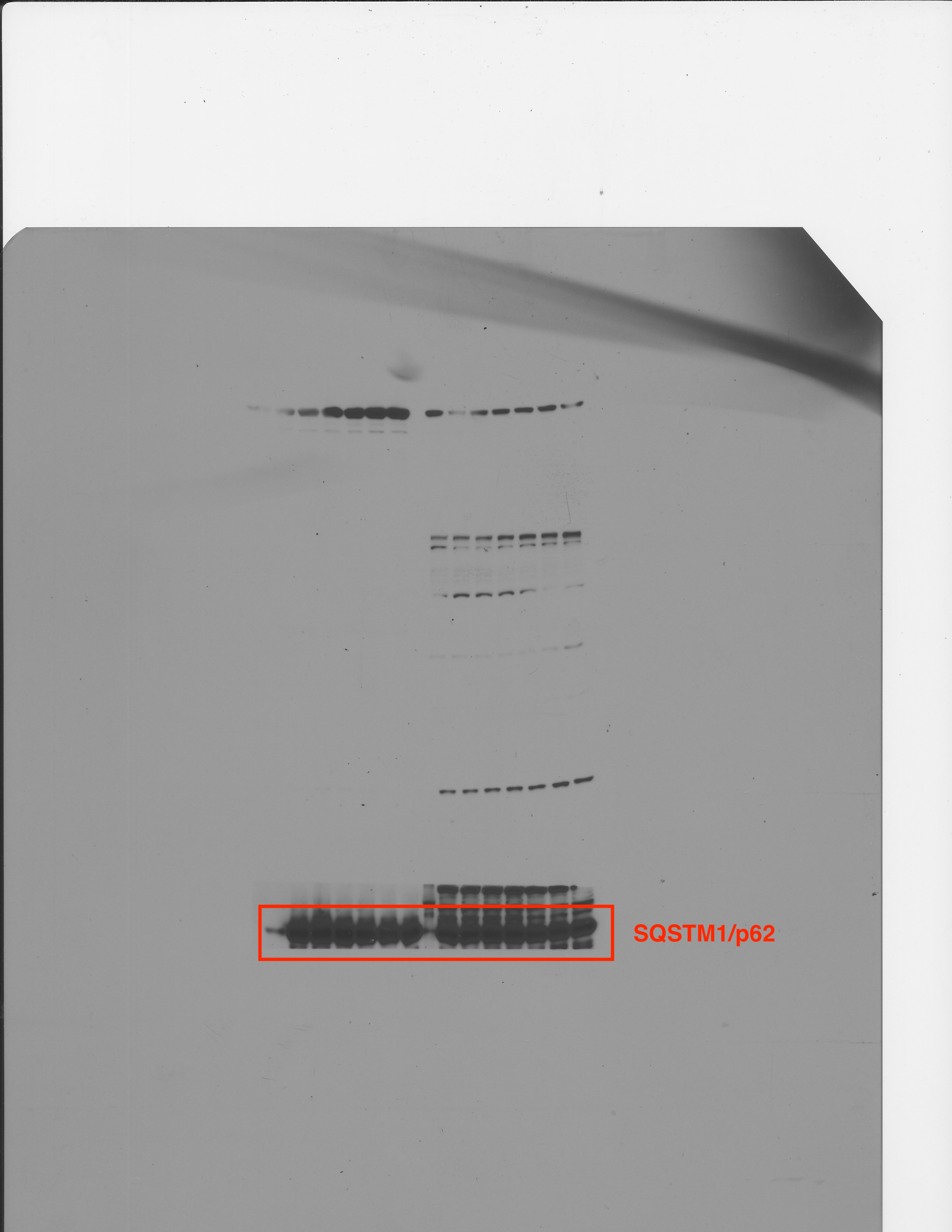

Supplement: Supplementary file 7 — Source data Fig. 3 [file 44318_2025_436_MOESM7_ESM.zip › Figure 3/3D/2022.07.18 GFP-LC3 IP_FSK DOSE _0003.tif]

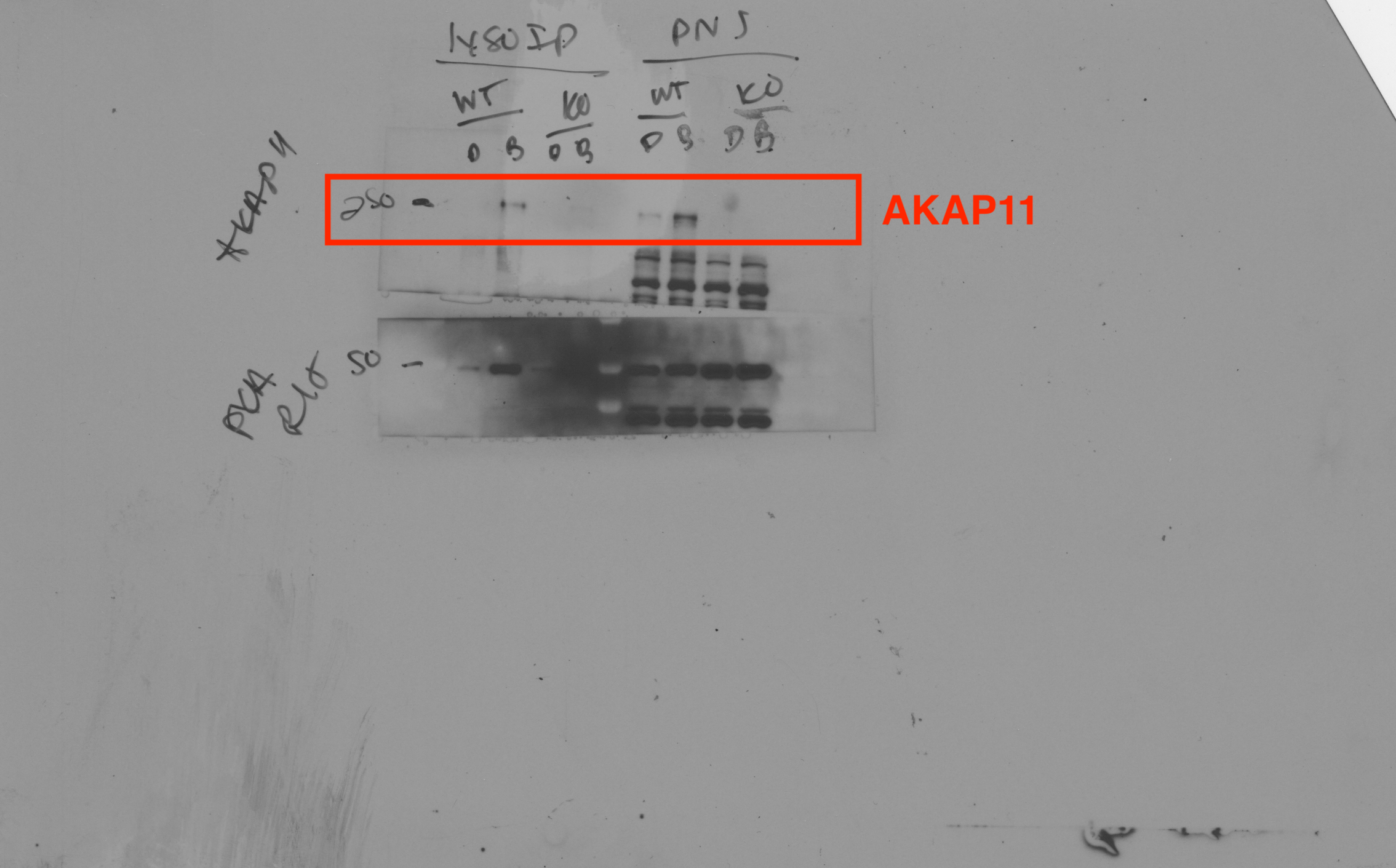

Supplement: Supplementary file 7 — Source data Fig. 3 [file 44318_2025_436_MOESM7_ESM.zip › Figure 3/3A/20241223_A11WTKO_lysoIP_0008.tif]

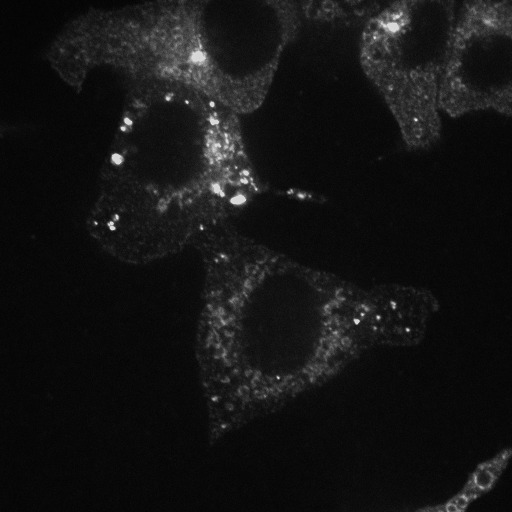

Supplement: Supplementary file 8 — Source data Fig. 4 [file 44318_2025_436_MOESM8_ESM.zip › Figure 4/4C/20230831_mngpkac1_A11ko_V5WT568_LAMP640_B_5.tif]

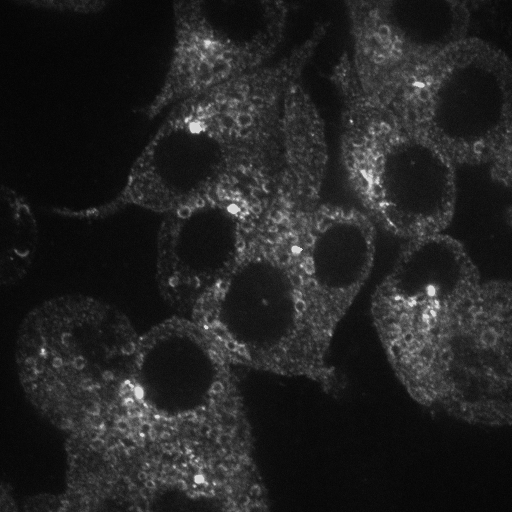

Supplement: Supplementary file 8 — Source data Fig. 4 [file 44318_2025_436_MOESM8_ESM.zip › Figure 4/4C/20230831_mngpkac1_A11ko_V5WT568_LAMP640_D_5.tif]

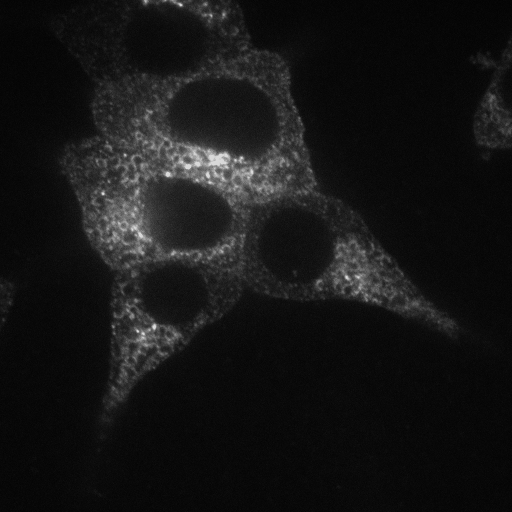

Supplement: Supplementary file 8 — Source data Fig. 4 [file 44318_2025_436_MOESM8_ESM.zip › Figure 4/4C/20230831_mngpkac1_A11ko_V5LIR568_LAMP640_D_7.tif]

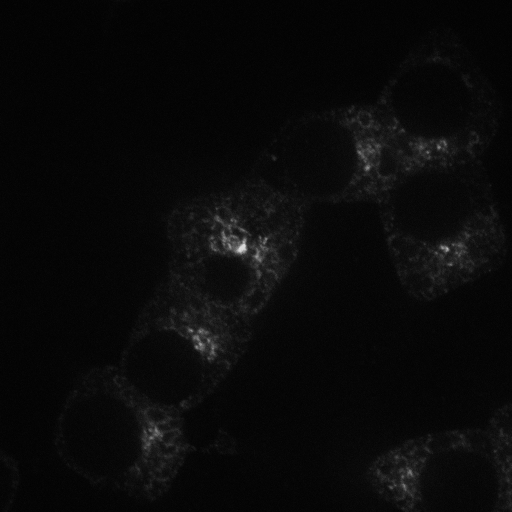

Supplement: Supplementary file 8 — Source data Fig. 4 [file 44318_2025_436_MOESM8_ESM.zip › Figure 4/4C/20230831_mngpkac1_NT_LAMP640_D_15.tif]

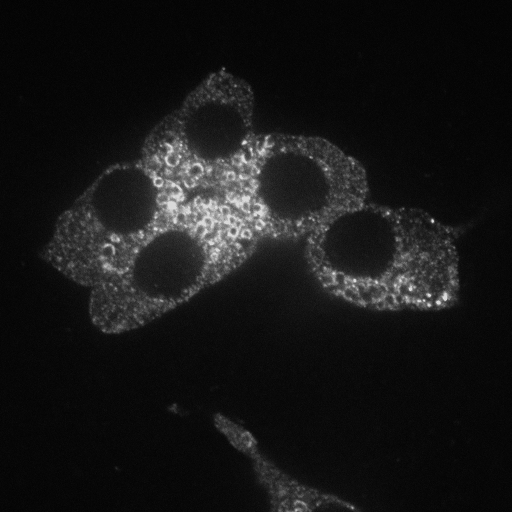

Supplement: Supplementary file 8 — Source data Fig. 4 [file 44318_2025_436_MOESM8_ESM.zip › Figure 4/4C/20230831_mngpkac1_A11ko_V5LIR568_LAMP640_B_9.tif]

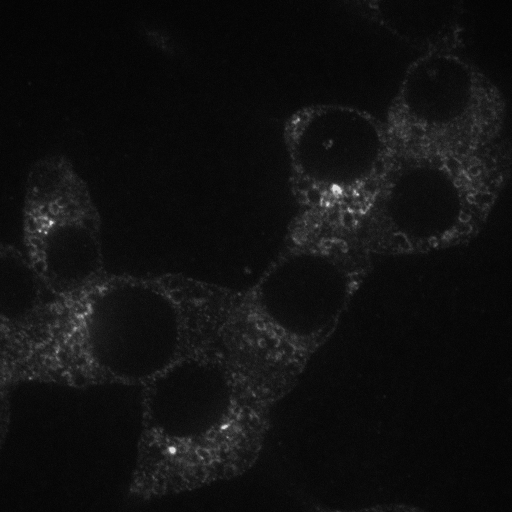

Supplement: Supplementary file 8 — Source data Fig. 4 [file 44318_2025_436_MOESM8_ESM.zip › Figure 4/4C/20230831_mngpkac1_A11ko_LAMP640_D_7.tif]

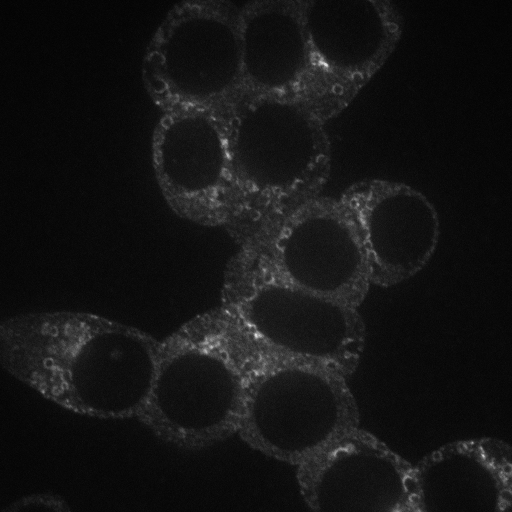

Supplement: Supplementary file 8 — Source data Fig. 4 [file 44318_2025_436_MOESM8_ESM.zip › Figure 4/4C/20230831_mngpkac1_A11ko_LAMP640_B_3.tif]

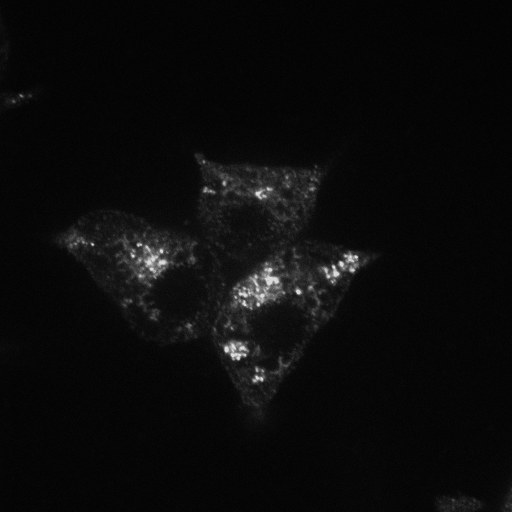

Supplement: Supplementary file 8 — Source data Fig. 4 [file 44318_2025_436_MOESM8_ESM.zip › Figure 4/4C/20230831_mngpkac1_NT_LAMP640_B_19.tif]

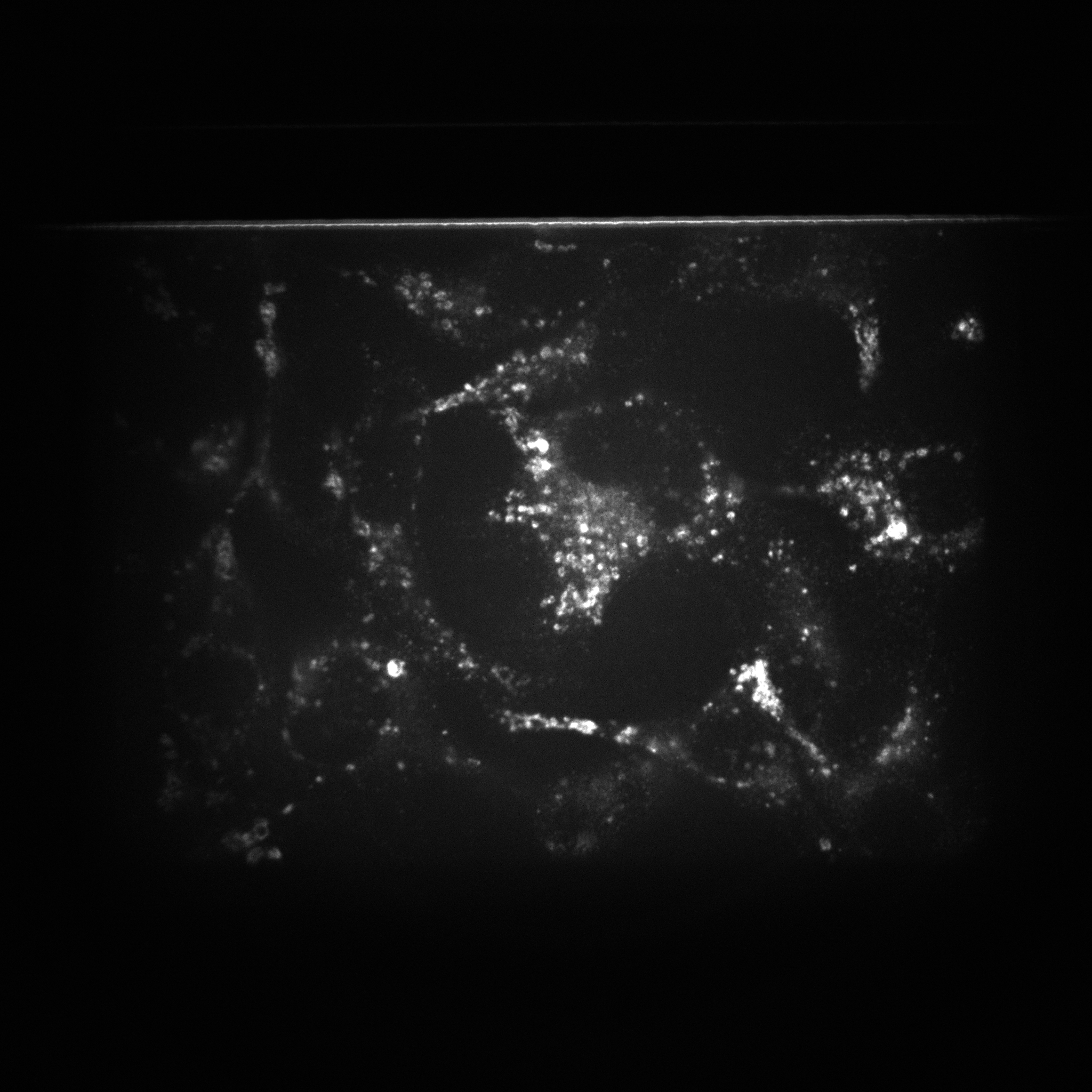

Supplement: Supplementary file 8 — Source data Fig. 4 [file 44318_2025_436_MOESM8_ESM.zip › Figure 4/4A/AKAP11_WT_BafA_3_20230825_63208 PM/AKAP11_WT_BafA_3_w0003.tif]

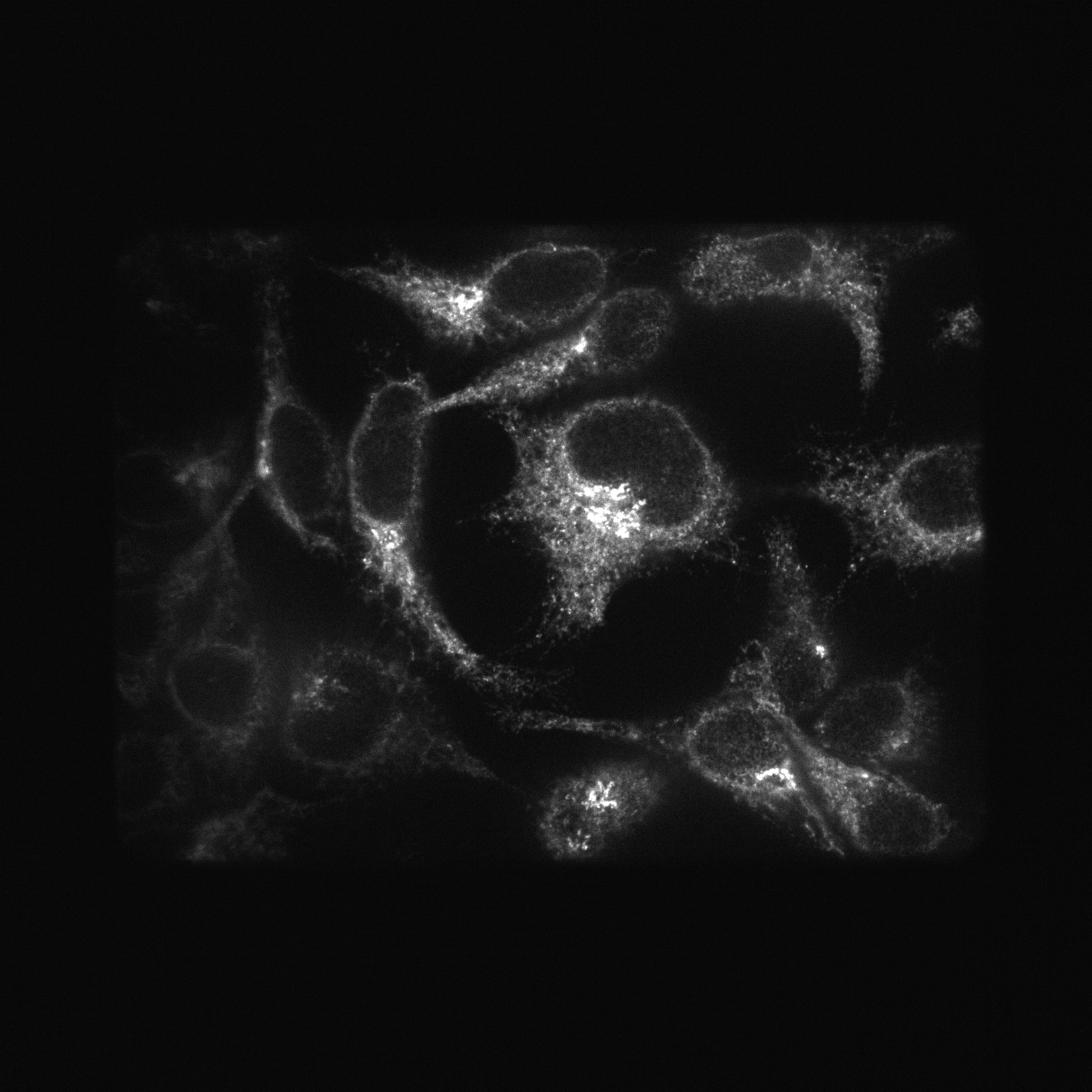

Supplement: Supplementary file 8 — Source data Fig. 4 [file 44318_2025_436_MOESM8_ESM.zip › Figure 4/4A/AKAP11_WT_BafA_3_20230825_63208 PM/AKAP11_WT_BafA_3_w0002.tif]

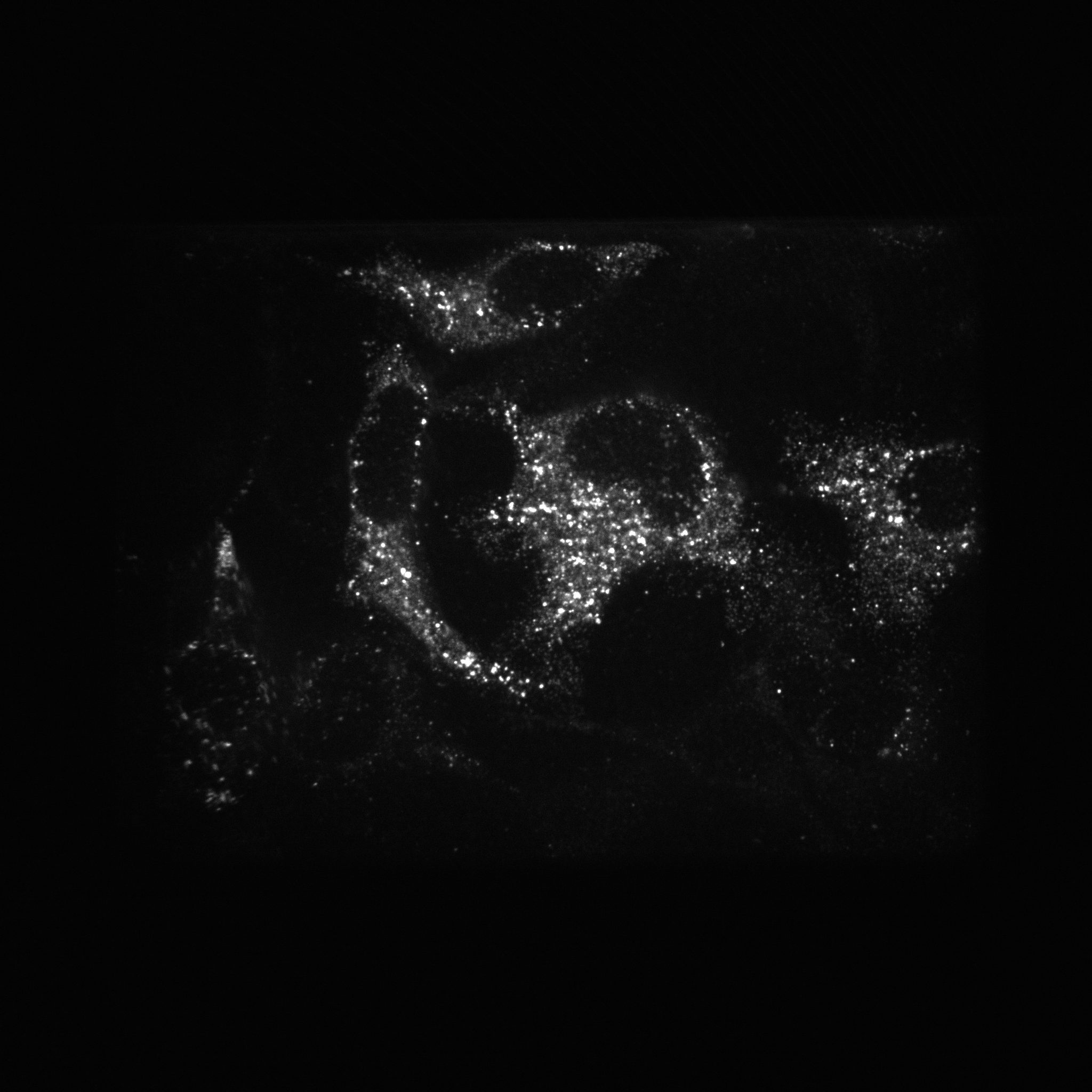

Supplement: Supplementary file 8 — Source data Fig. 4 [file 44318_2025_436_MOESM8_ESM.zip › Figure 4/4A/AKAP11_WT_BafA_3_20230825_63208 PM/AKAP11_WT_BafA_3_w0000.tif]

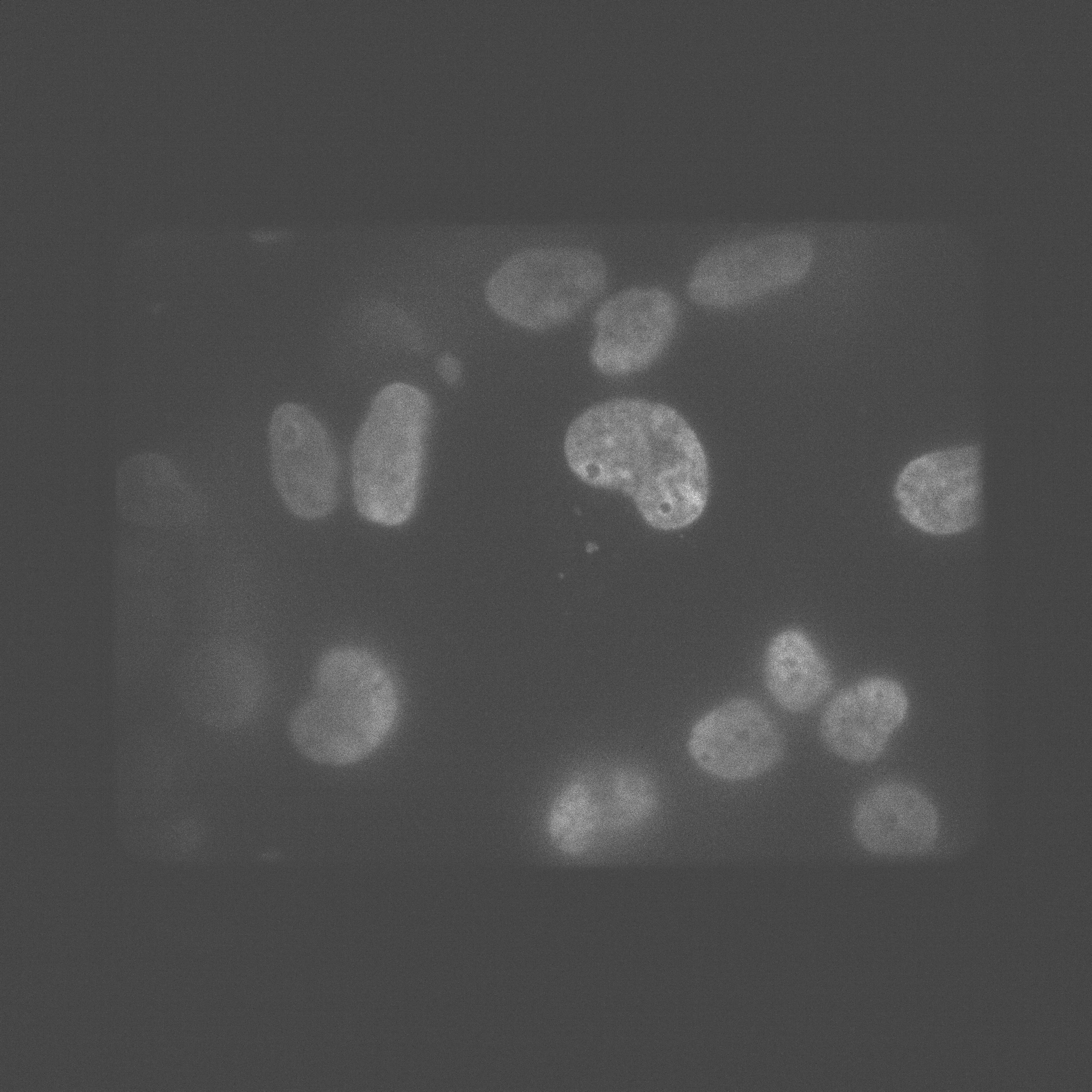

Supplement: Supplementary file 8 — Source data Fig. 4 [file 44318_2025_436_MOESM8_ESM.zip › Figure 4/4A/AKAP11_WT_BafA_3_20230825_63208 PM/AKAP11_WT_BafA_3_w0001.tif]

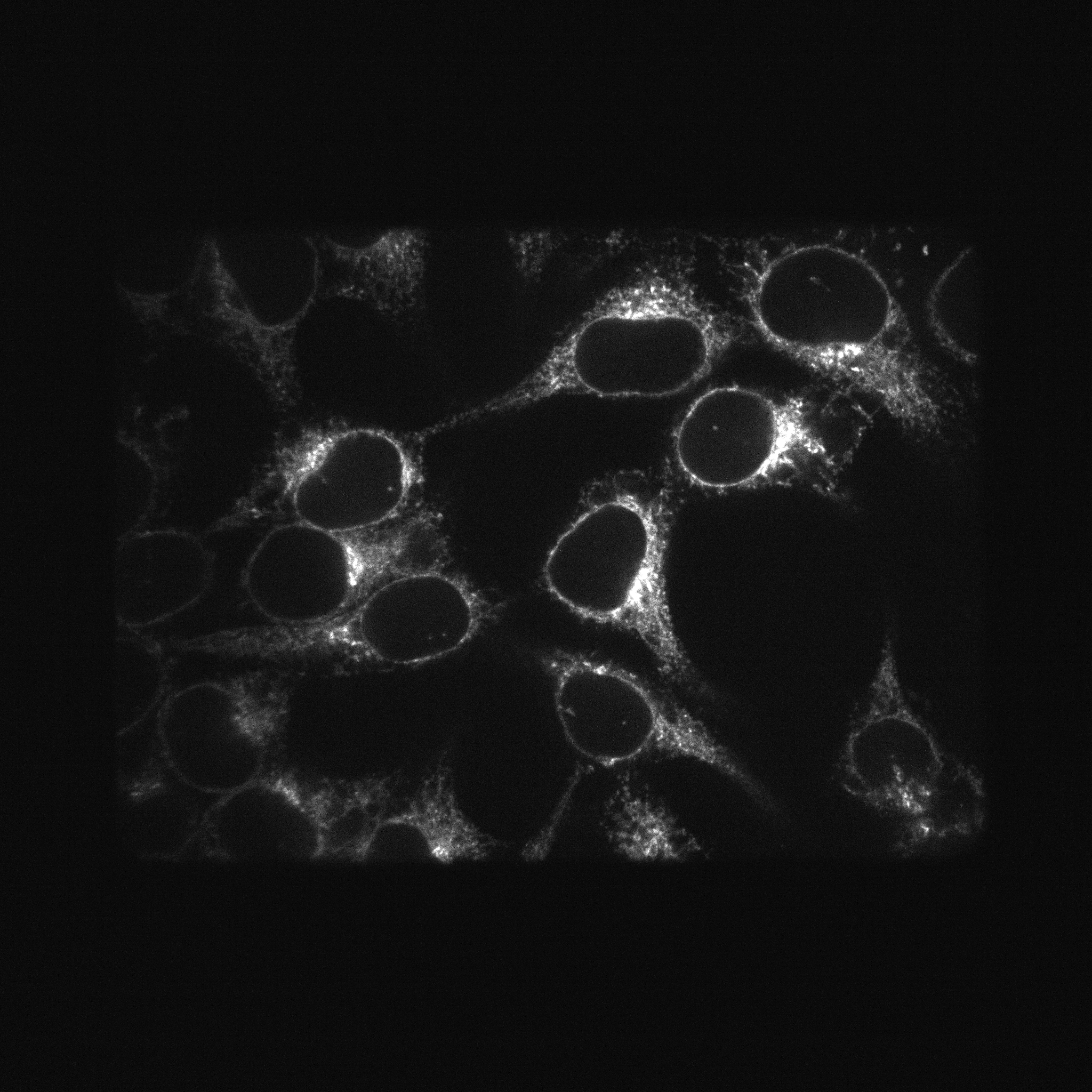

Supplement: Supplementary file 8 — Source data Fig. 4 [file 44318_2025_436_MOESM8_ESM.zip › Figure 4/4A/AKAP11_WT_dmso_2_20230825_62353 PM/AKAP11_WT_dmso_2_w0002.tif]

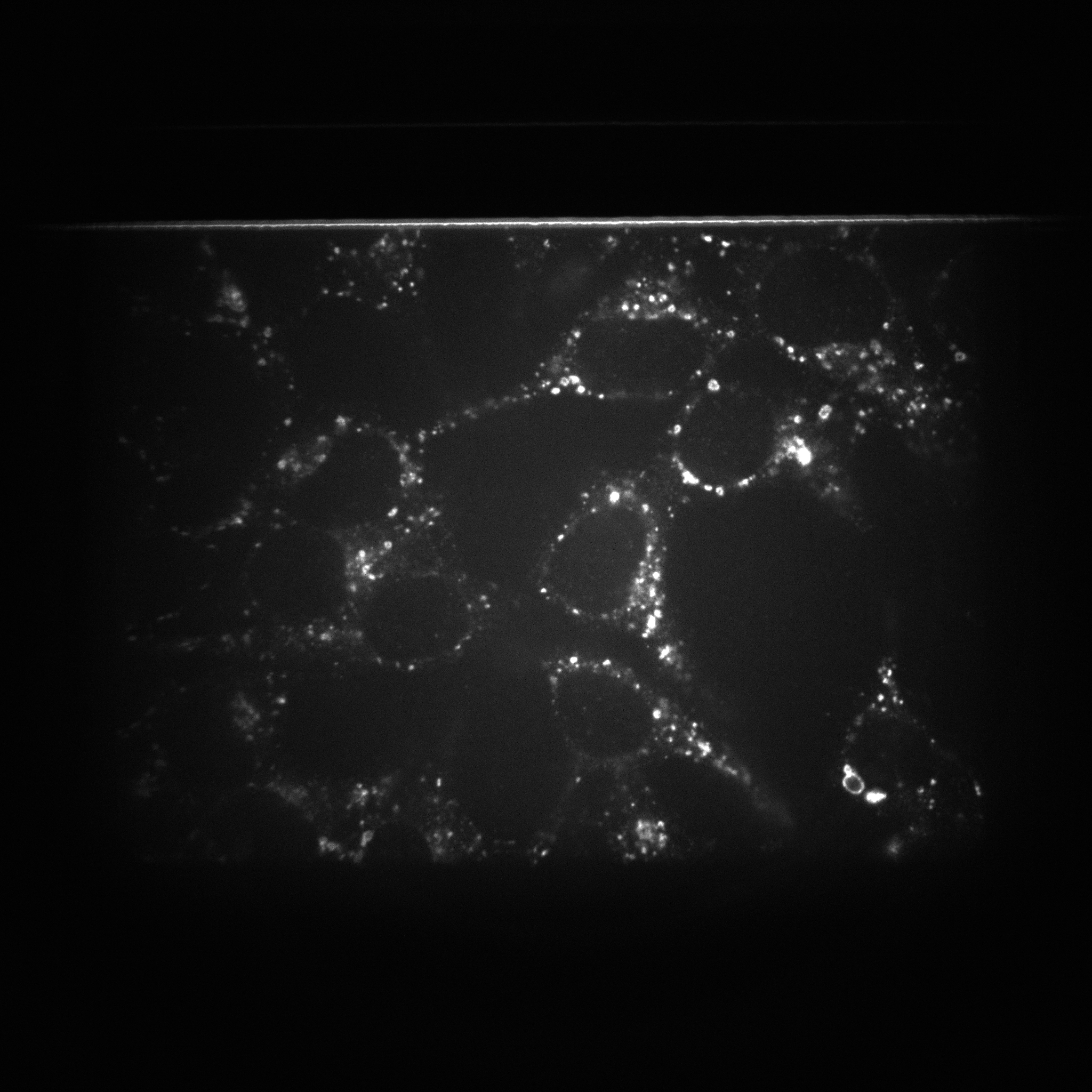

Supplement: Supplementary file 8 — Source data Fig. 4 [file 44318_2025_436_MOESM8_ESM.zip › Figure 4/4A/AKAP11_WT_dmso_2_20230825_62353 PM/AKAP11_WT_dmso_2_w0003.tif]

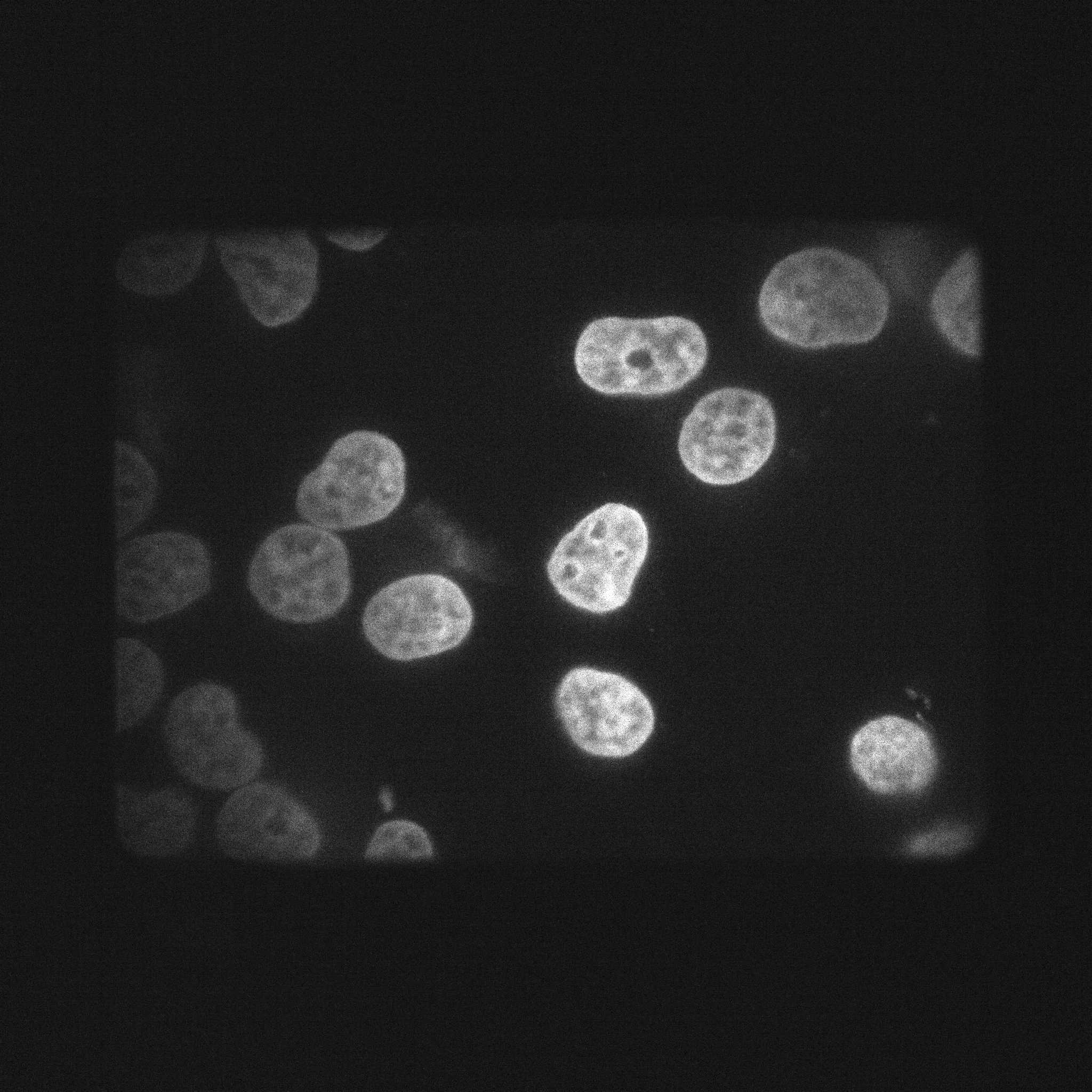

Supplement: Supplementary file 8 — Source data Fig. 4 [file 44318_2025_436_MOESM8_ESM.zip › Figure 4/4A/AKAP11_WT_dmso_2_20230825_62353 PM/AKAP11_WT_dmso_2_w0001.tif]

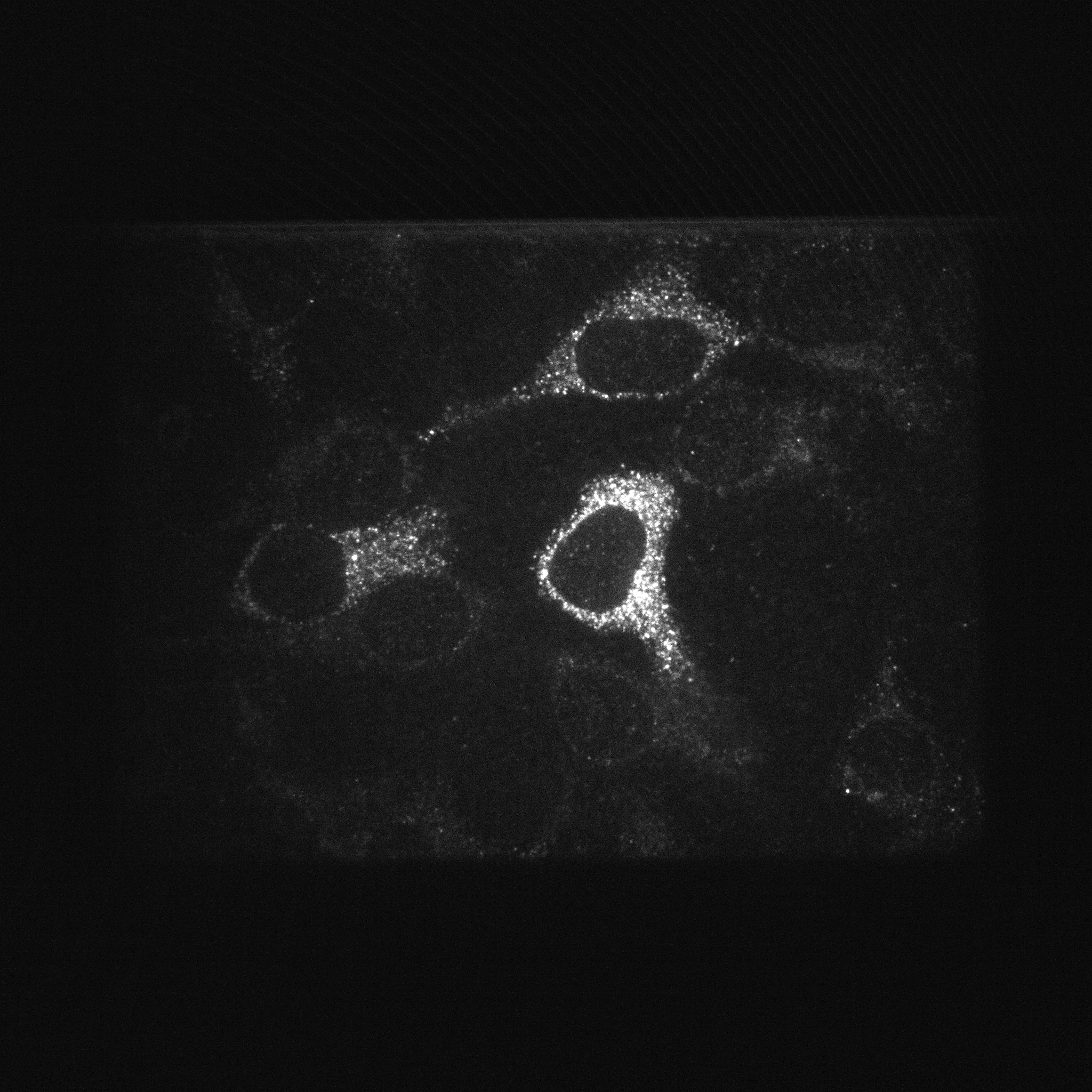

Supplement: Supplementary file 8 — Source data Fig. 4 [file 44318_2025_436_MOESM8_ESM.zip › Figure 4/4A/AKAP11_WT_dmso_2_20230825_62353 PM/AKAP11_WT_dmso_2_w0000.tif]

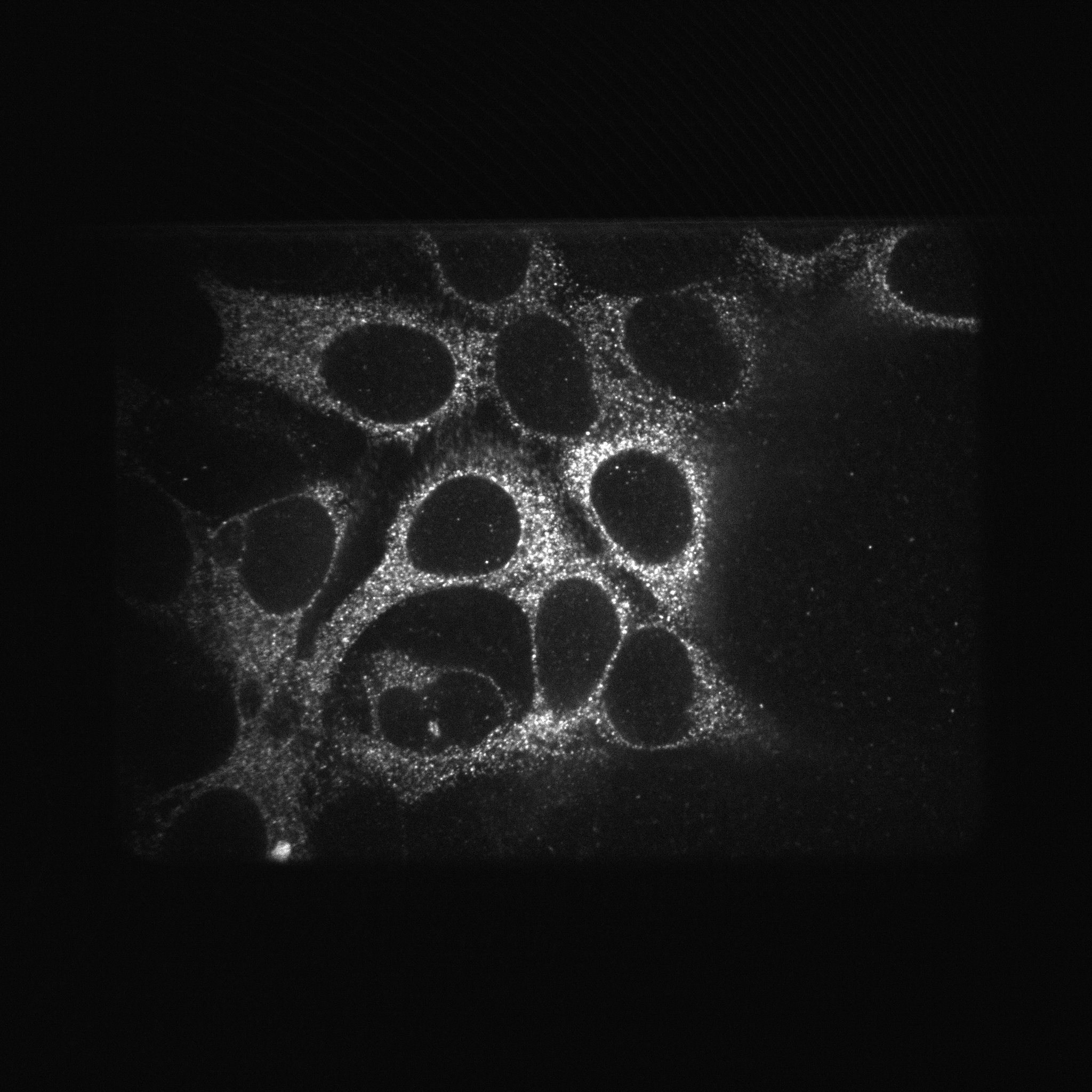

Supplement: Supplementary file 8 — Source data Fig. 4 [file 44318_2025_436_MOESM8_ESM.zip › Figure 4/4A/AKAP11_dLL_dmso_5_20230825_64946 PM/AKAP11_dLL_dmso_5_w0000.tif]

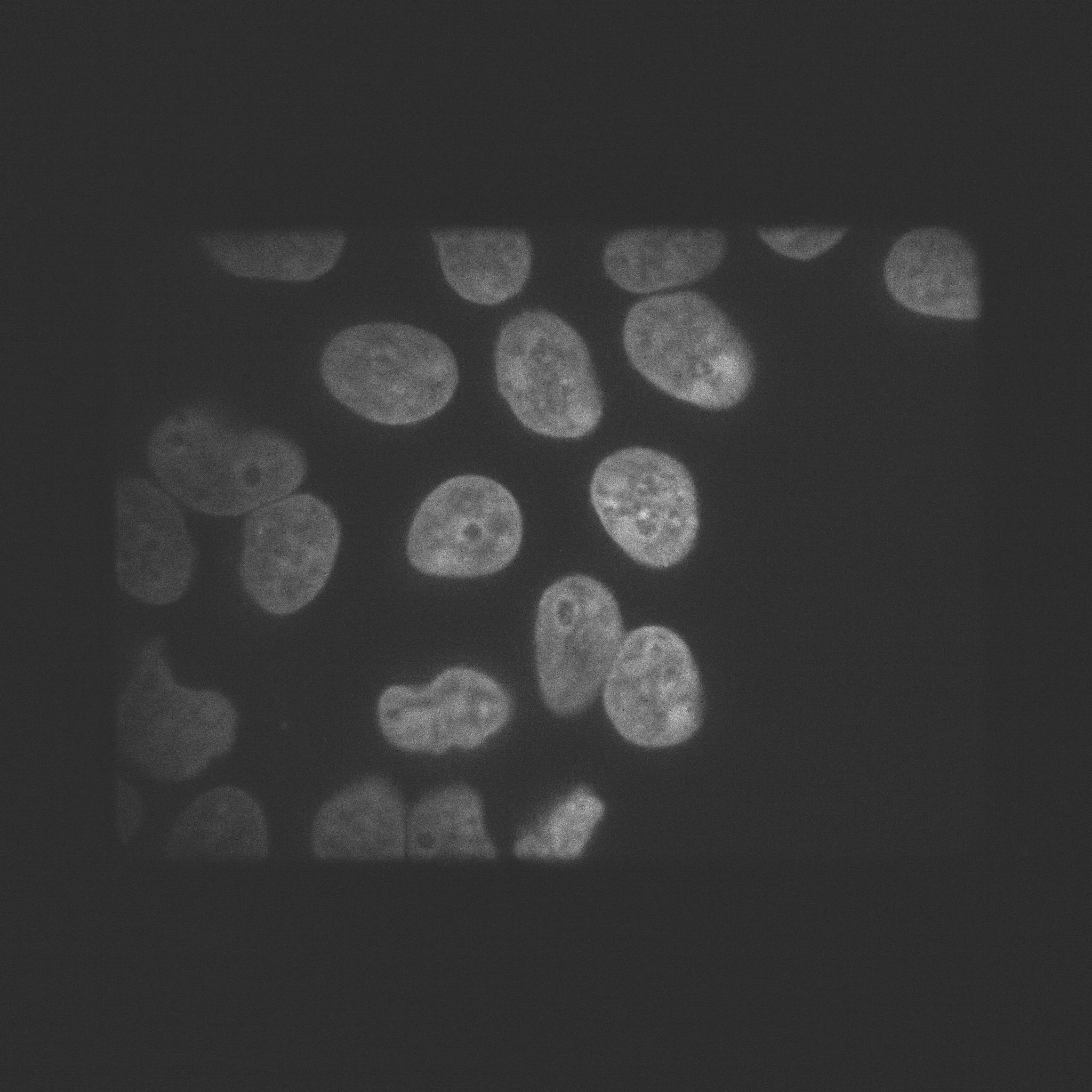

Supplement: Supplementary file 8 — Source data Fig. 4 [file 44318_2025_436_MOESM8_ESM.zip › Figure 4/4A/AKAP11_dLL_dmso_5_20230825_64946 PM/AKAP11_dLL_dmso_5_w0001.tif]

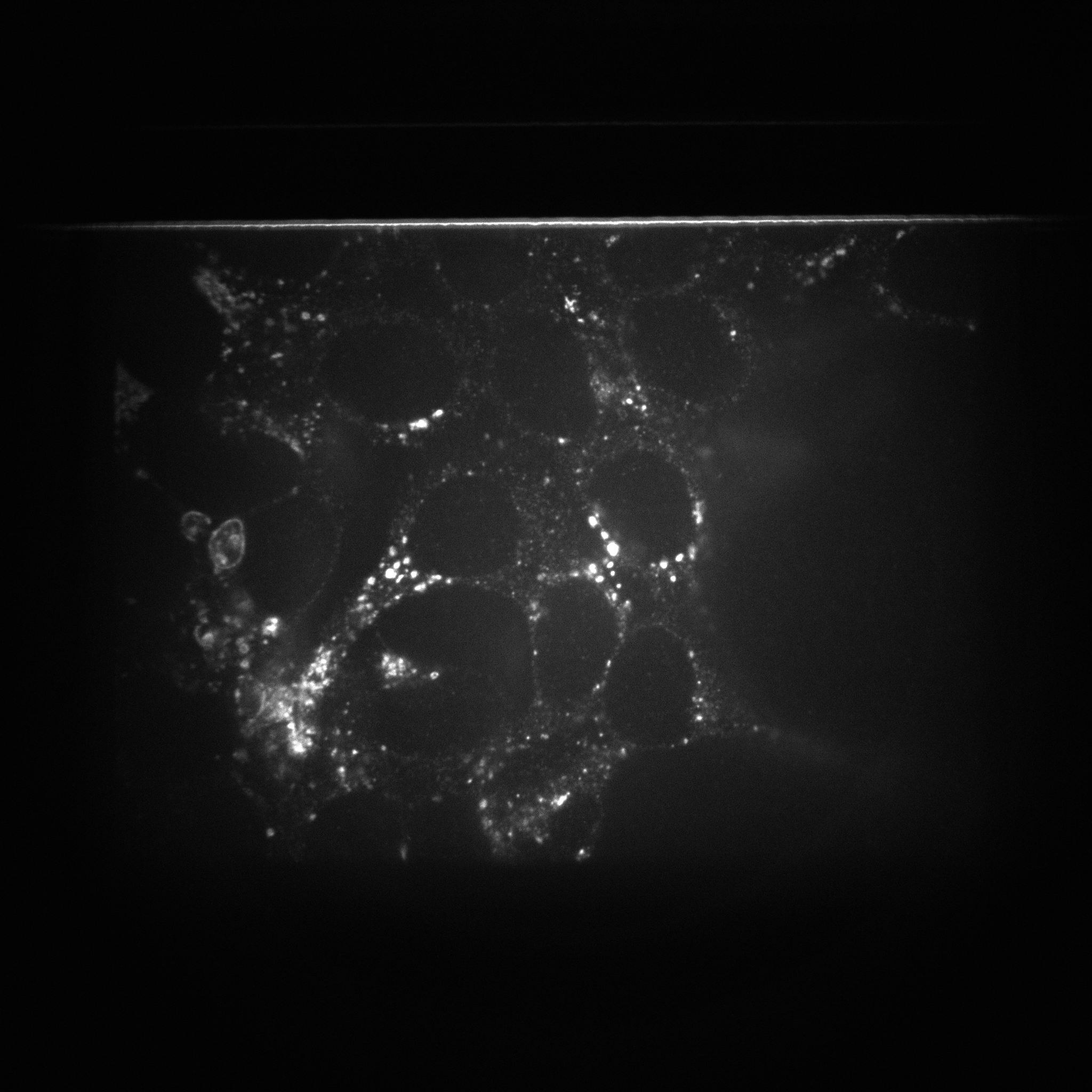

Supplement: Supplementary file 8 — Source data Fig. 4 [file 44318_2025_436_MOESM8_ESM.zip › Figure 4/4A/AKAP11_dLL_dmso_5_20230825_64946 PM/AKAP11_dLL_dmso_5_w0003.tif]

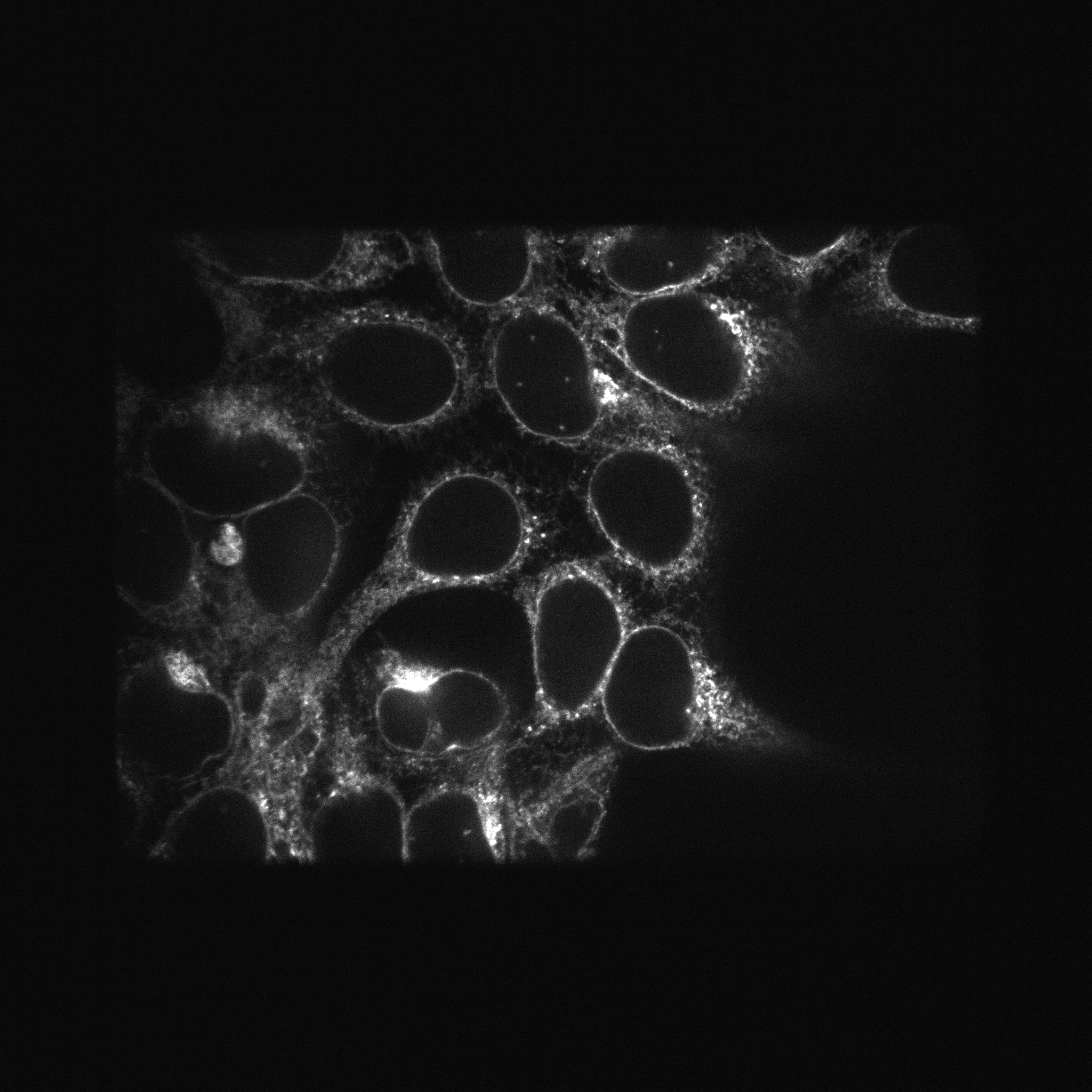

Supplement: Supplementary file 8 — Source data Fig. 4 [file 44318_2025_436_MOESM8_ESM.zip › Figure 4/4A/AKAP11_dLL_dmso_5_20230825_64946 PM/AKAP11_dLL_dmso_5_w0002.tif]

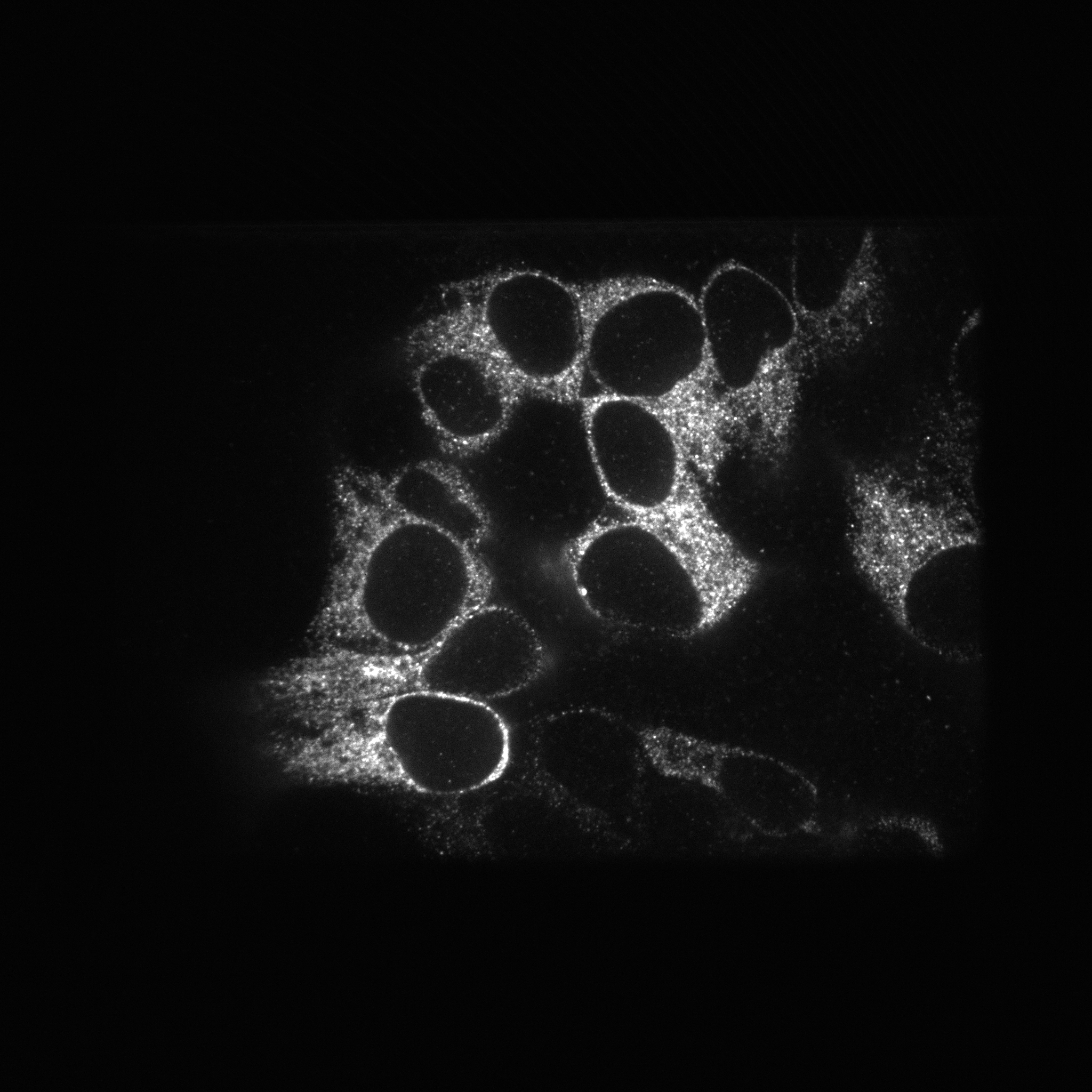

Supplement: Supplementary file 8 — Source data Fig. 4 [file 44318_2025_436_MOESM8_ESM.zip › Figure 4/4A/AKAP11_dLL_BafA_2_20230825_65210 PM/AKAP11_dLL_BafA_2_w0000.tif]

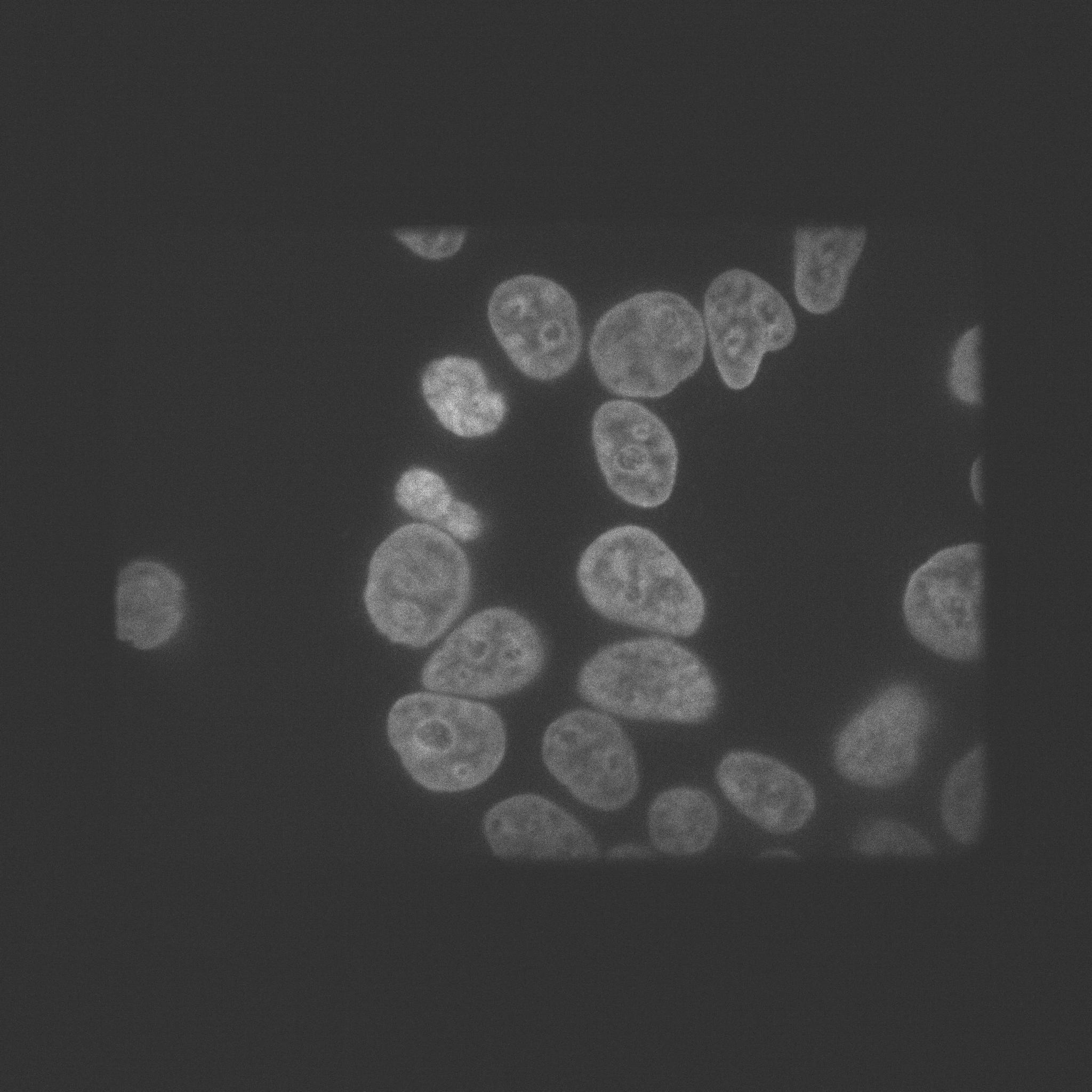

Supplement: Supplementary file 8 — Source data Fig. 4 [file 44318_2025_436_MOESM8_ESM.zip › Figure 4/4A/AKAP11_dLL_BafA_2_20230825_65210 PM/AKAP11_dLL_BafA_2_w0001.tif]

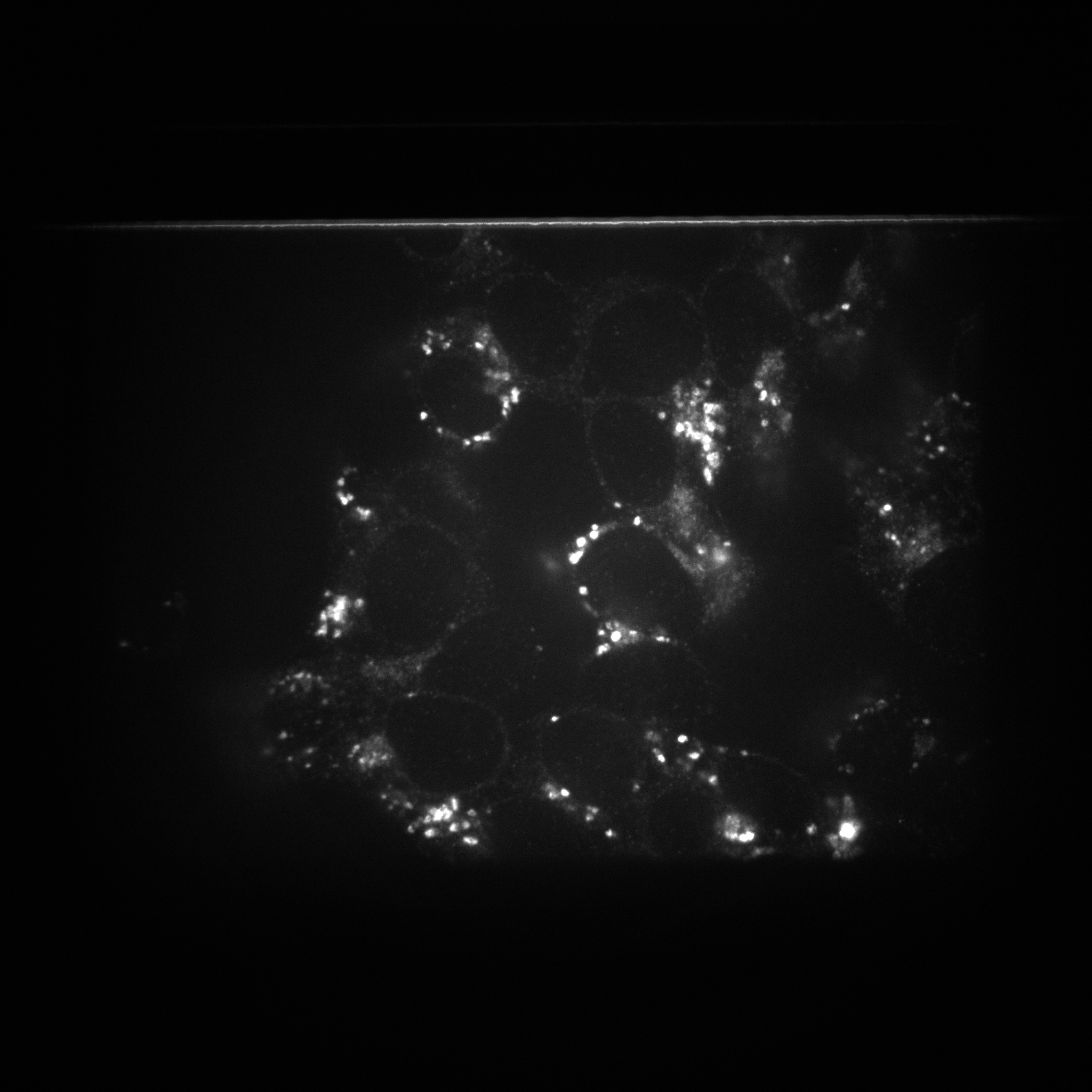

Supplement: Supplementary file 8 — Source data Fig. 4 [file 44318_2025_436_MOESM8_ESM.zip › Figure 4/4A/AKAP11_dLL_BafA_2_20230825_65210 PM/AKAP11_dLL_BafA_2_w0003.tif]

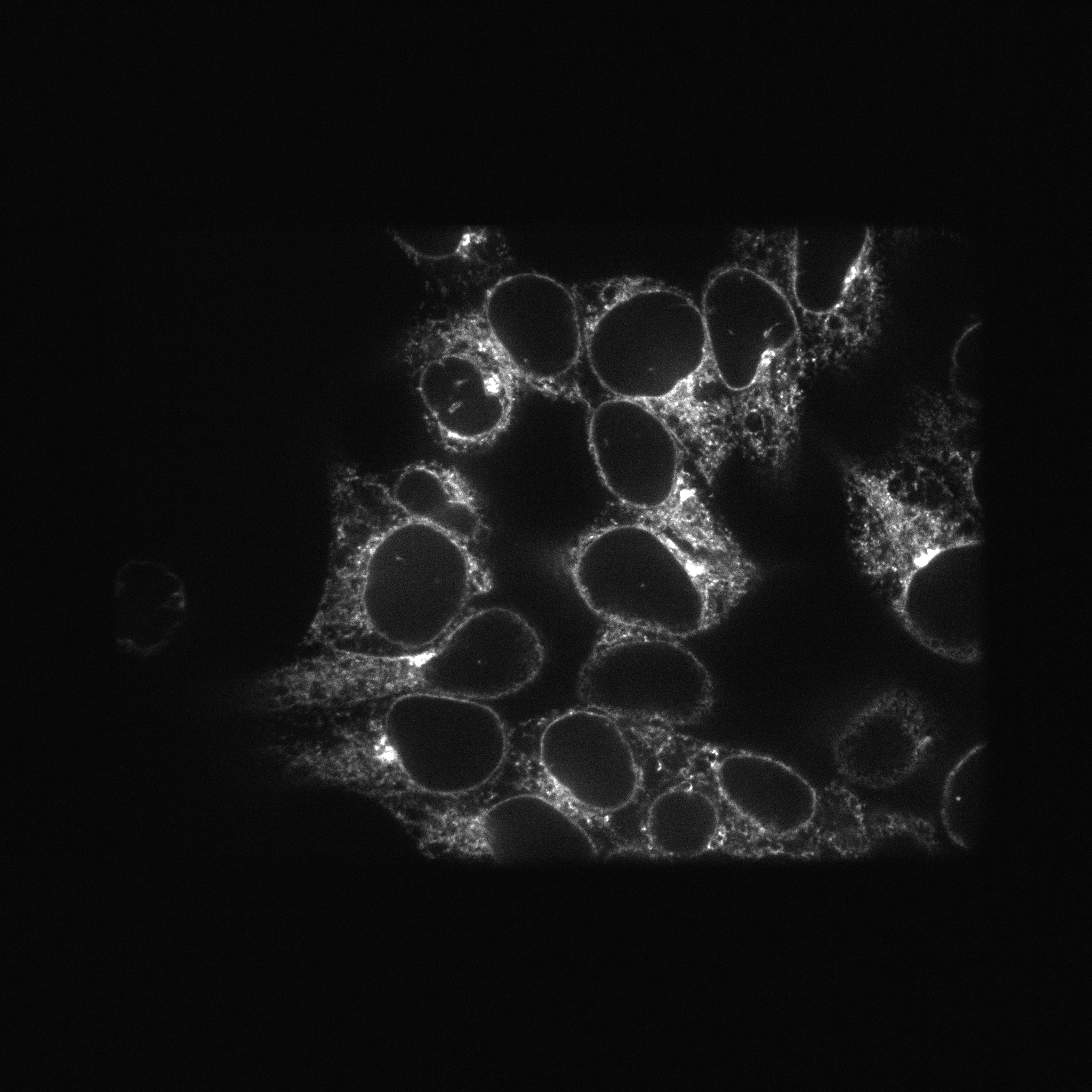

Supplement: Supplementary file 8 — Source data Fig. 4 [file 44318_2025_436_MOESM8_ESM.zip › Figure 4/4A/AKAP11_dLL_BafA_2_20230825_65210 PM/AKAP11_dLL_BafA_2_w0002.tif]

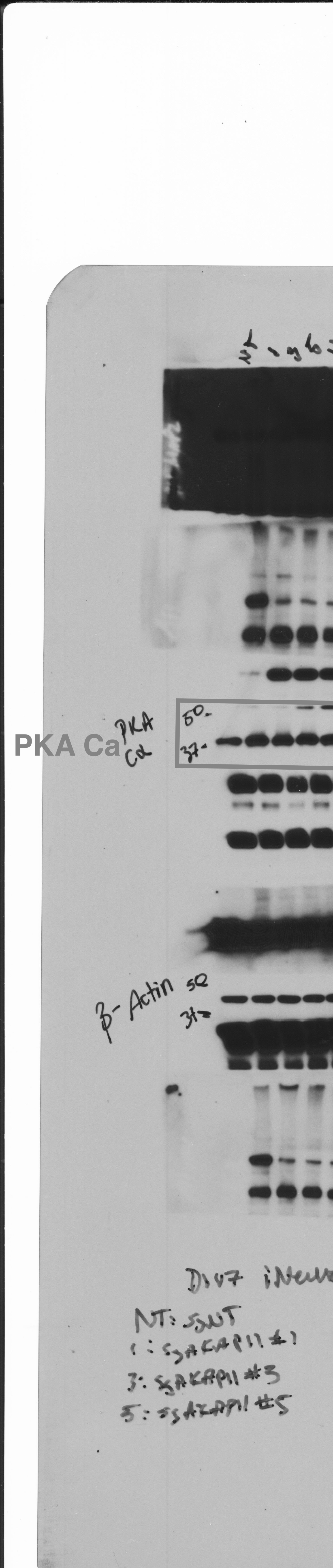

Supplement: Supplementary file 9 — Source data Fig. 5 [file 44318_2025_436_MOESM9_ESM.zip › Figure 5/5A/AMRscan2.jpg]

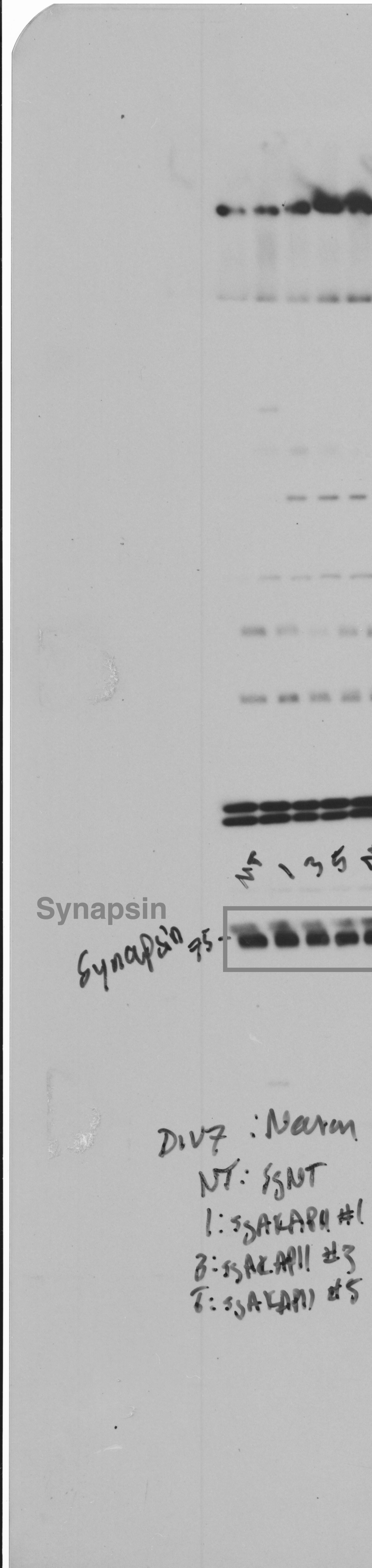

Supplement: Supplementary file 9 — Source data Fig. 5 [file 44318_2025_436_MOESM9_ESM.zip › Figure 5/5A/AMRscan3.jpg]

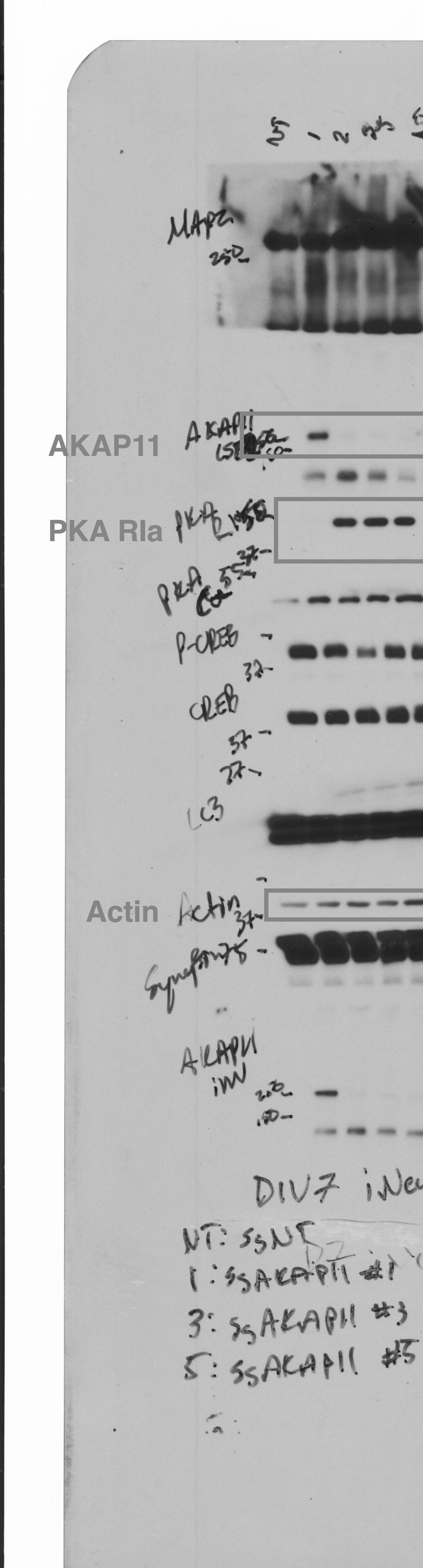

Supplement: Supplementary file 9 — Source data Fig. 5 [file 44318_2025_436_MOESM9_ESM.zip › Figure 5/5A/AMRscan1.jpg]

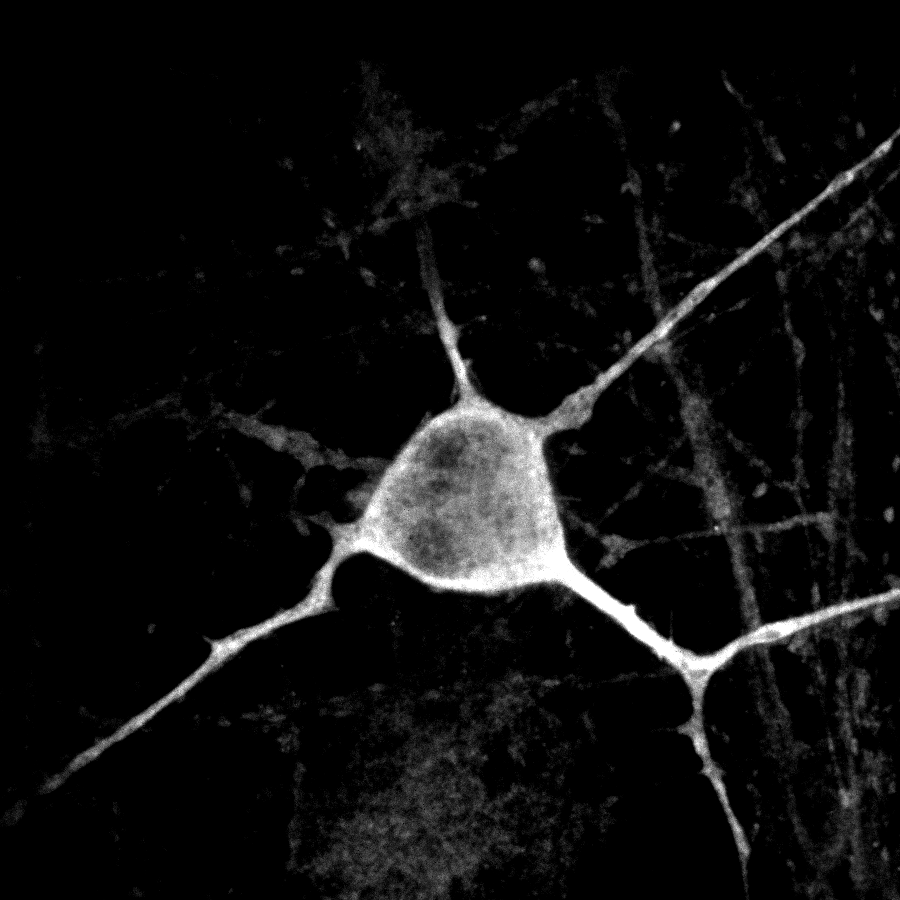

Supplement: Supplementary file 9 — Source data Fig. 5 [file 44318_2025_436_MOESM9_ESM.zip › Figure 5/5B/sgAKAP11 1/sgAKAP11.1composite2.tif]

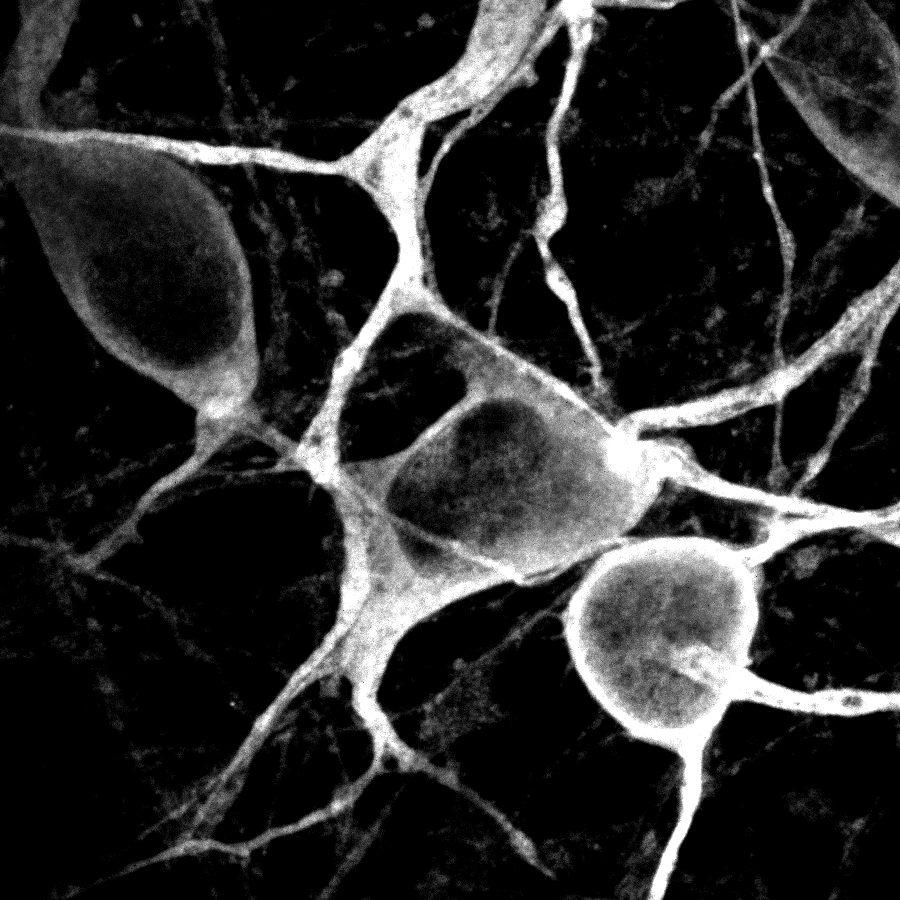

Supplement: Supplementary file 9 — Source data Fig. 5 [file 44318_2025_436_MOESM9_ESM.zip › Figure 5/5B/sgNT/sgNTcomposite3 copy.tif]

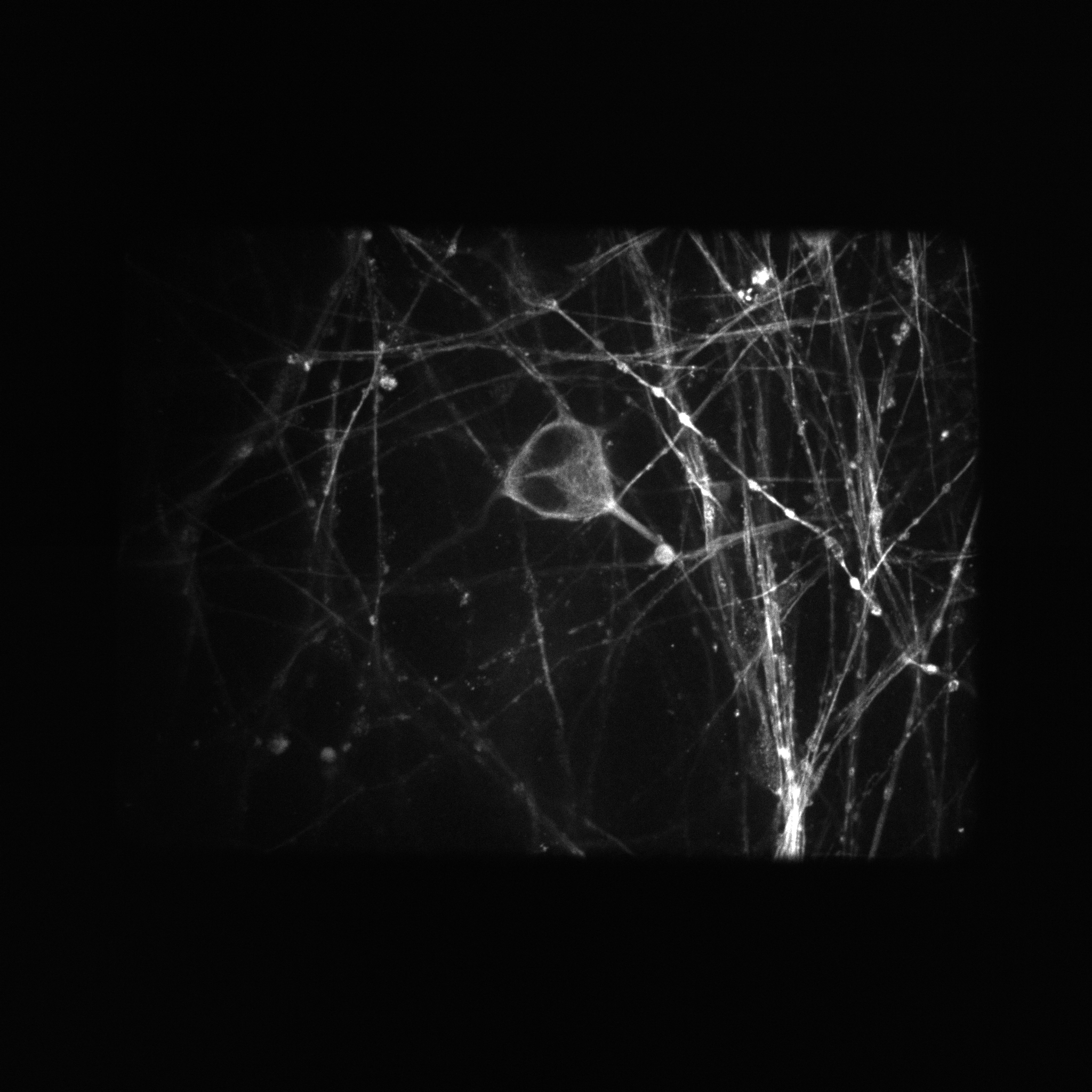

Supplement: Supplementary file 9 — Source data Fig. 5 [file 44318_2025_436_MOESM9_ESM.zip › Figure 5/5B/sgAKAP11 1/A11 1 tuj1map22_20240620_110012 AM/A11 1 tuj1map22_w0001_z0003.tif]

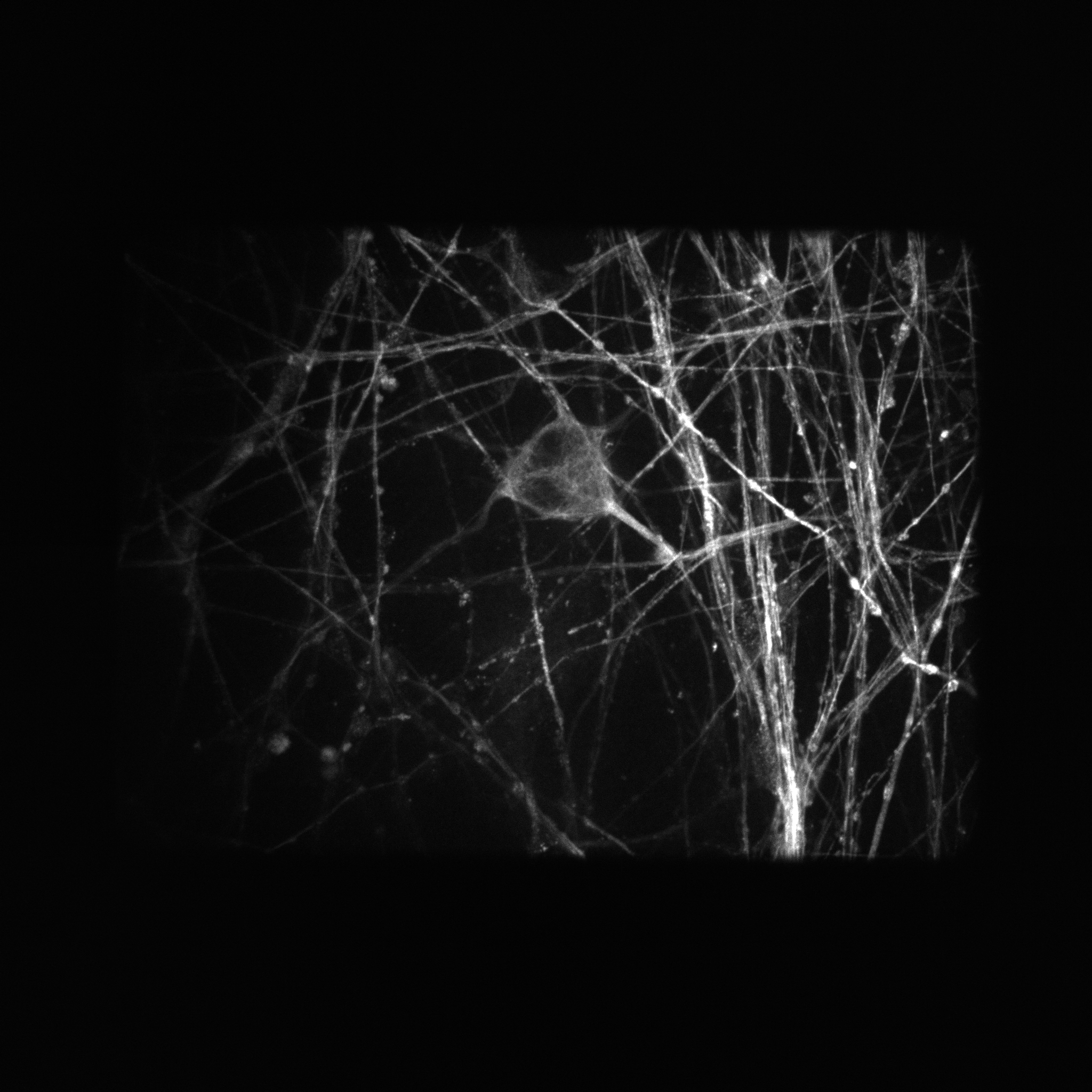

Supplement: Supplementary file 9 — Source data Fig. 5 [file 44318_2025_436_MOESM9_ESM.zip › Figure 5/5B/sgAKAP11 1/A11 1 tuj1map22_20240620_110012 AM/A11 1 tuj1map22_w0001_z0002.tif]

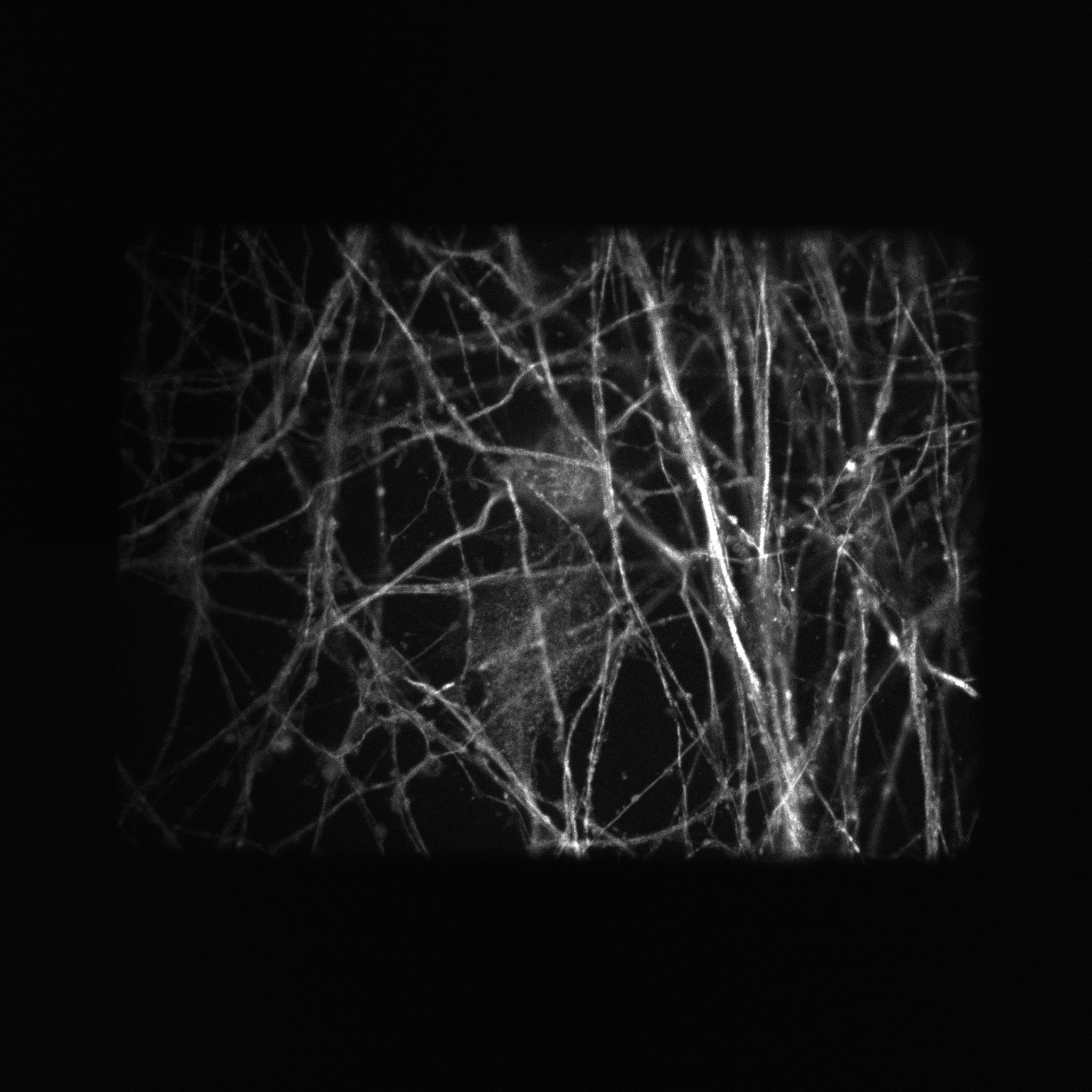

Supplement: Supplementary file 9 — Source data Fig. 5 [file 44318_2025_436_MOESM9_ESM.zip › Figure 5/5B/sgAKAP11 1/A11 1 tuj1map22_20240620_110012 AM/A11 1 tuj1map22_w0001_z0000.tif]

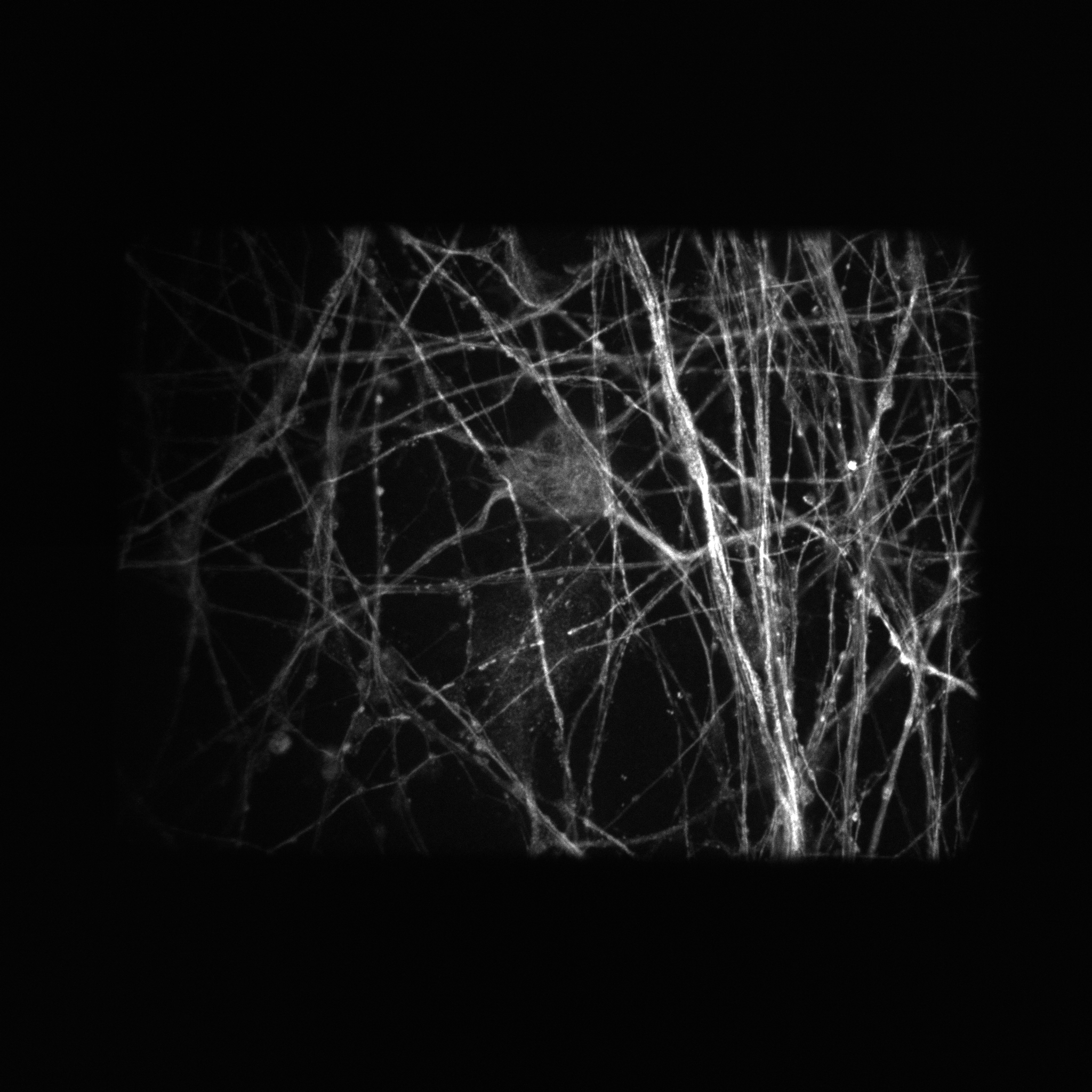

Supplement: Supplementary file 9 — Source data Fig. 5 [file 44318_2025_436_MOESM9_ESM.zip › Figure 5/5B/sgAKAP11 1/A11 1 tuj1map22_20240620_110012 AM/A11 1 tuj1map22_w0001_z0001.tif]

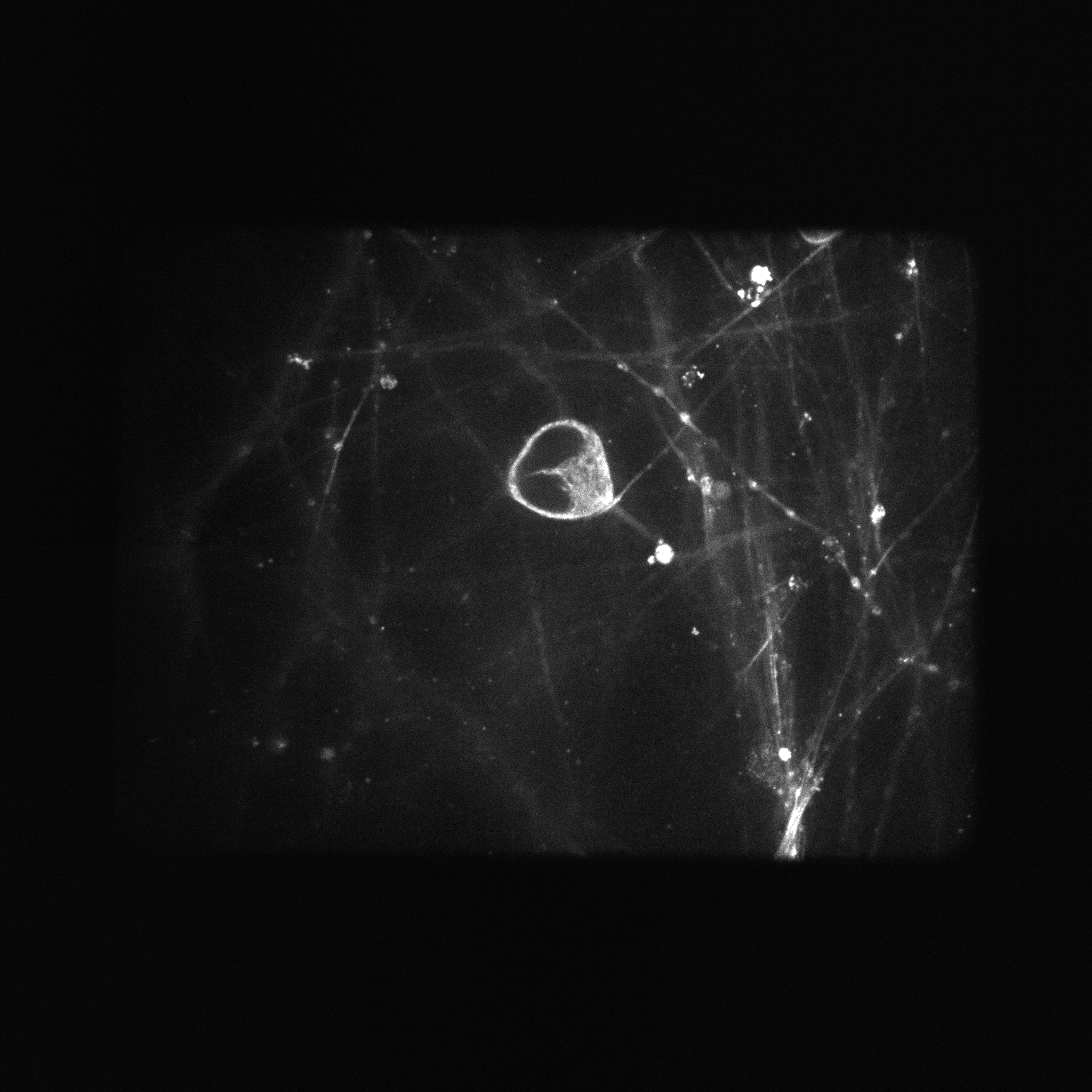

Supplement: Supplementary file 9 — Source data Fig. 5 [file 44318_2025_436_MOESM9_ESM.zip › Figure 5/5B/sgAKAP11 1/A11 1 tuj1map22_20240620_110012 AM/A11 1 tuj1map22_w0001_z0005.tif]

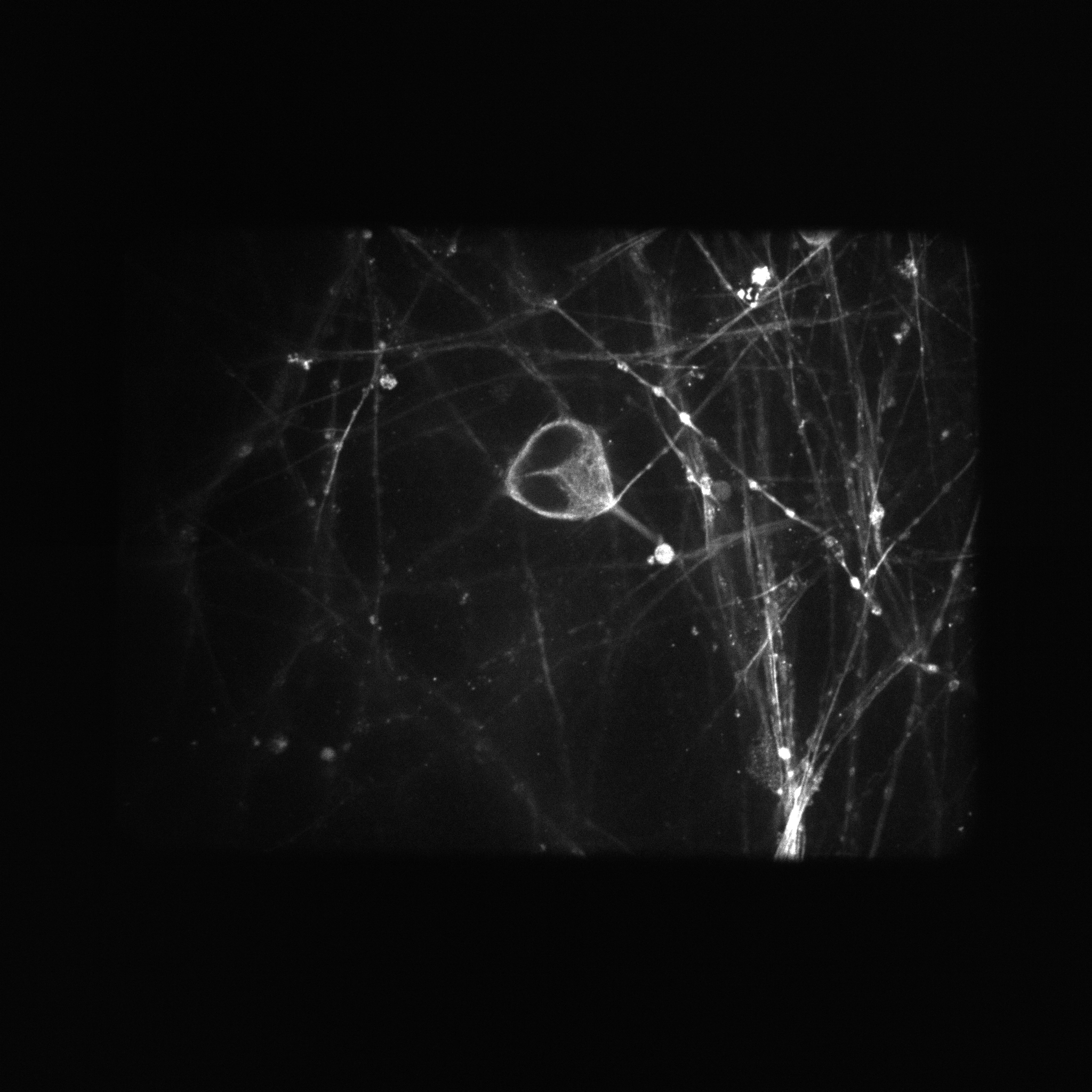

Supplement: Supplementary file 9 — Source data Fig. 5 [file 44318_2025_436_MOESM9_ESM.zip › Figure 5/5B/sgAKAP11 1/A11 1 tuj1map22_20240620_110012 AM/A11 1 tuj1map22_w0001_z0004.tif]

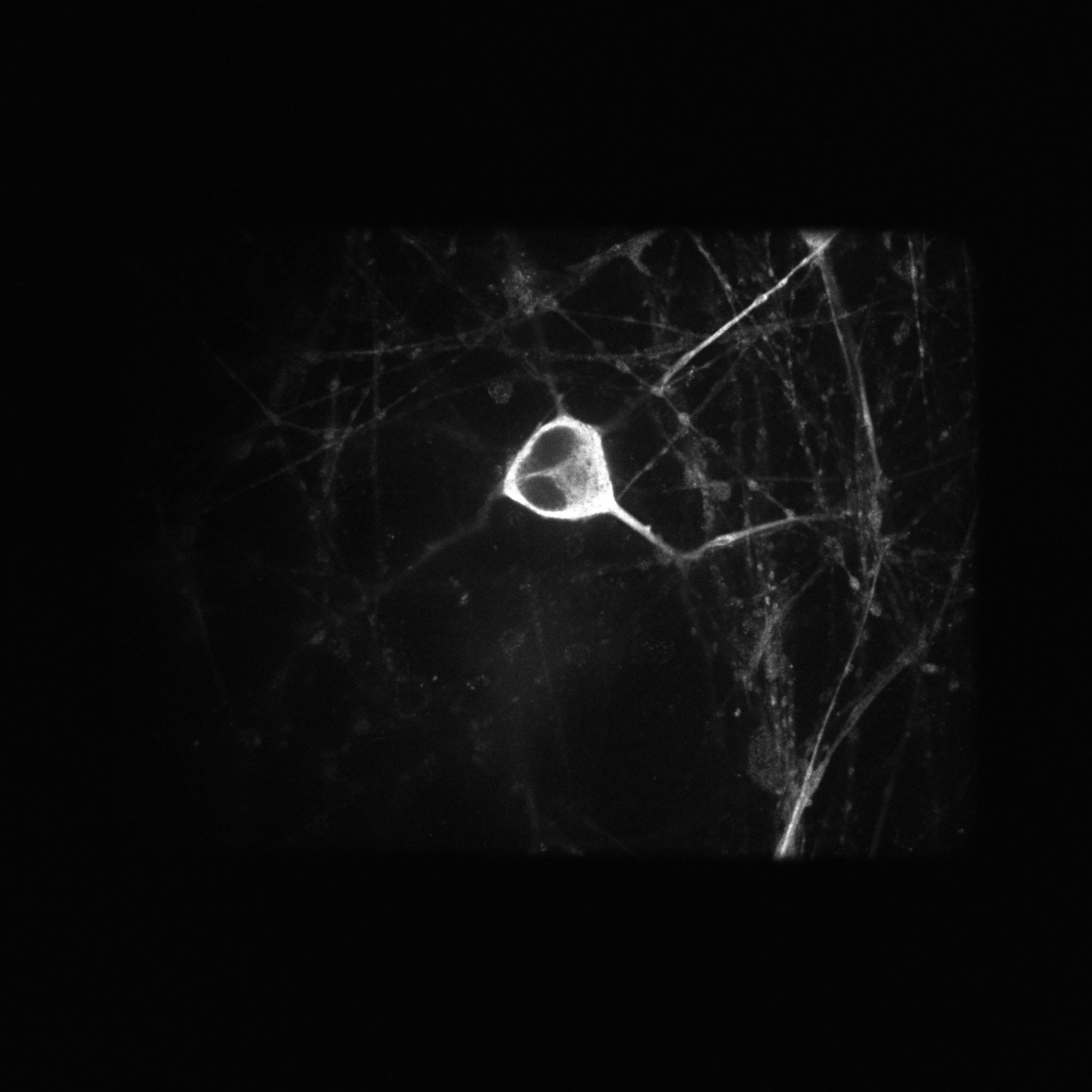

Supplement: Supplementary file 9 — Source data Fig. 5 [file 44318_2025_436_MOESM9_ESM.zip › Figure 5/5B/sgAKAP11 1/A11 1 tuj1map22_20240620_110012 AM/A11 1 tuj1map22_w0000_z0003.tif]

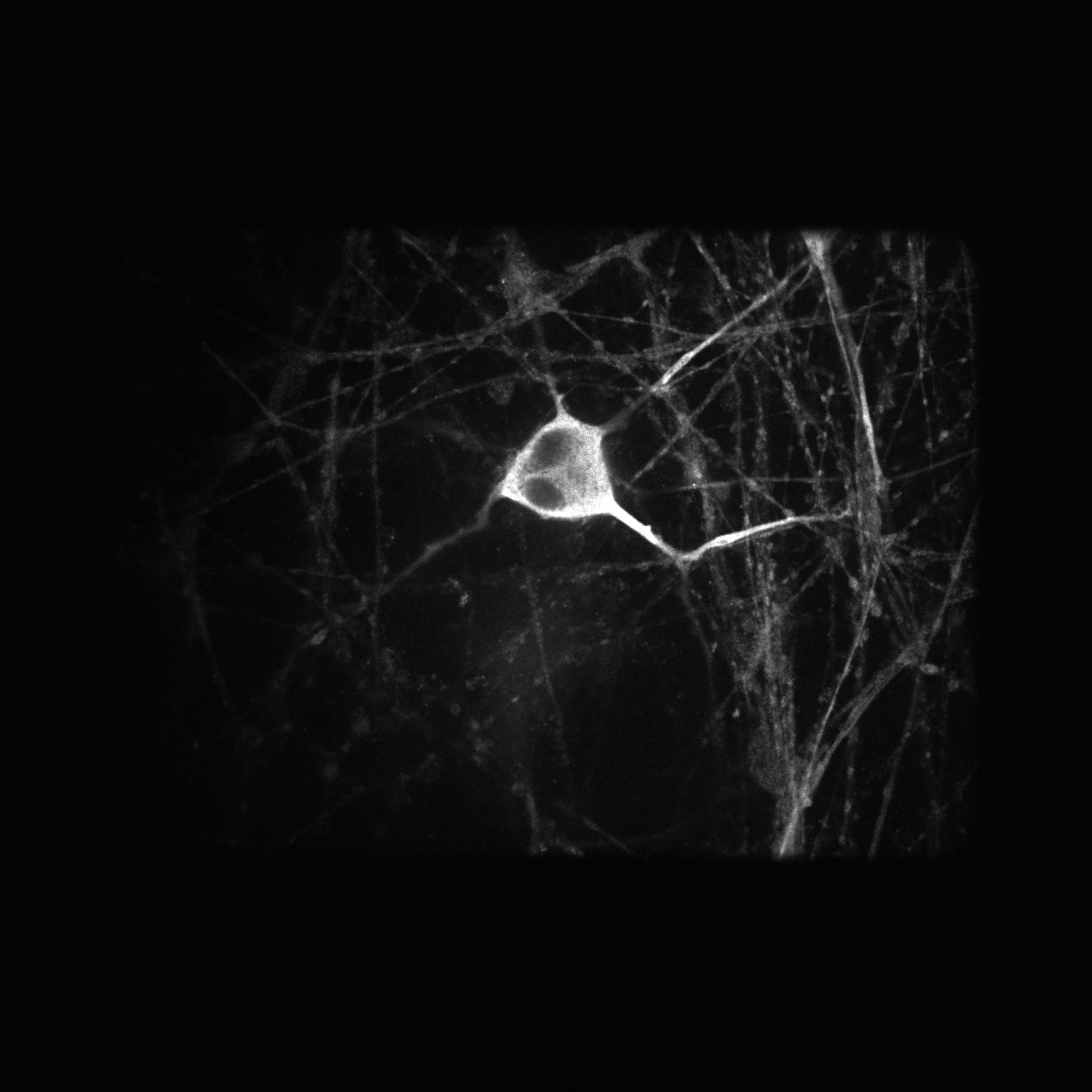

Supplement: Supplementary file 9 — Source data Fig. 5 [file 44318_2025_436_MOESM9_ESM.zip › Figure 5/5B/sgAKAP11 1/A11 1 tuj1map22_20240620_110012 AM/A11 1 tuj1map22_w0000_z0002.tif]

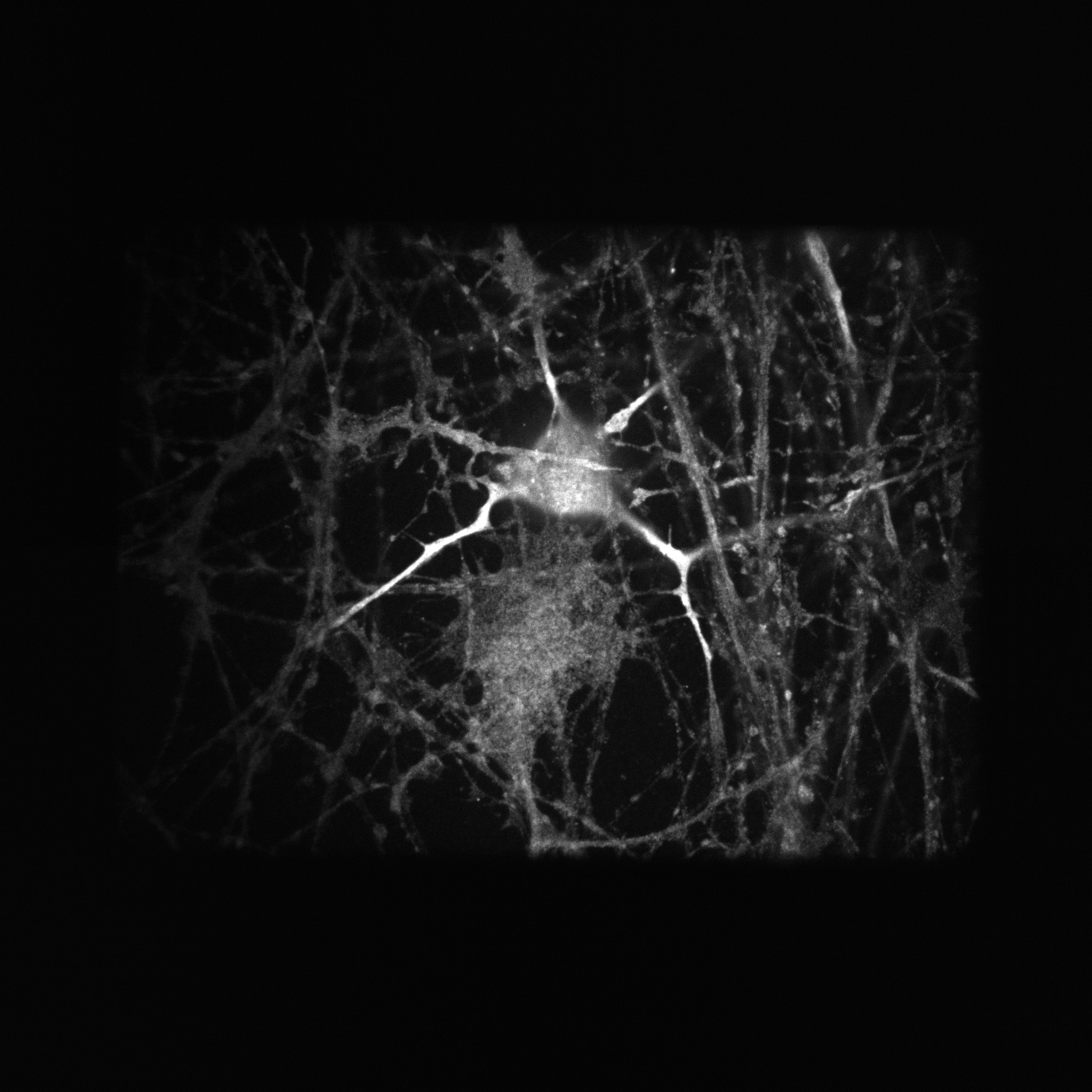

Supplement: Supplementary file 9 — Source data Fig. 5 [file 44318_2025_436_MOESM9_ESM.zip › Figure 5/5B/sgAKAP11 1/A11 1 tuj1map22_20240620_110012 AM/A11 1 tuj1map22_w0000_z0000.tif]

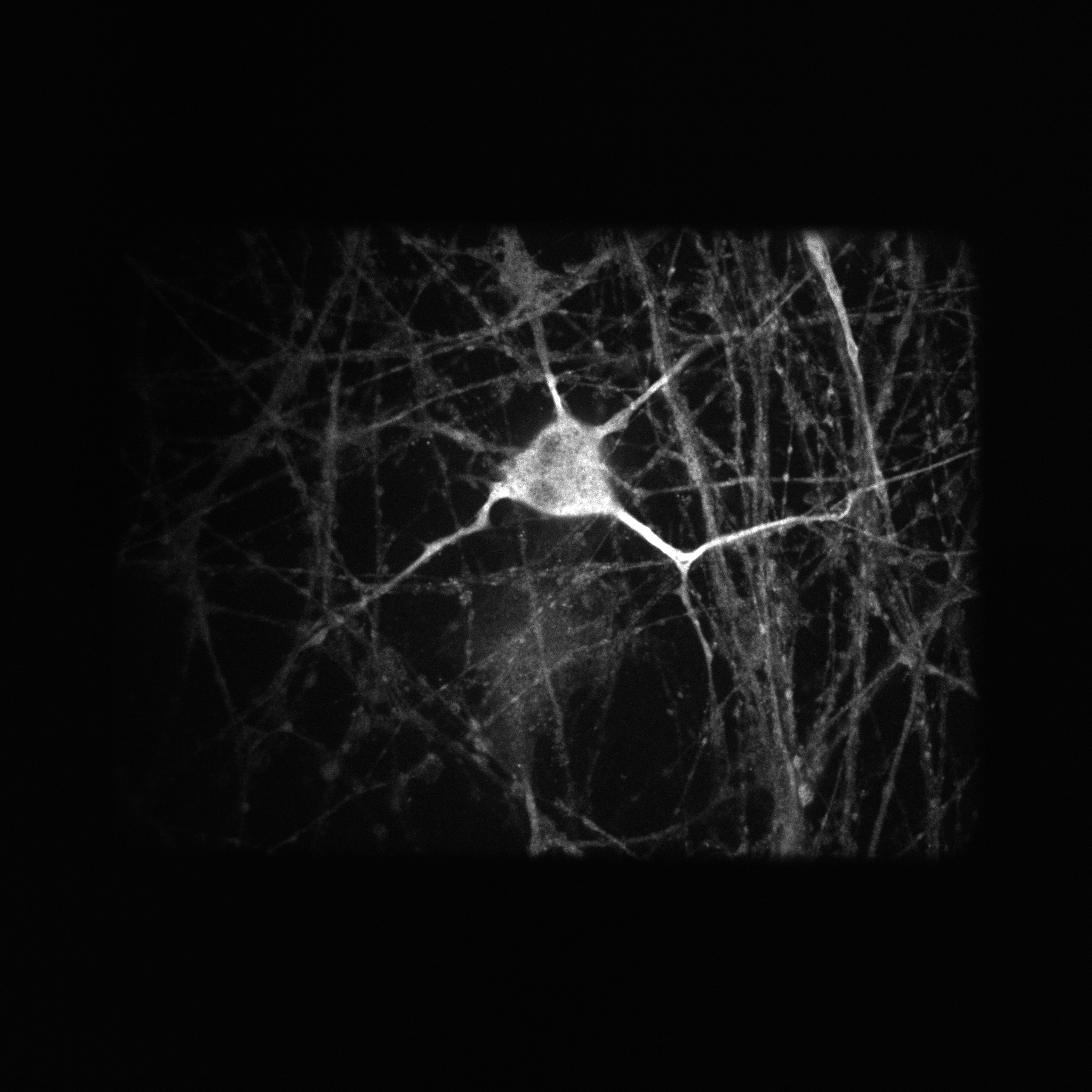

Supplement: Supplementary file 9 — Source data Fig. 5 [file 44318_2025_436_MOESM9_ESM.zip › Figure 5/5B/sgAKAP11 1/A11 1 tuj1map22_20240620_110012 AM/A11 1 tuj1map22_w0000_z0001.tif]

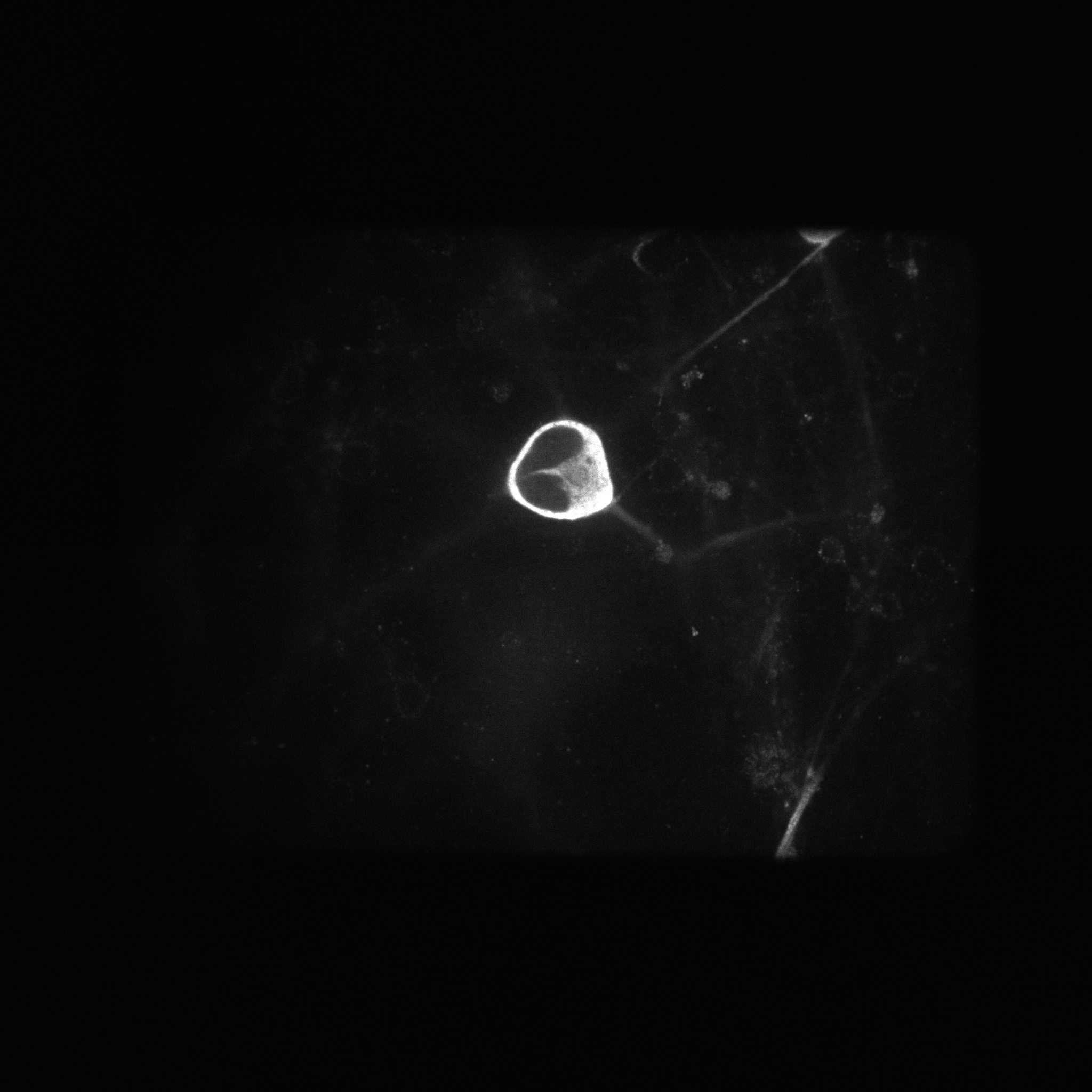

Supplement: Supplementary file 9 — Source data Fig. 5 [file 44318_2025_436_MOESM9_ESM.zip › Figure 5/5B/sgAKAP11 1/A11 1 tuj1map22_20240620_110012 AM/A11 1 tuj1map22_w0000_z0005.tif]

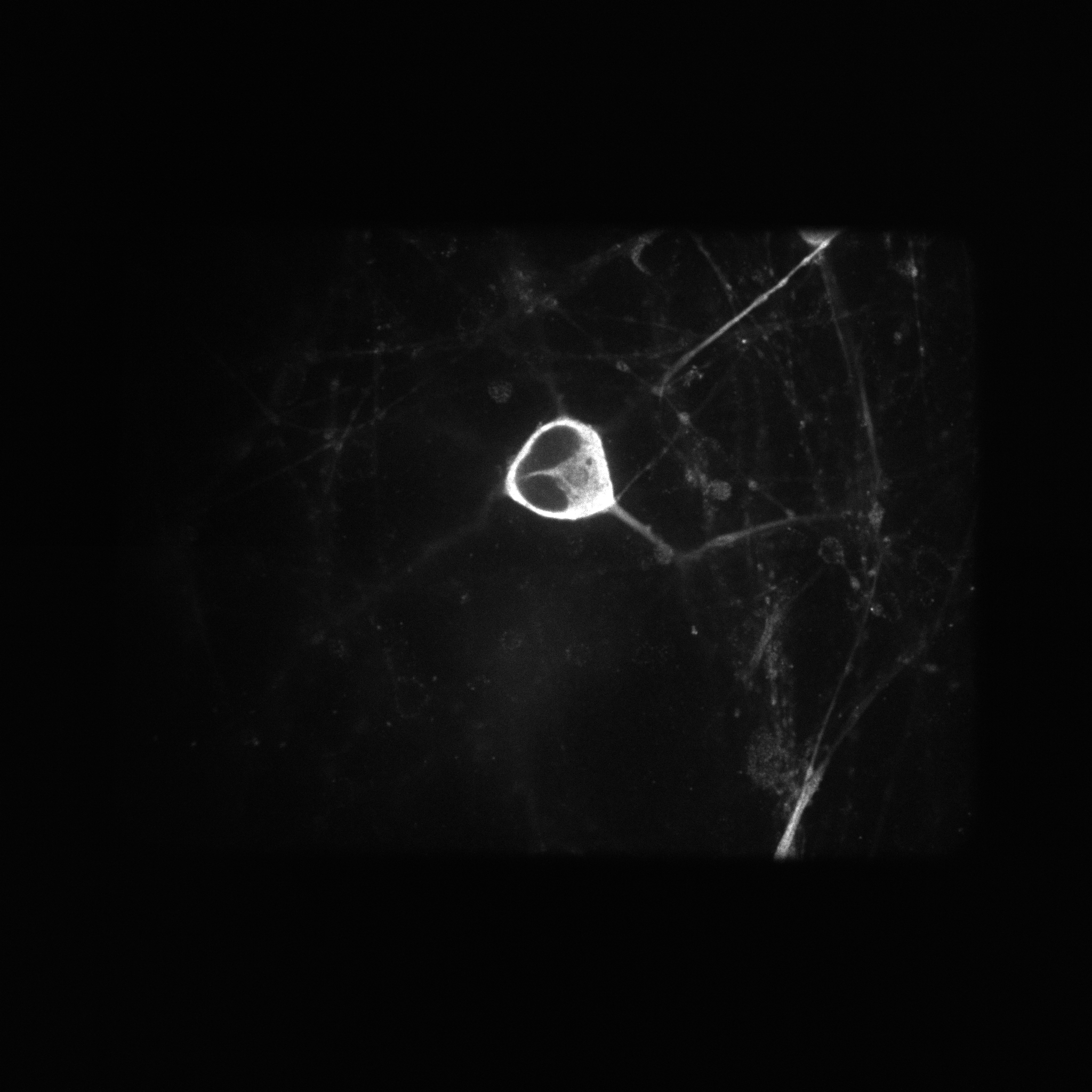

Supplement: Supplementary file 9 — Source data Fig. 5 [file 44318_2025_436_MOESM9_ESM.zip › Figure 5/5B/sgAKAP11 1/A11 1 tuj1map22_20240620_110012 AM/A11 1 tuj1map22_w0000_z0004.tif]

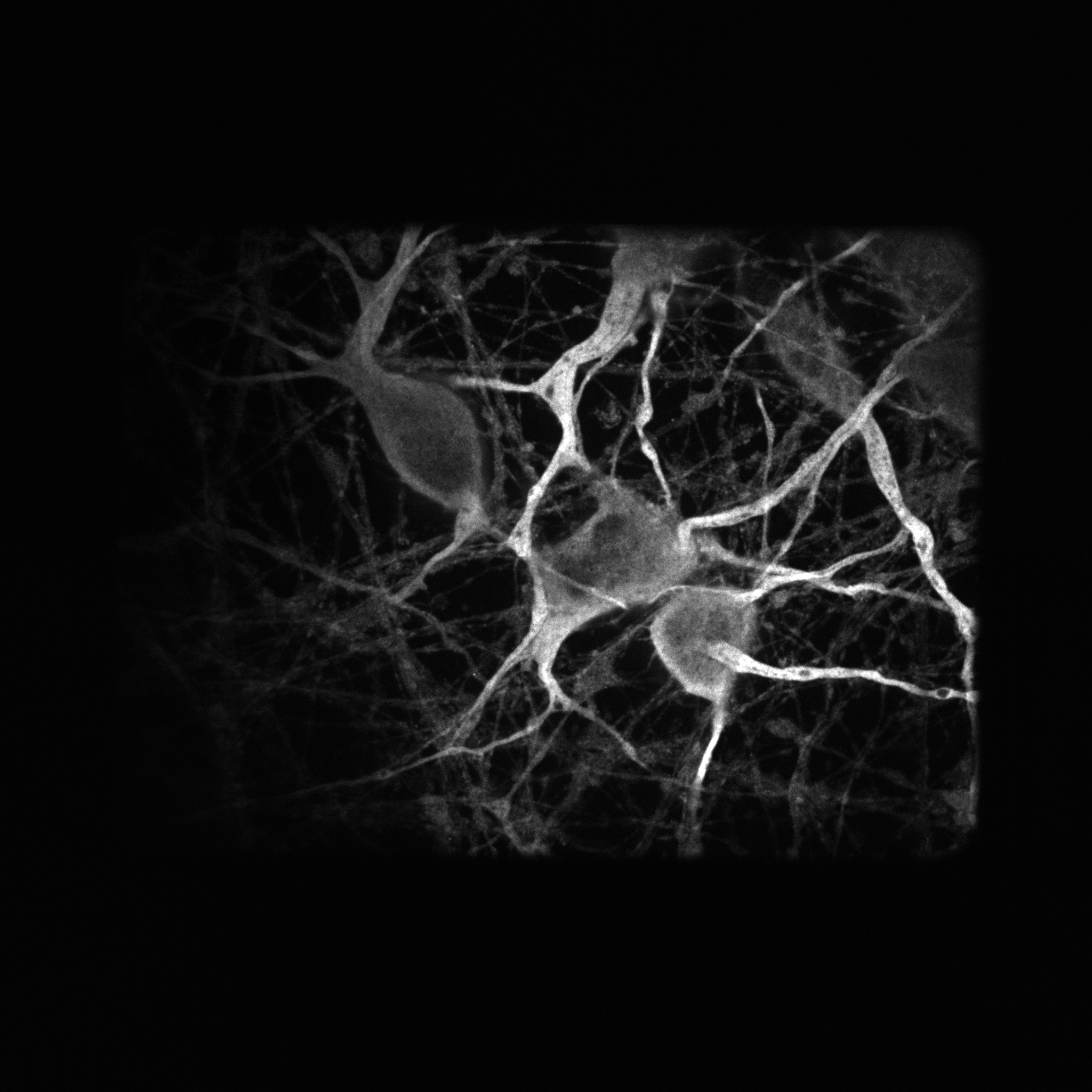

Supplement: Supplementary file 9 — Source data Fig. 5 [file 44318_2025_436_MOESM9_ESM.zip › Figure 5/5B/sgNT/sgnt 1 tuj1map22_20240620_111922 AM copy/sgnt 1 tuj1map22_w0000_z0000.tif]

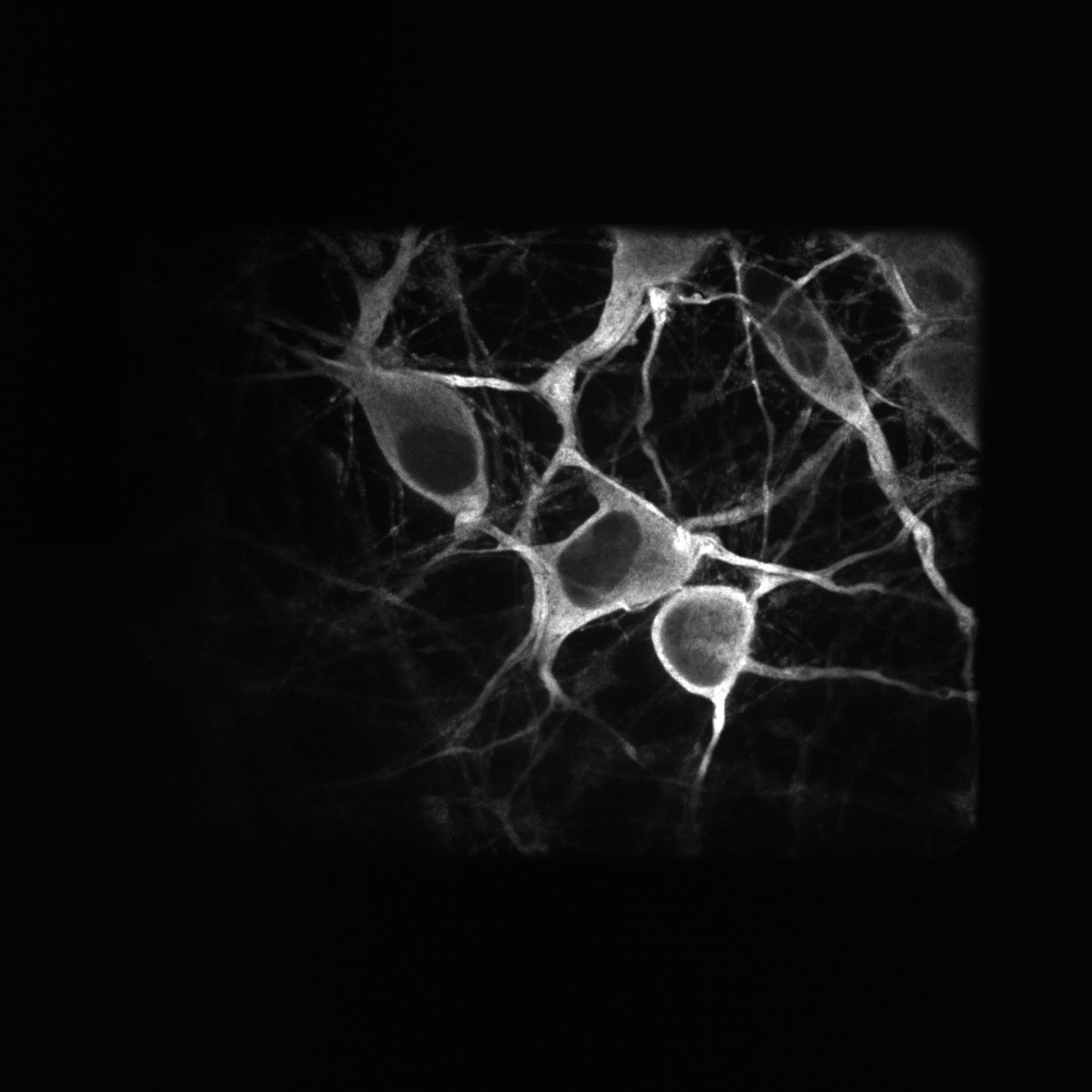

Supplement: Supplementary file 9 — Source data Fig. 5 [file 44318_2025_436_MOESM9_ESM.zip › Figure 5/5B/sgNT/sgnt 1 tuj1map22_20240620_111922 AM copy/sgnt 1 tuj1map22_w0000_z0001.tif]

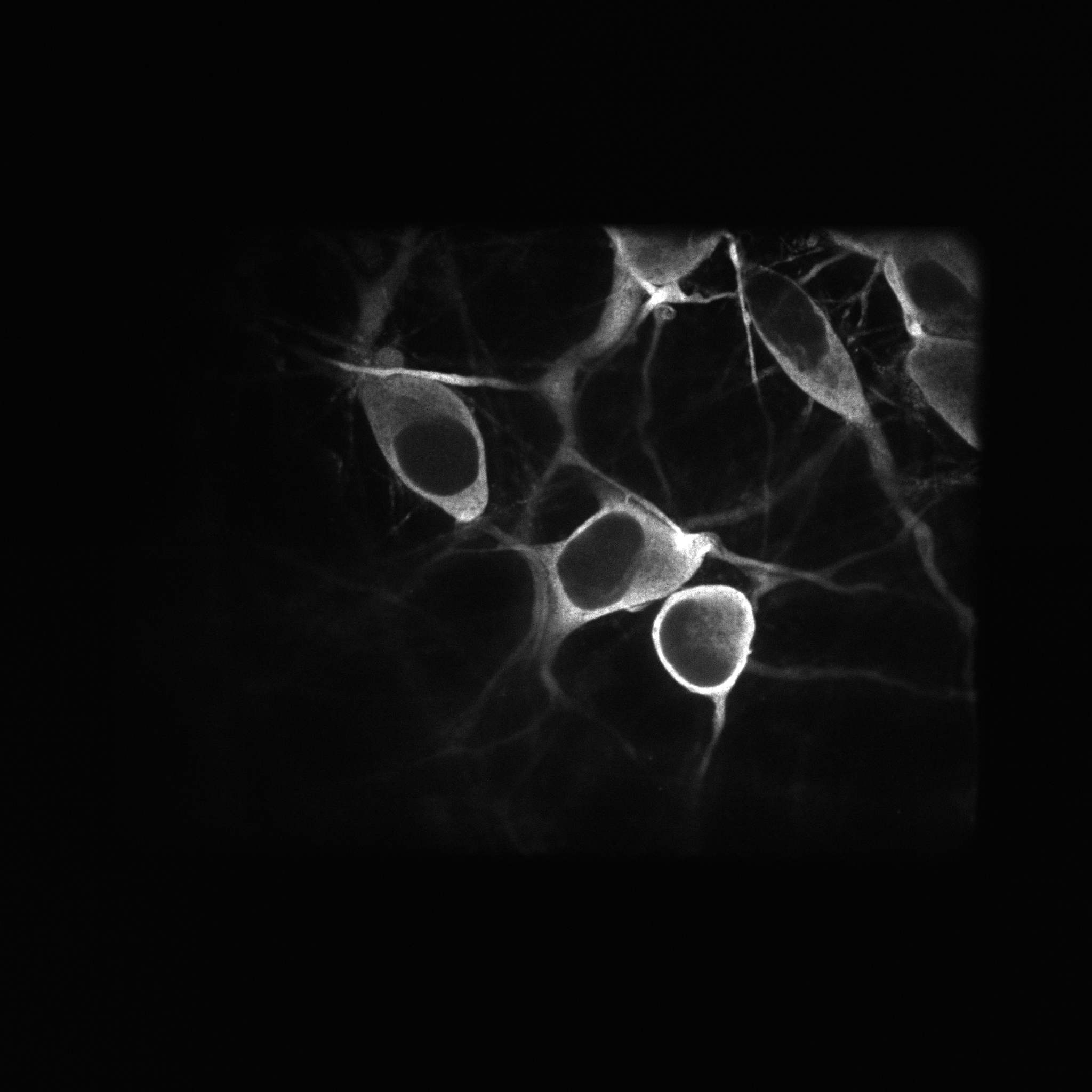

Supplement: Supplementary file 9 — Source data Fig. 5 [file 44318_2025_436_MOESM9_ESM.zip › Figure 5/5B/sgNT/sgnt 1 tuj1map22_20240620_111922 AM copy/sgnt 1 tuj1map22_w0000_z0002.tif]

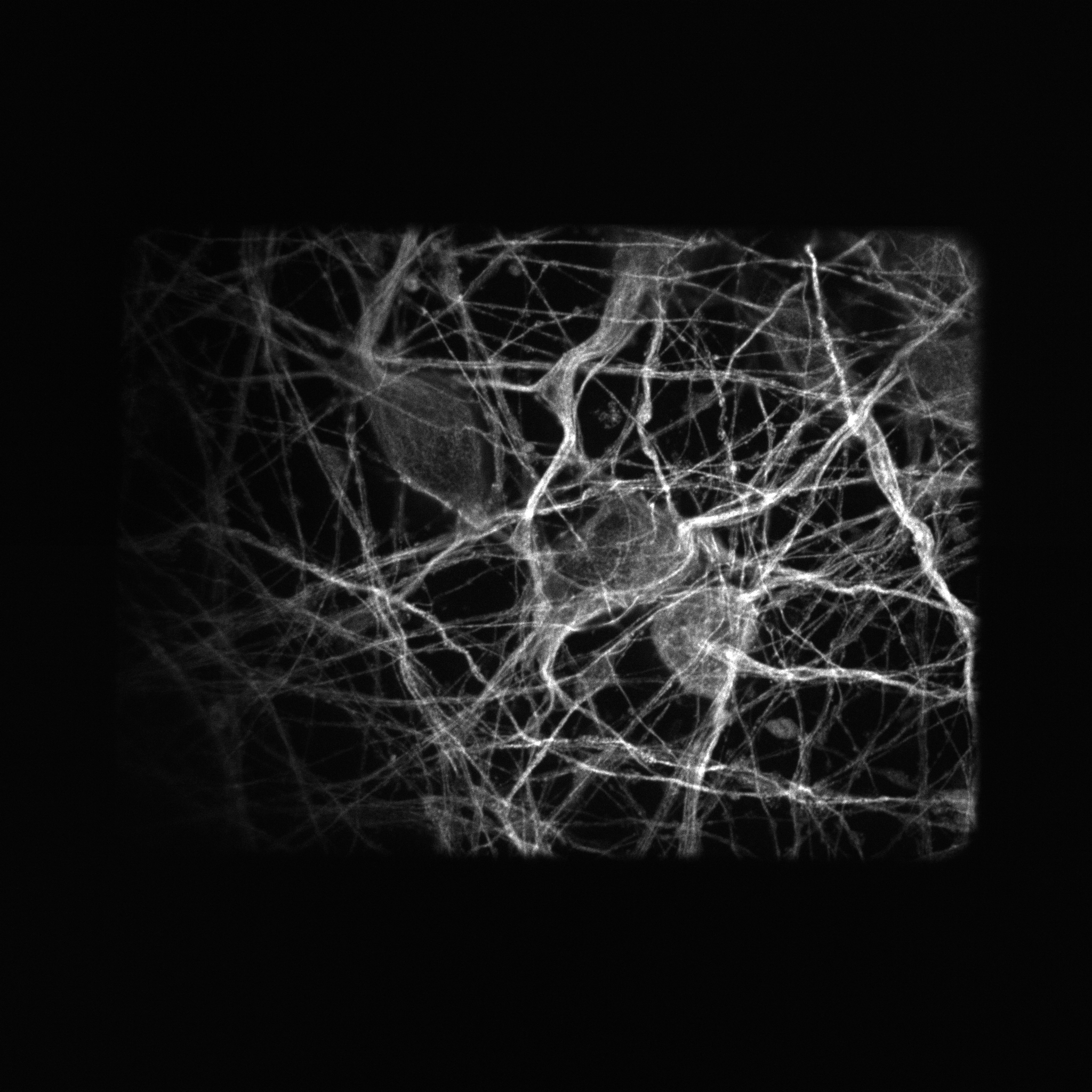

Supplement: Supplementary file 9 — Source data Fig. 5 [file 44318_2025_436_MOESM9_ESM.zip › Figure 5/5B/sgNT/sgnt 1 tuj1map22_20240620_111922 AM copy/sgnt 1 tuj1map22_w0001_z0000.tif]

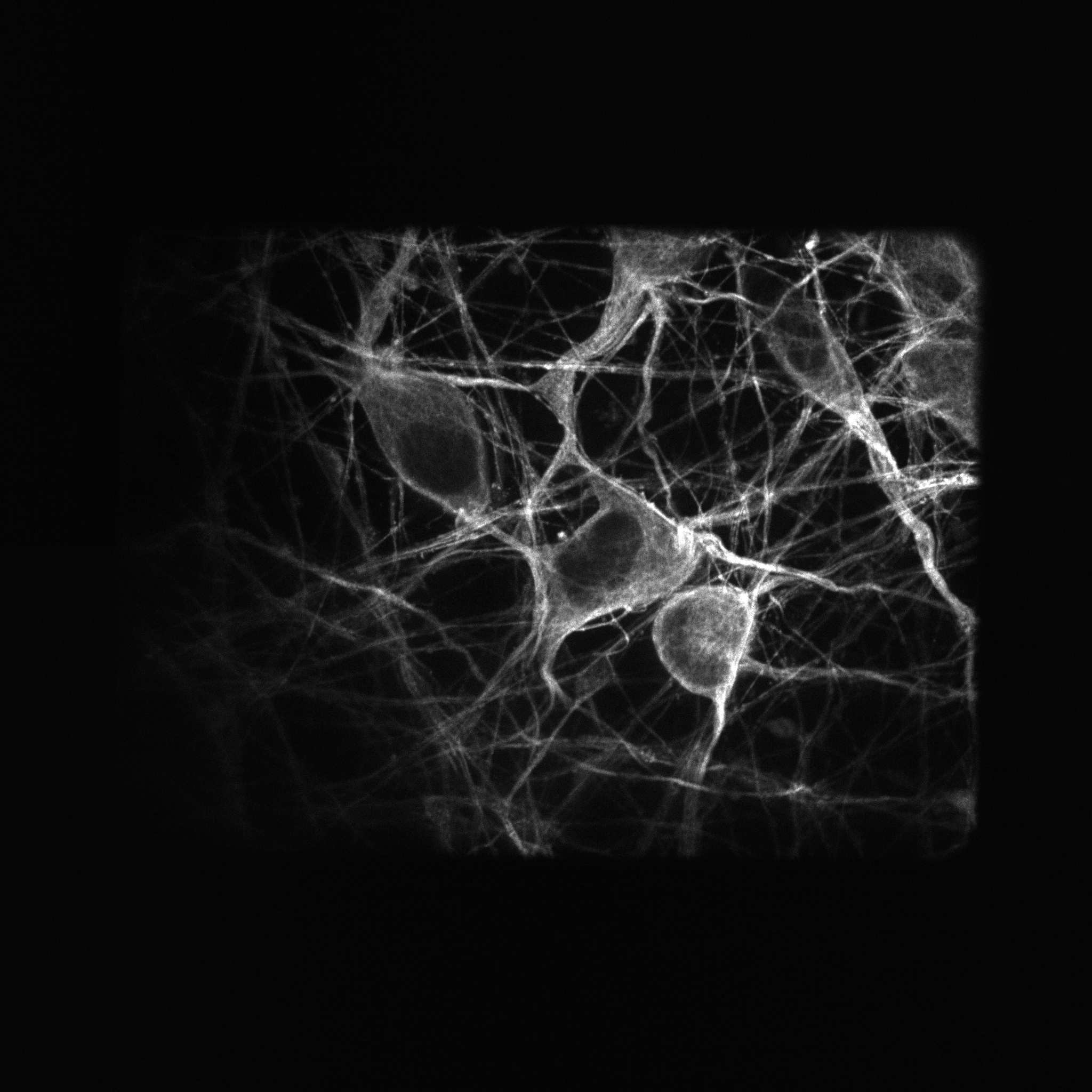

Supplement: Supplementary file 9 — Source data Fig. 5 [file 44318_2025_436_MOESM9_ESM.zip › Figure 5/5B/sgNT/sgnt 1 tuj1map22_20240620_111922 AM copy/sgnt 1 tuj1map22_w0001_z0001.tif]

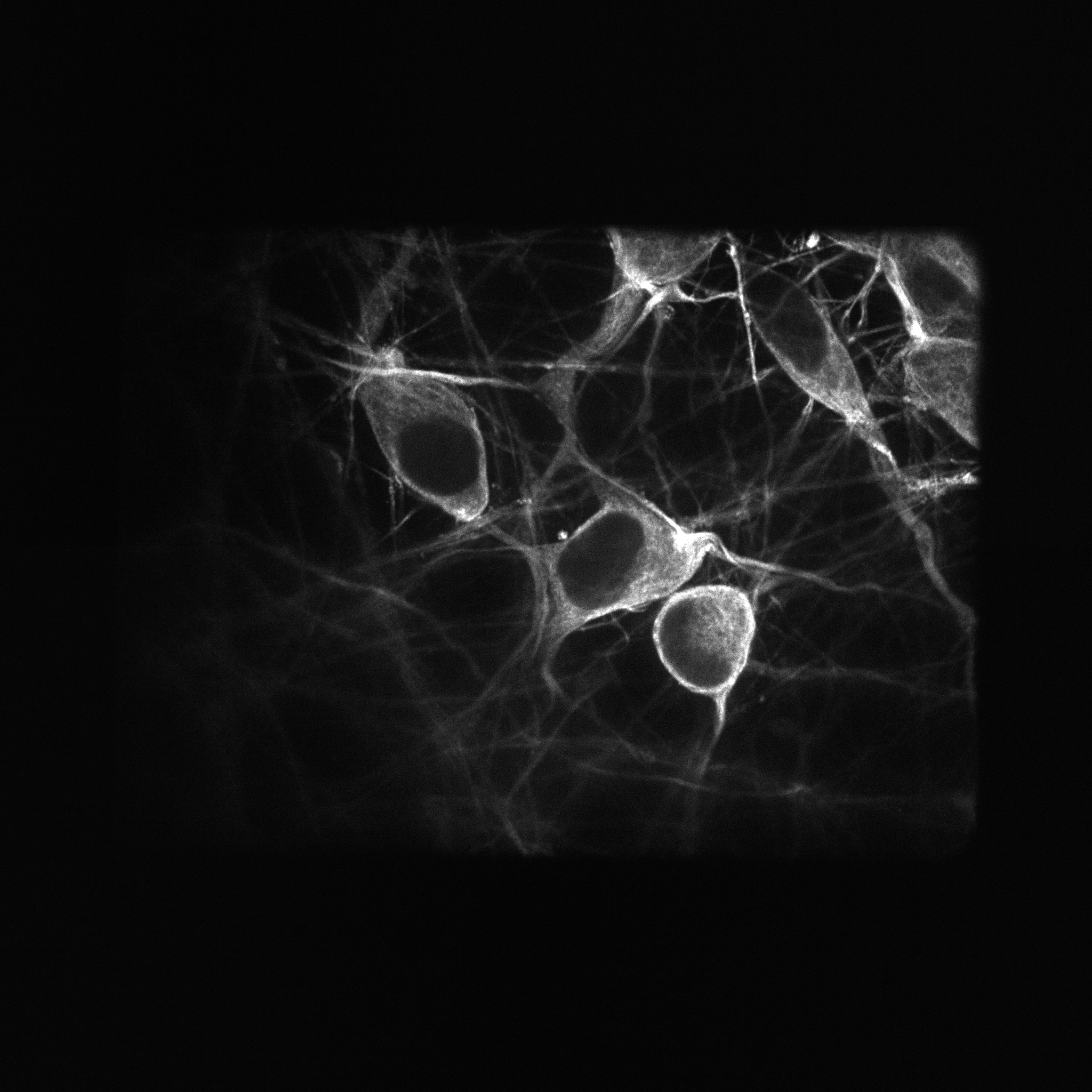

Supplement: Supplementary file 9 — Source data Fig. 5 [file 44318_2025_436_MOESM9_ESM.zip › Figure 5/5B/sgNT/sgnt 1 tuj1map22_20240620_111922 AM copy/sgnt 1 tuj1map22_w0001_z0002.tif]

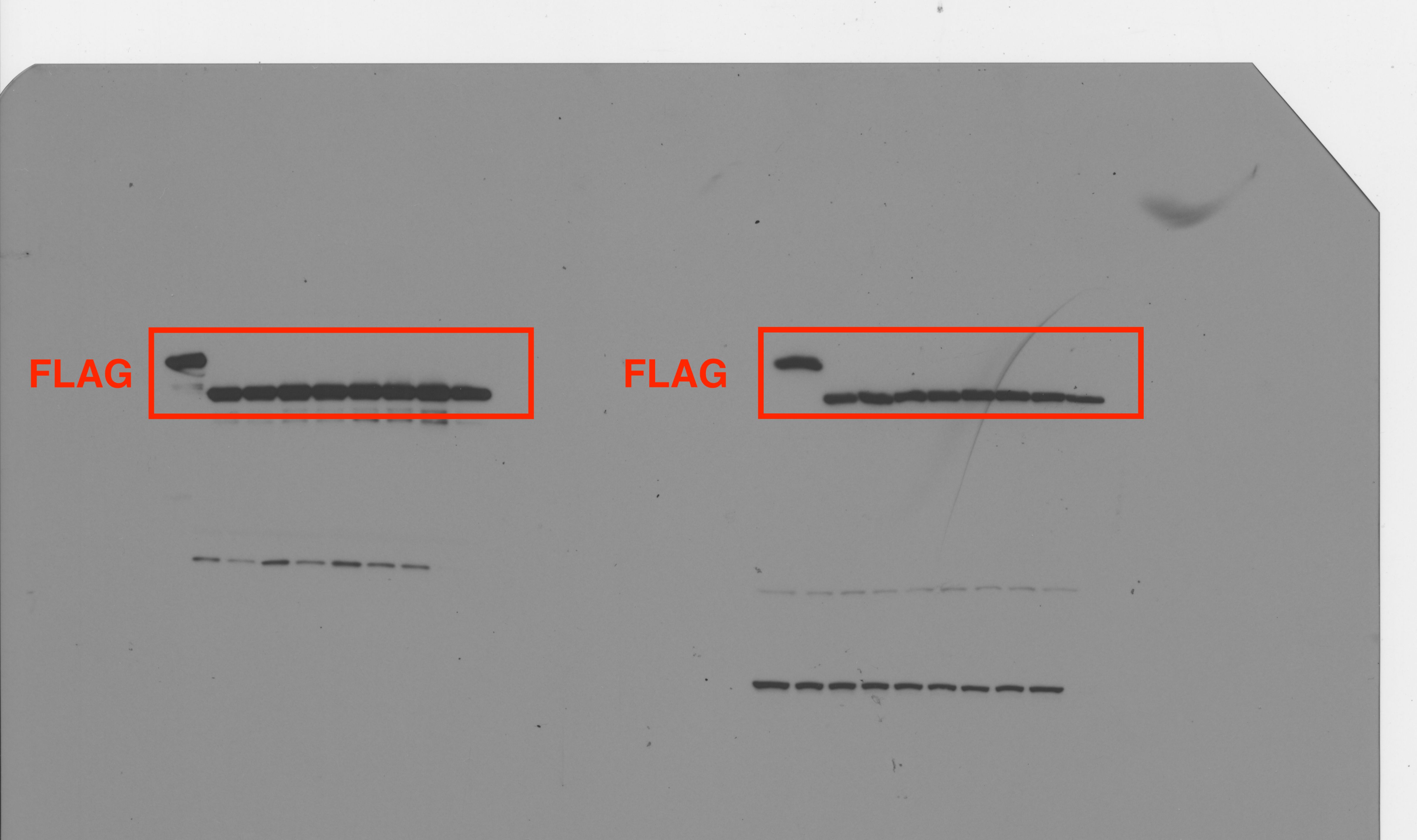

Supplement: Supplementary file 10 — Source data Fig. 6 [file 44318_2025_436_MOESM10_ESM.zip › Figure 6/6F/20240726_0002.tif]

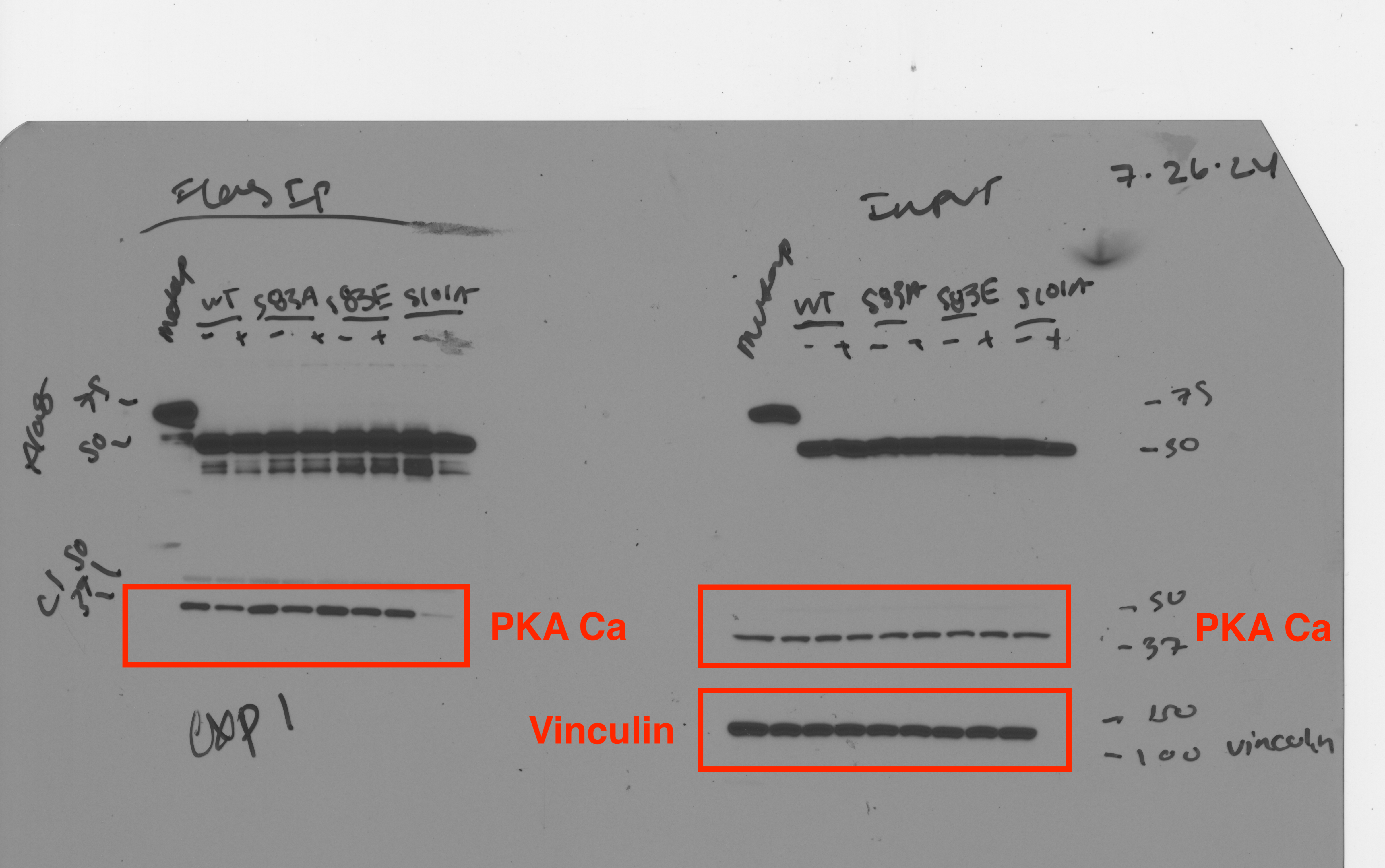

Supplement: Supplementary file 10 — Source data Fig. 6 [file 44318_2025_436_MOESM10_ESM.zip › Figure 6/6F/20240726_0001.tif]

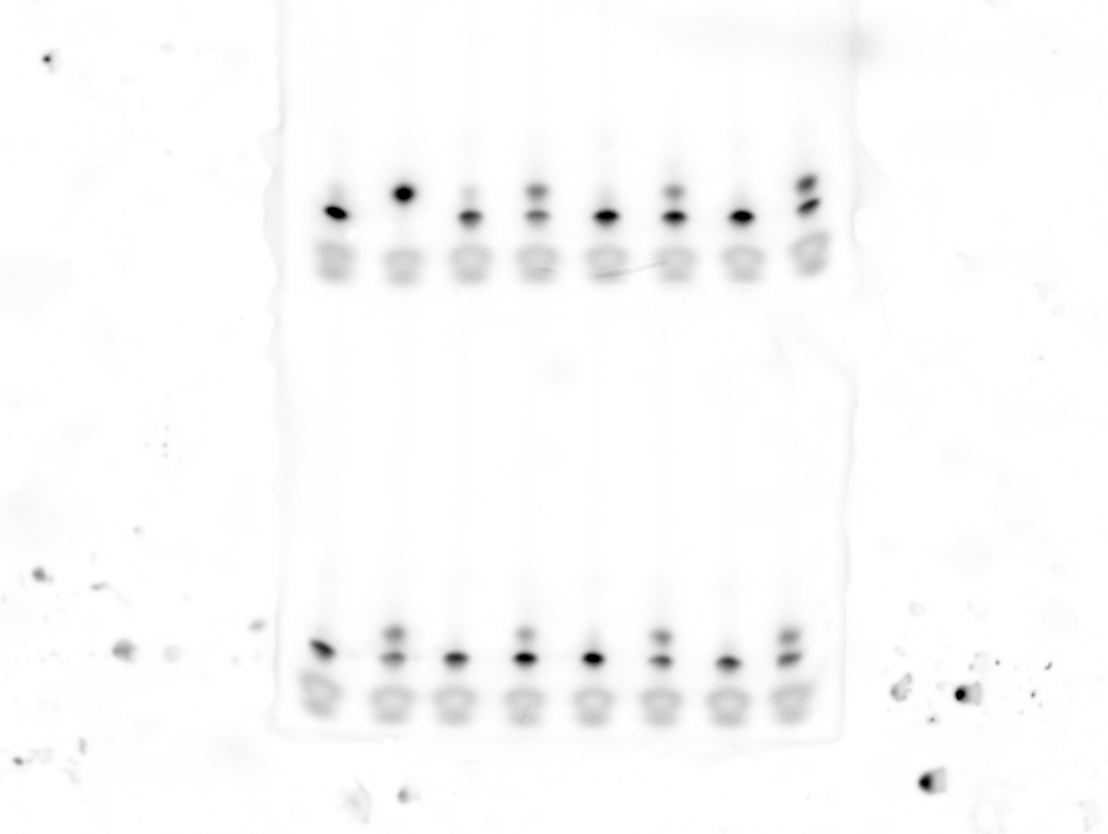

Supplement: Supplementary file 10 — Source data Fig. 6 [file 44318_2025_436_MOESM10_ESM.zip › Figure 6/6D/Triplicate#3.png]

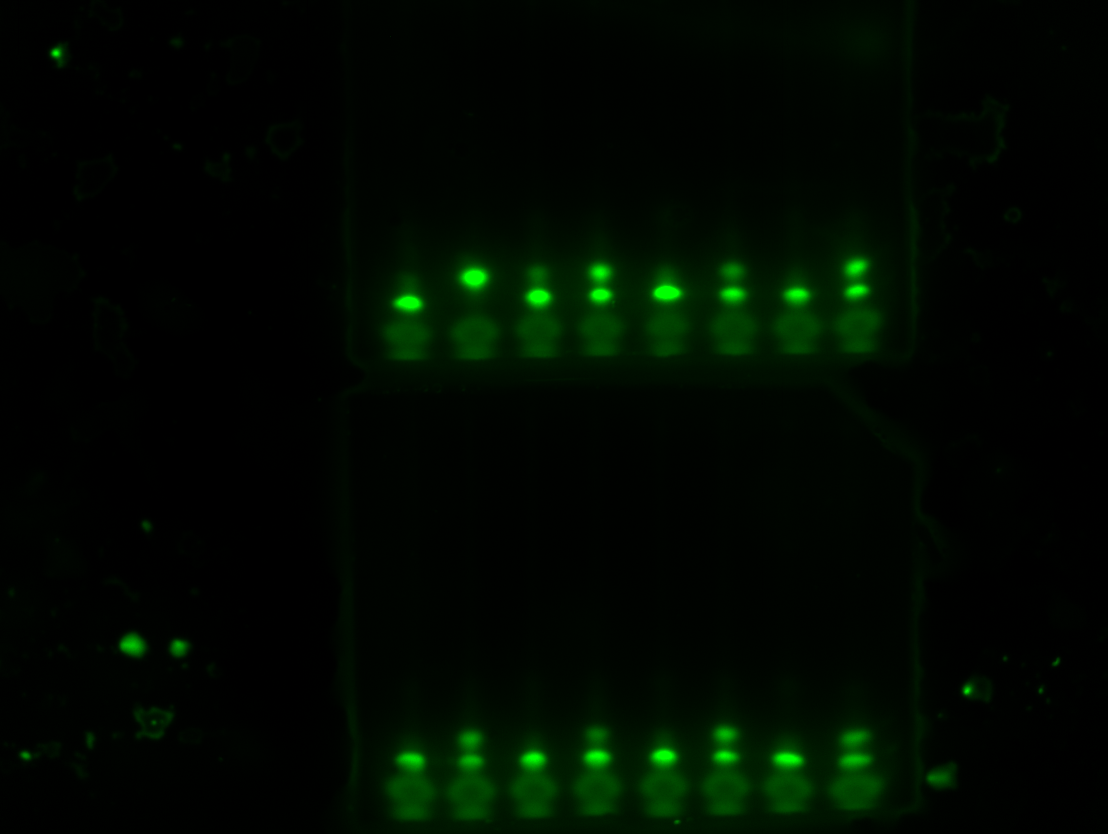

Supplement: Supplementary file 10 — Source data Fig. 6 [file 44318_2025_436_MOESM10_ESM.zip › Figure 6/6D/2024-07-12_Replicate1_cAMPSeperated/2024-07-12_17-58-45_8bit.png]

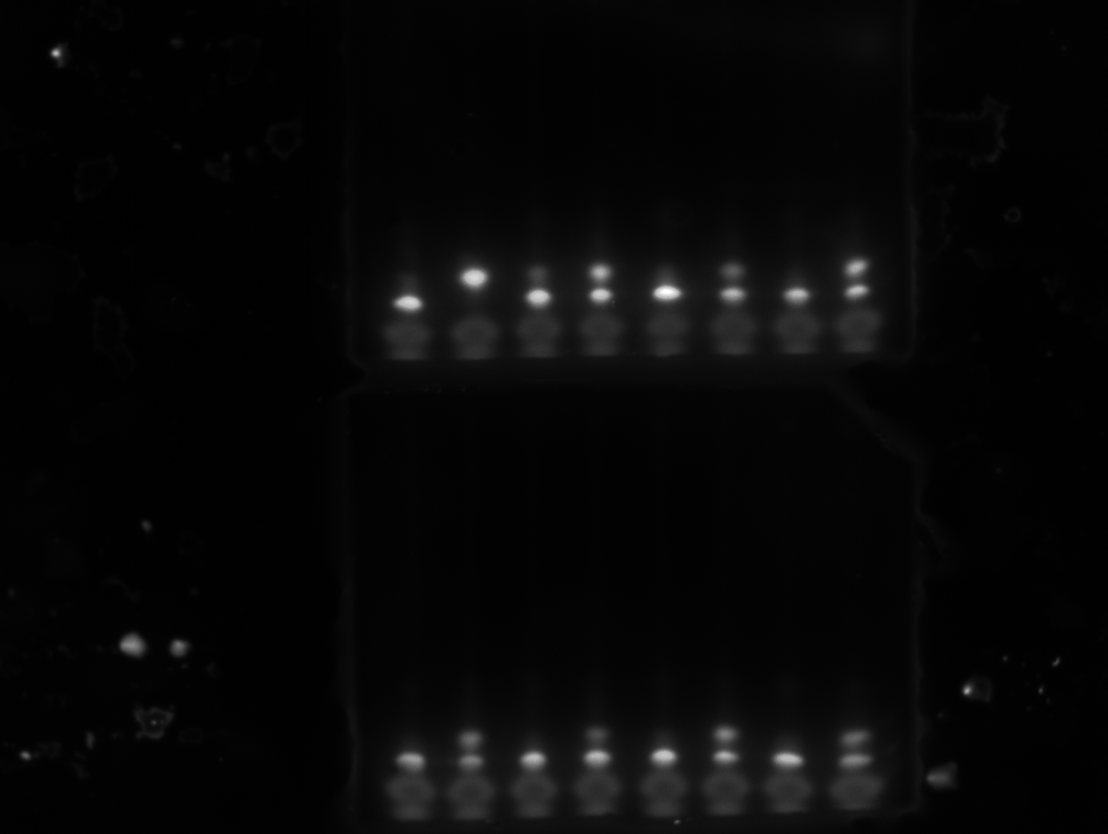

Supplement: Supplementary file 10 — Source data Fig. 6 [file 44318_2025_436_MOESM10_ESM.zip › Figure 6/6D/2024-07-12_Replicate1_cAMPSeperated/2024-07-12_17-58-45_1_16bit.png]

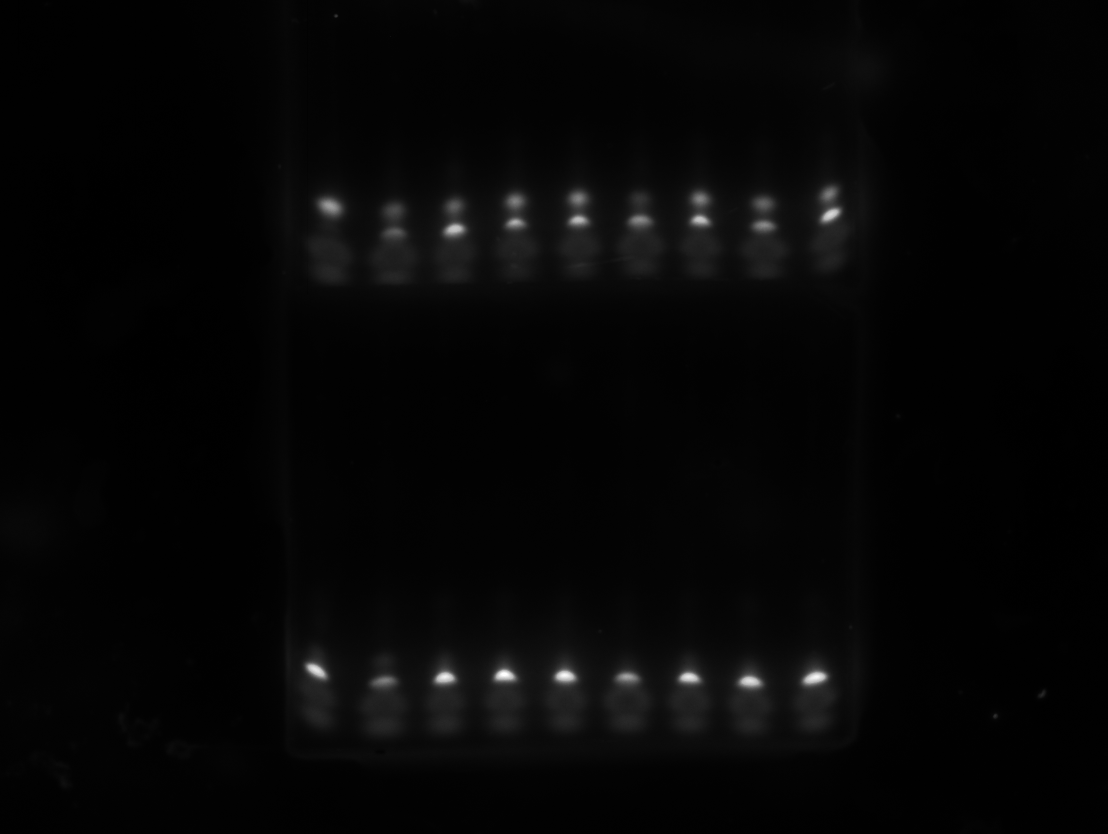

Supplement: Supplementary file 10 — Source data Fig. 6 [file 44318_2025_436_MOESM10_ESM.zip › Figure 6/6D/2024-07-12_Replicate3/2024-07-12_16-33-31_1_16bit.png]

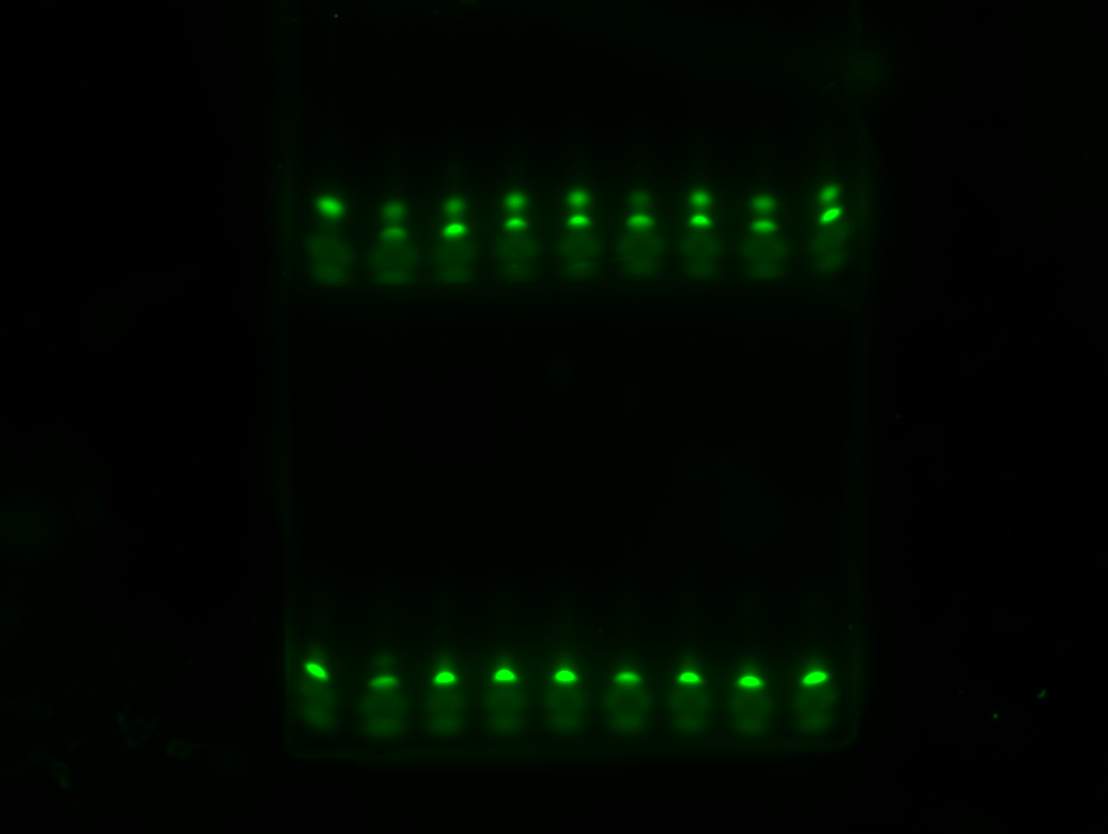

Supplement: Supplementary file 10 — Source data Fig. 6 [file 44318_2025_436_MOESM10_ESM.zip › Figure 6/6D/2024-07-12_Replicate3/2024-07-12_16-33-31_8bit.png]

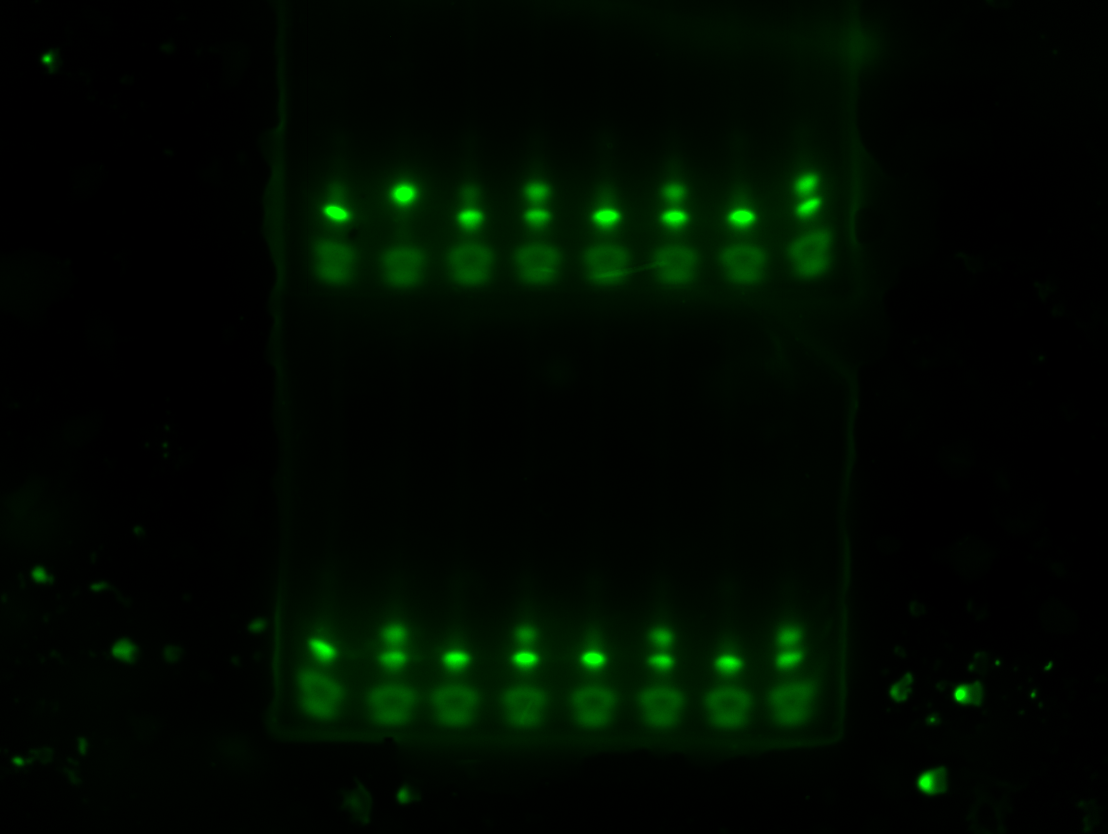

Supplement: Supplementary file 10 — Source data Fig. 6 [file 44318_2025_436_MOESM10_ESM.zip › Figure 6/6D/2024-07-12_Replicate2/2024-07-12_17-29-17_8bit.png]

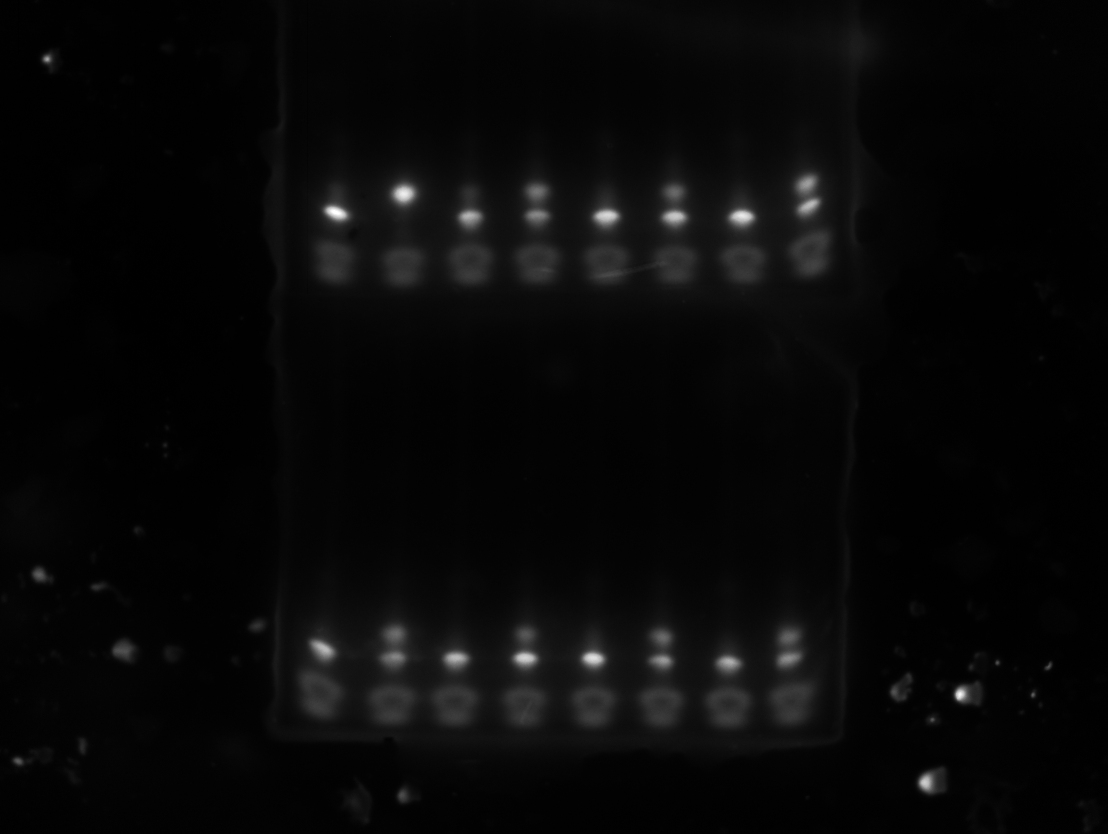

Supplement: Supplementary file 10 — Source data Fig. 6 [file 44318_2025_436_MOESM10_ESM.zip › Figure 6/6D/2024-07-12_Replicate2/2024-07-12_17-29-17_1_16bit.png]

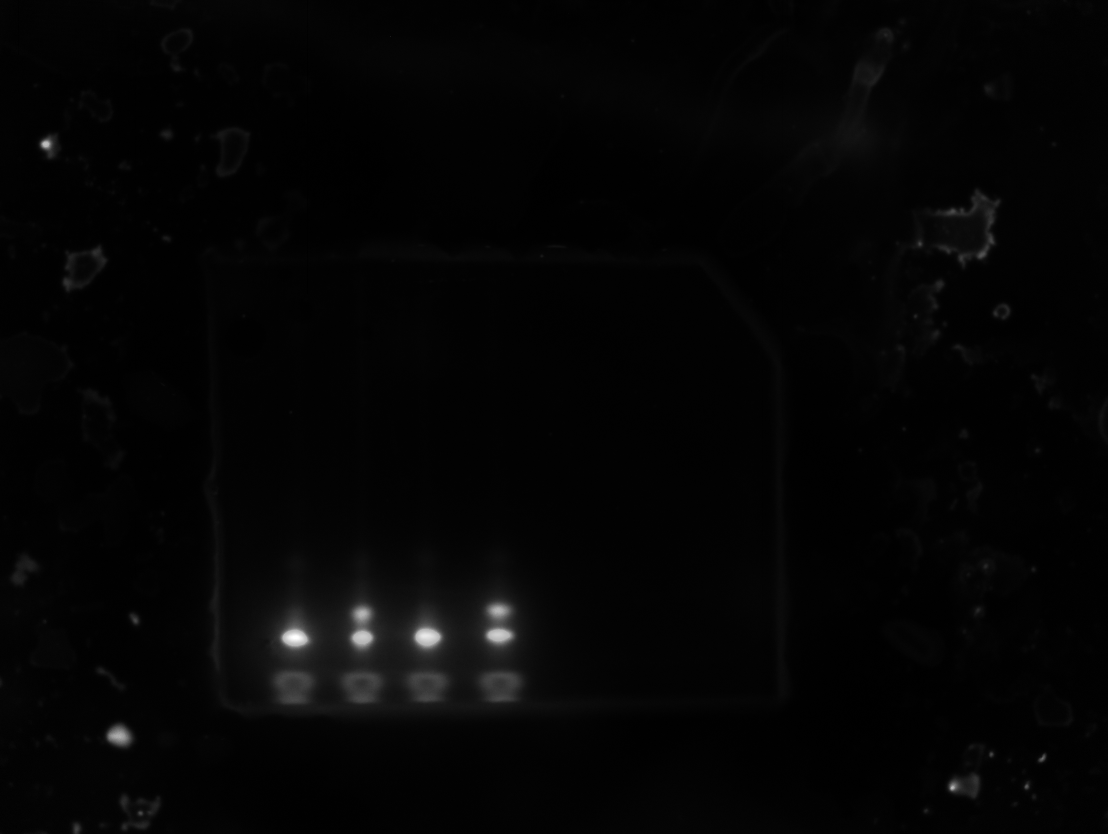

Supplement: Supplementary file 10 — Source data Fig. 6 [file 44318_2025_436_MOESM10_ESM.zip › Figure 6/6D/2024-07-12_Replicate2_3T338D/2024-07-12_18-11-13_1_16bit.png]

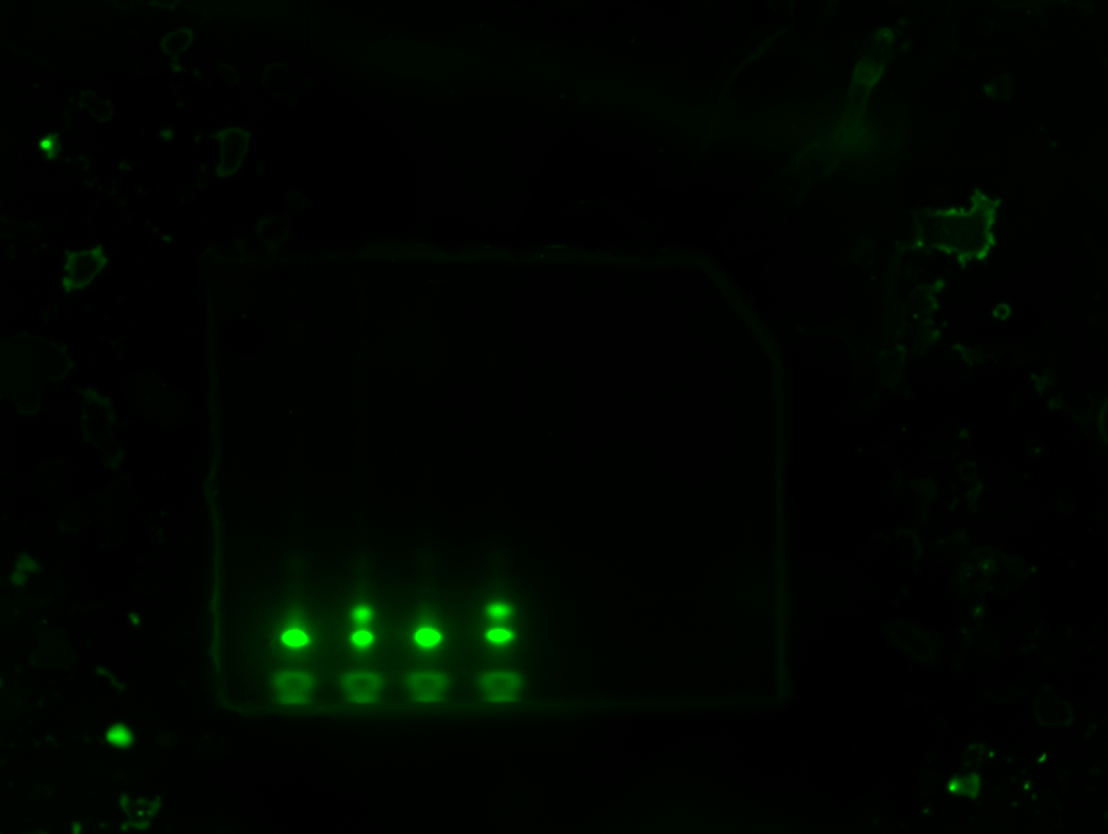

Supplement: Supplementary file 10 — Source data Fig. 6 [file 44318_2025_436_MOESM10_ESM.zip › Figure 6/6D/2024-07-12_Replicate2_3T338D/2024-07-12_18-11-13_8bit.png]

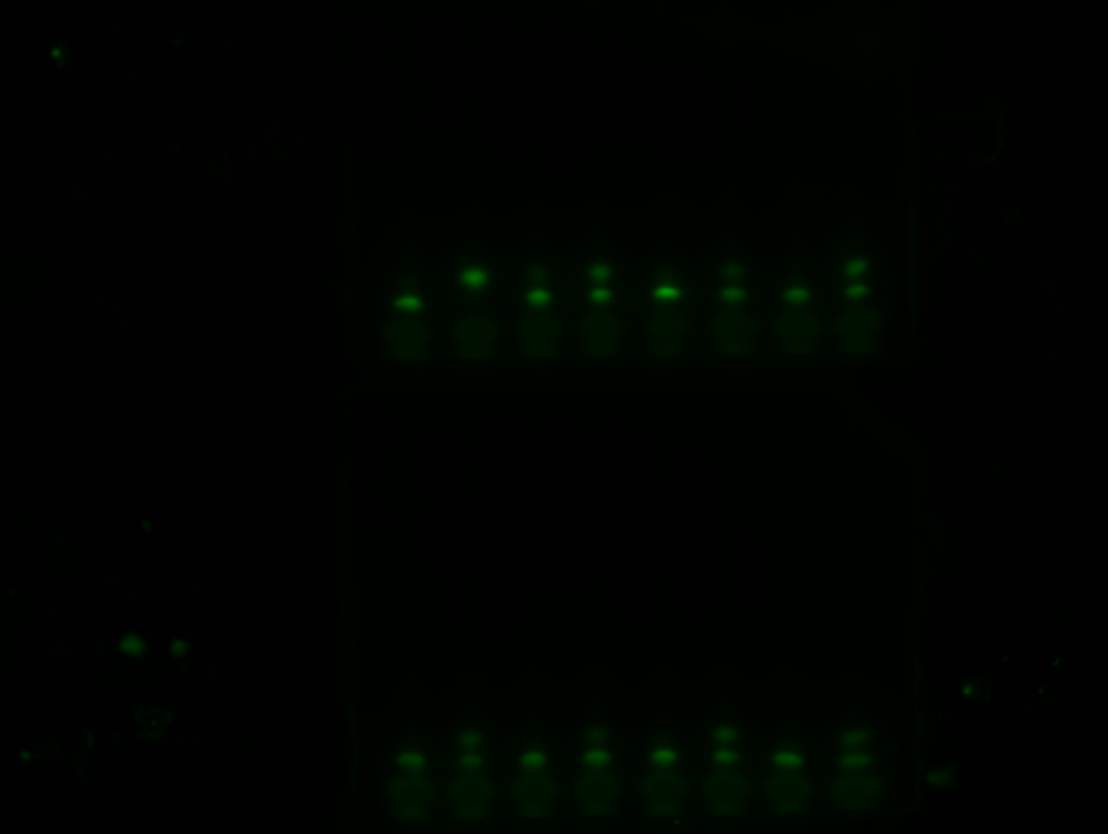

Supplement: Supplementary file 10 — Source data Fig. 6 [file 44318_2025_436_MOESM10_ESM.zip › Figure 6/6D/2024-07-12_Replicate1_cAMPSeperated/contrast/contrast_0.png]

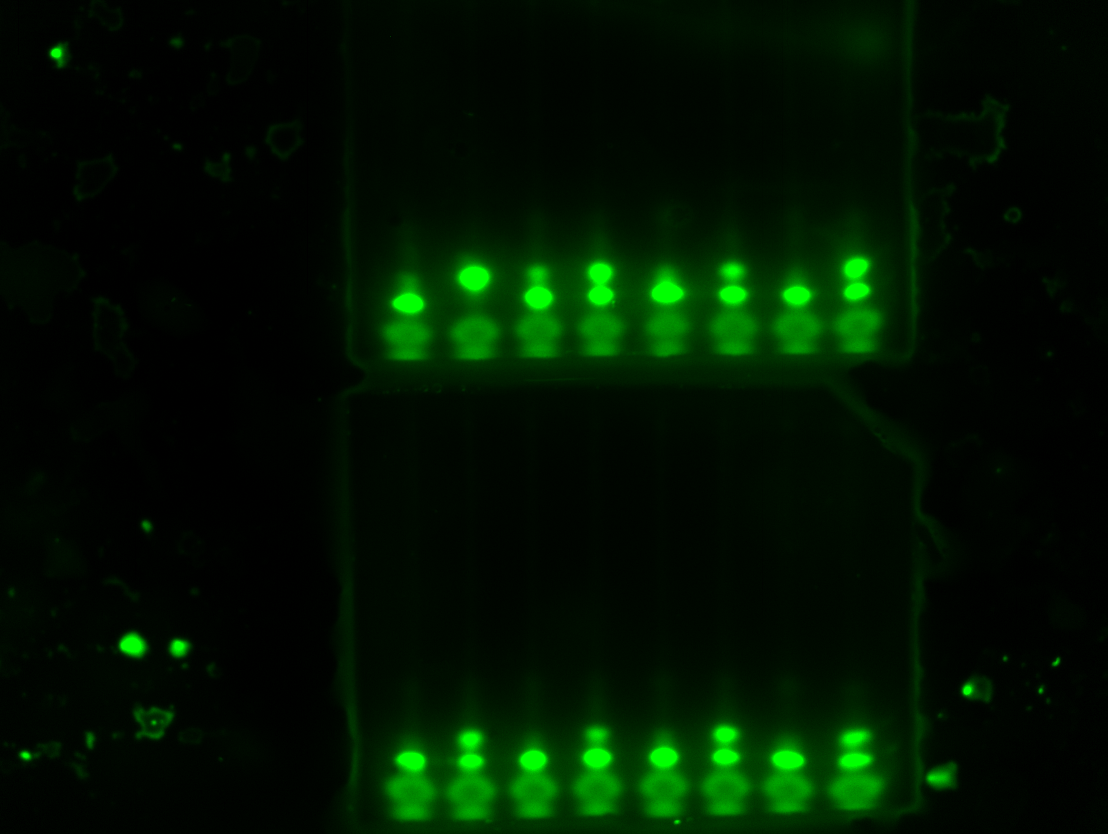

Supplement: Supplementary file 10 — Source data Fig. 6 [file 44318_2025_436_MOESM10_ESM.zip › Figure 6/6D/2024-07-12_Replicate1_cAMPSeperated/contrast/contrast_3.png]

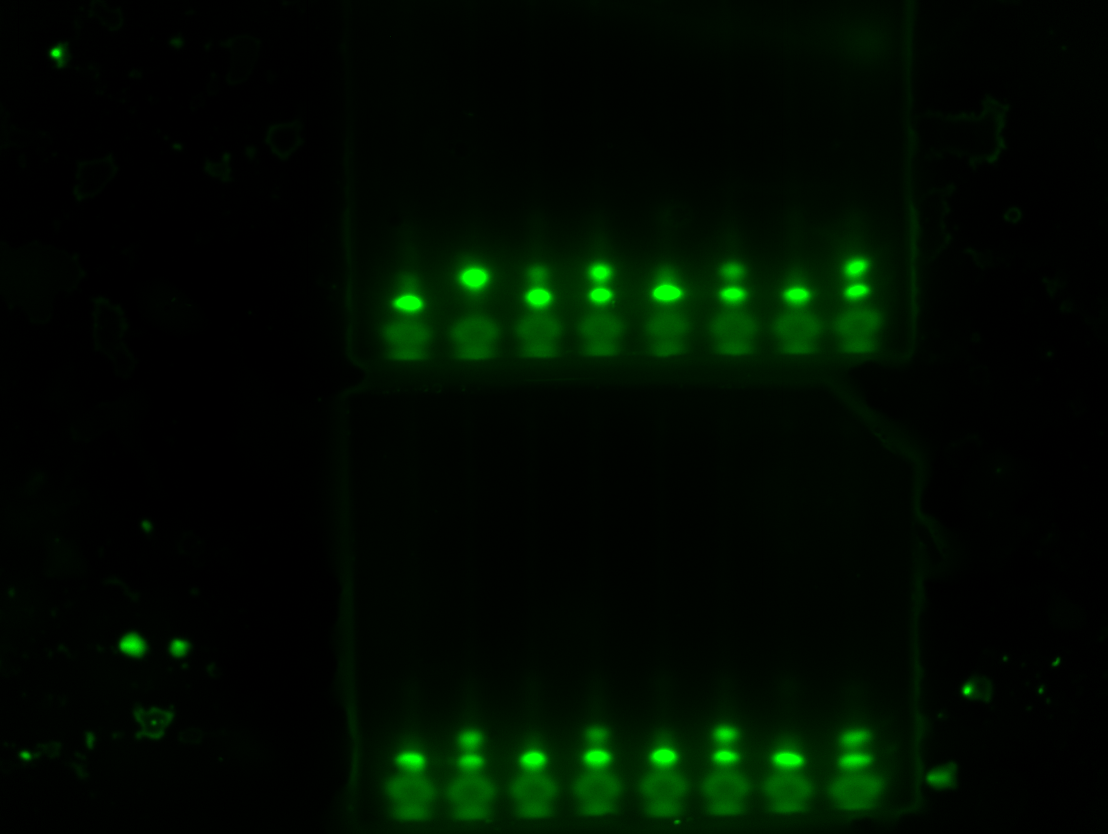

Supplement: Supplementary file 10 — Source data Fig. 6 [file 44318_2025_436_MOESM10_ESM.zip › Figure 6/6D/2024-07-12_Replicate1_cAMPSeperated/contrast/contrast_2.png]

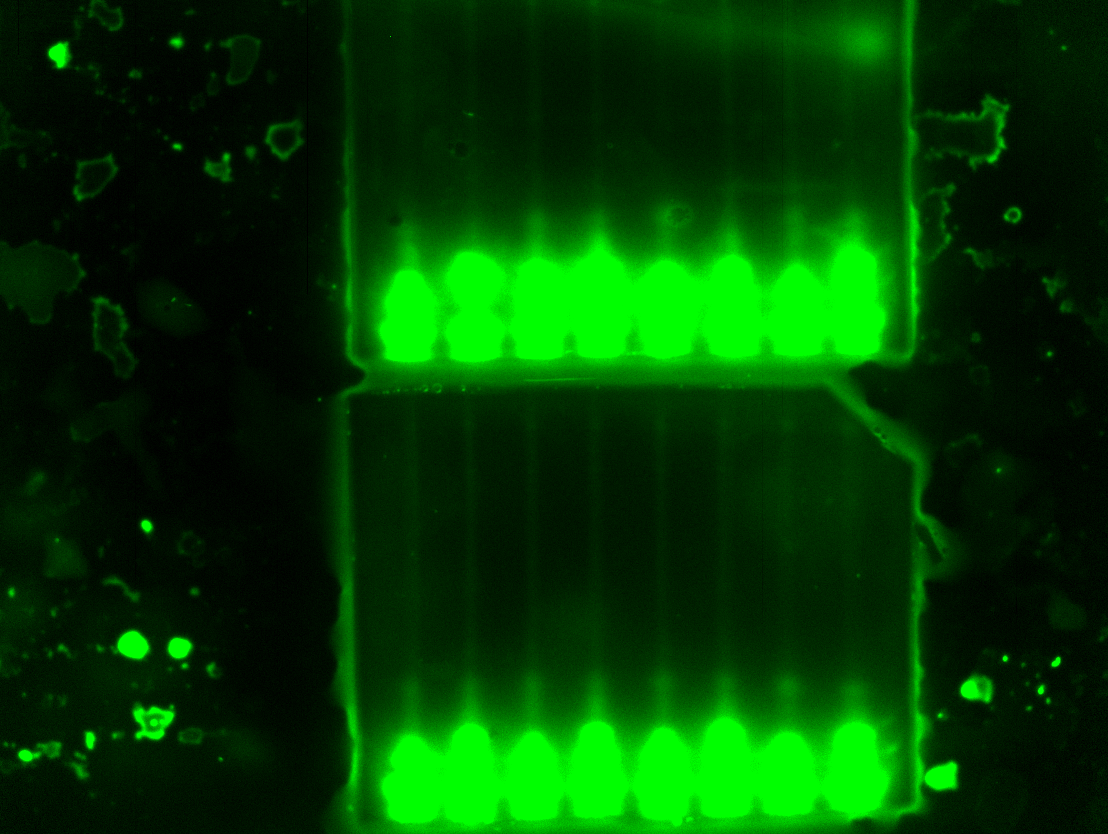

Supplement: Supplementary file 10 — Source data Fig. 6 [file 44318_2025_436_MOESM10_ESM.zip › Figure 6/6D/2024-07-12_Replicate1_cAMPSeperated/contrast/contrast_6.png]

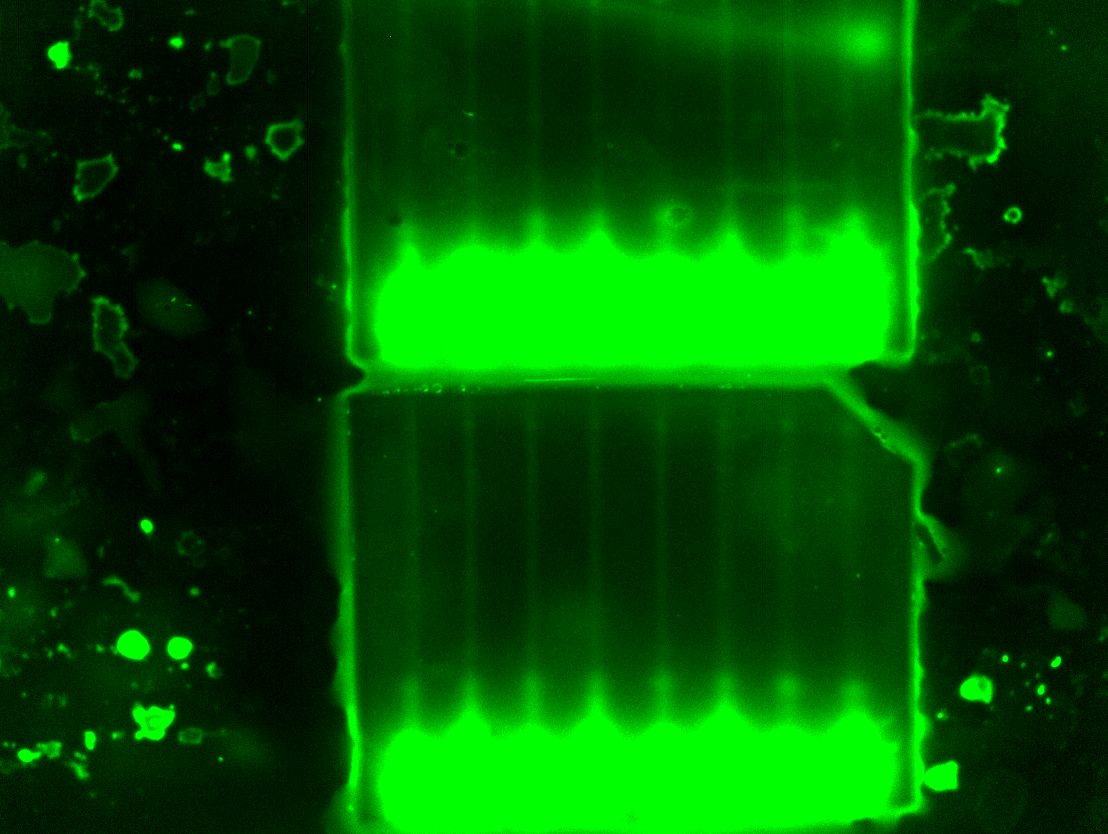

Supplement: Supplementary file 10 — Source data Fig. 6 [file 44318_2025_436_MOESM10_ESM.zip › Figure 6/6D/2024-07-12_Replicate1_cAMPSeperated/contrast/contrast_7.png]

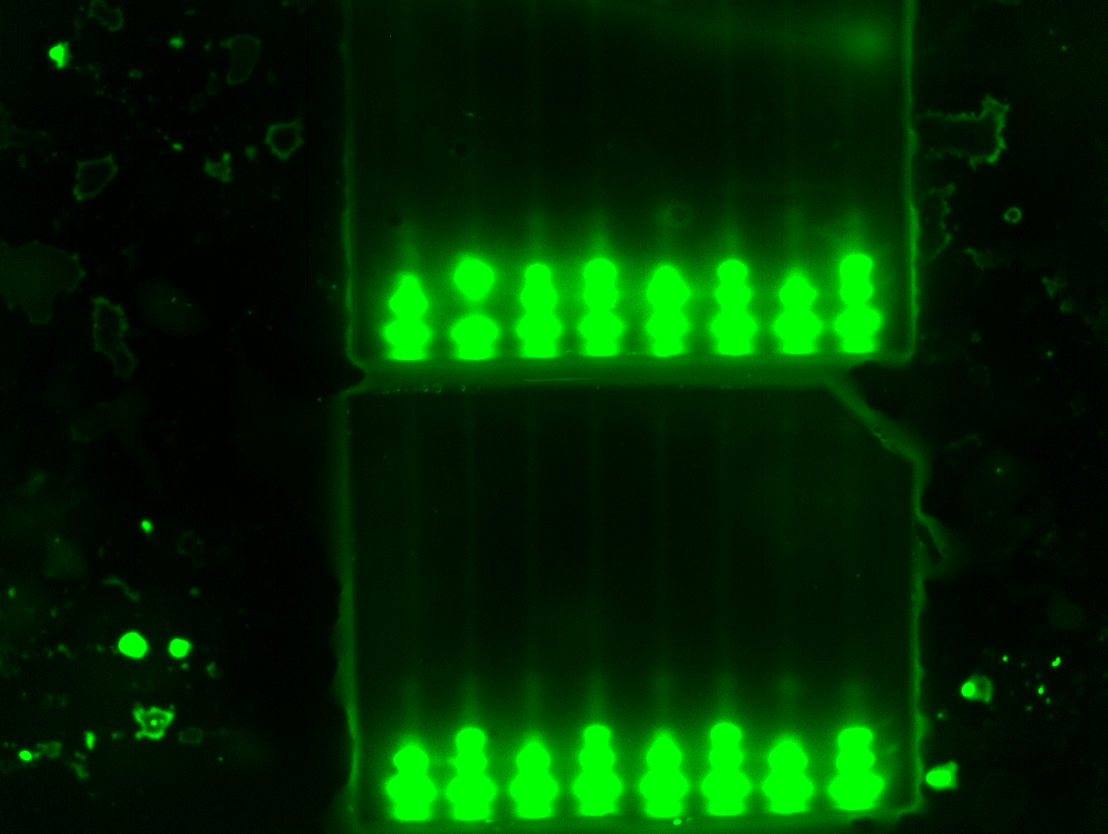

Supplement: Supplementary file 10 — Source data Fig. 6 [file 44318_2025_436_MOESM10_ESM.zip › Figure 6/6D/2024-07-12_Replicate1_cAMPSeperated/contrast/contrast_5.png]

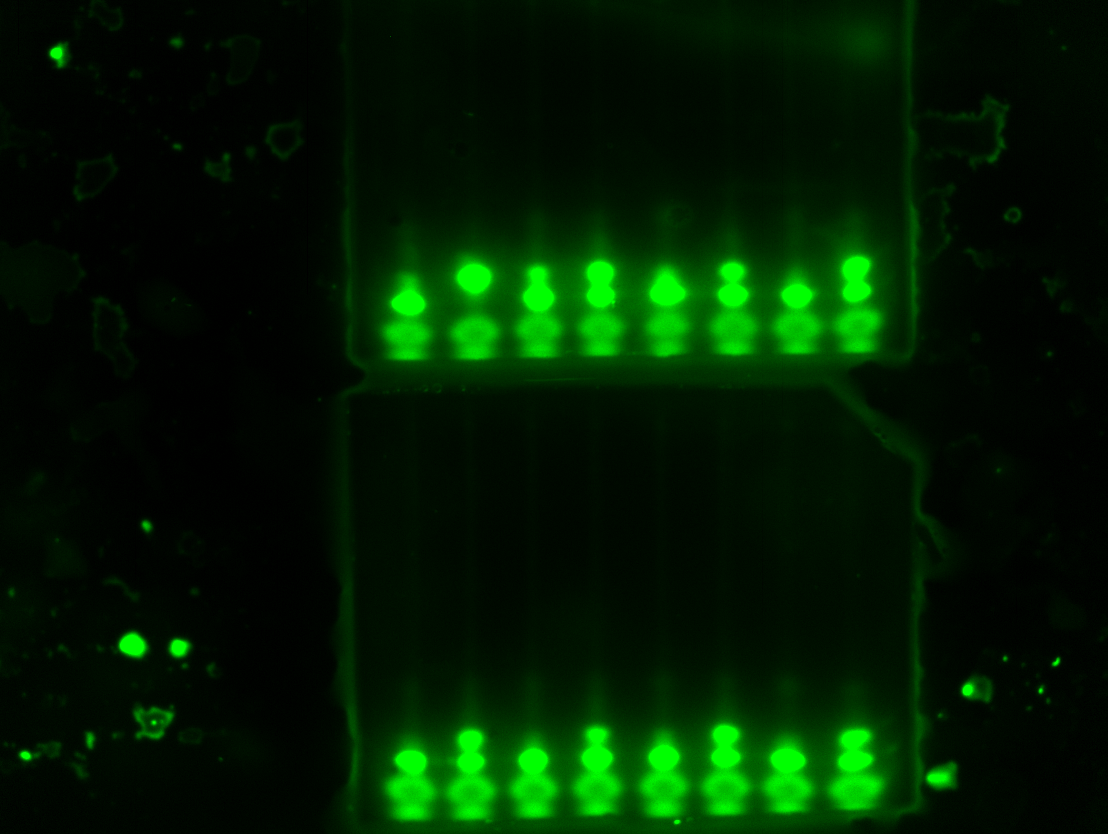

Supplement: Supplementary file 10 — Source data Fig. 6 [file 44318_2025_436_MOESM10_ESM.zip › Figure 6/6D/2024-07-12_Replicate1_cAMPSeperated/contrast/contrast_4.png]

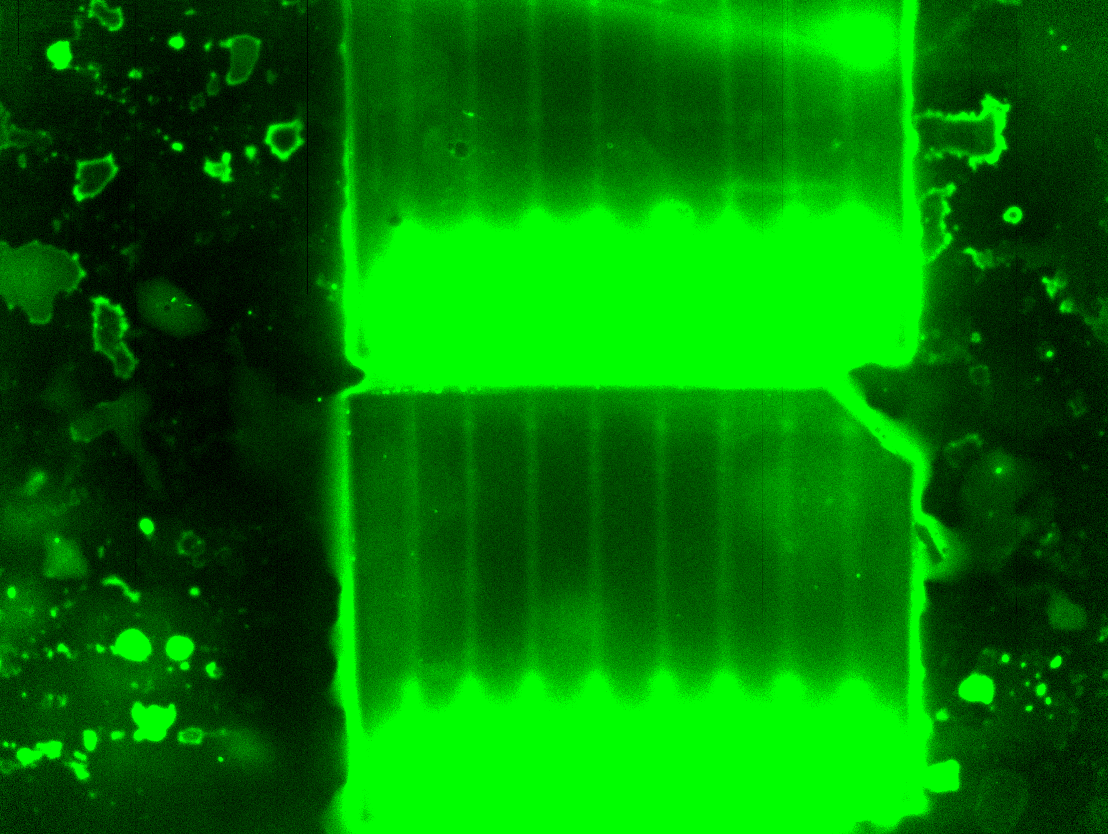

Supplement: Supplementary file 10 — Source data Fig. 6 [file 44318_2025_436_MOESM10_ESM.zip › Figure 6/6D/2024-07-12_Replicate1_cAMPSeperated/contrast/contrast_8.png]

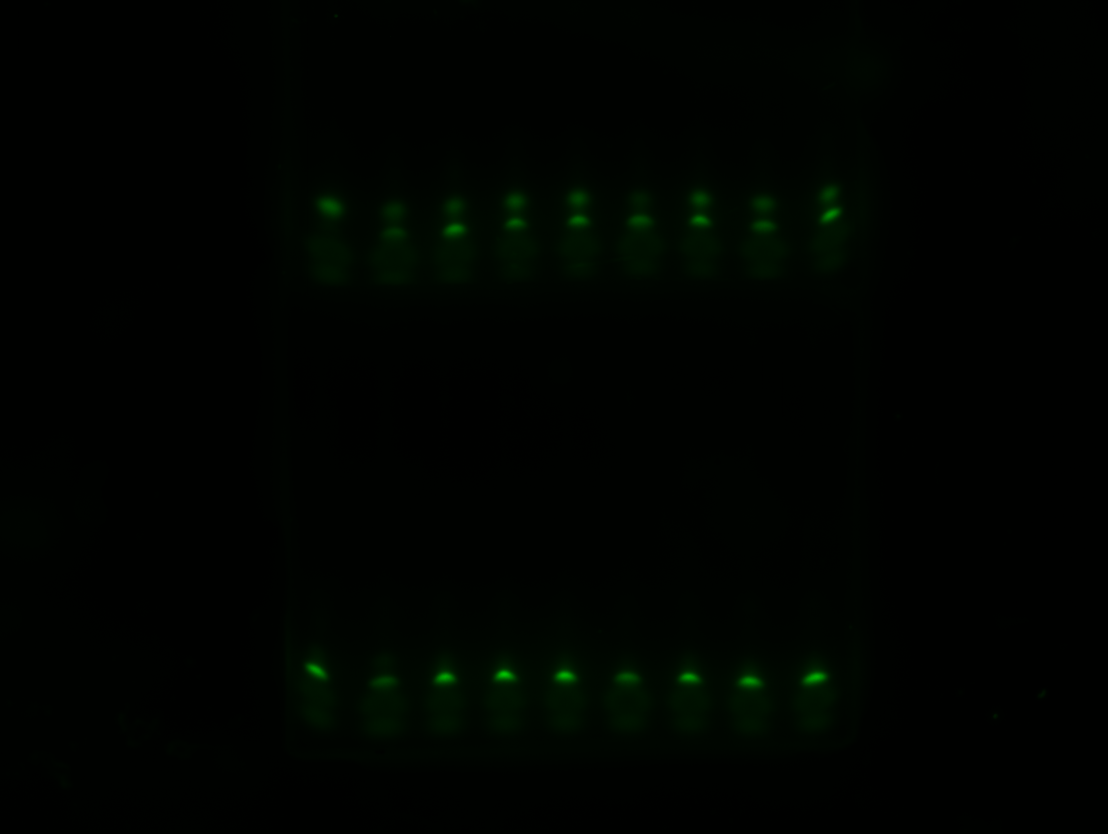

Supplement: Supplementary file 10 — Source data Fig. 6 [file 44318_2025_436_MOESM10_ESM.zip › Figure 6/6D/2024-07-12_Replicate3/contrast/contrast_0.png]

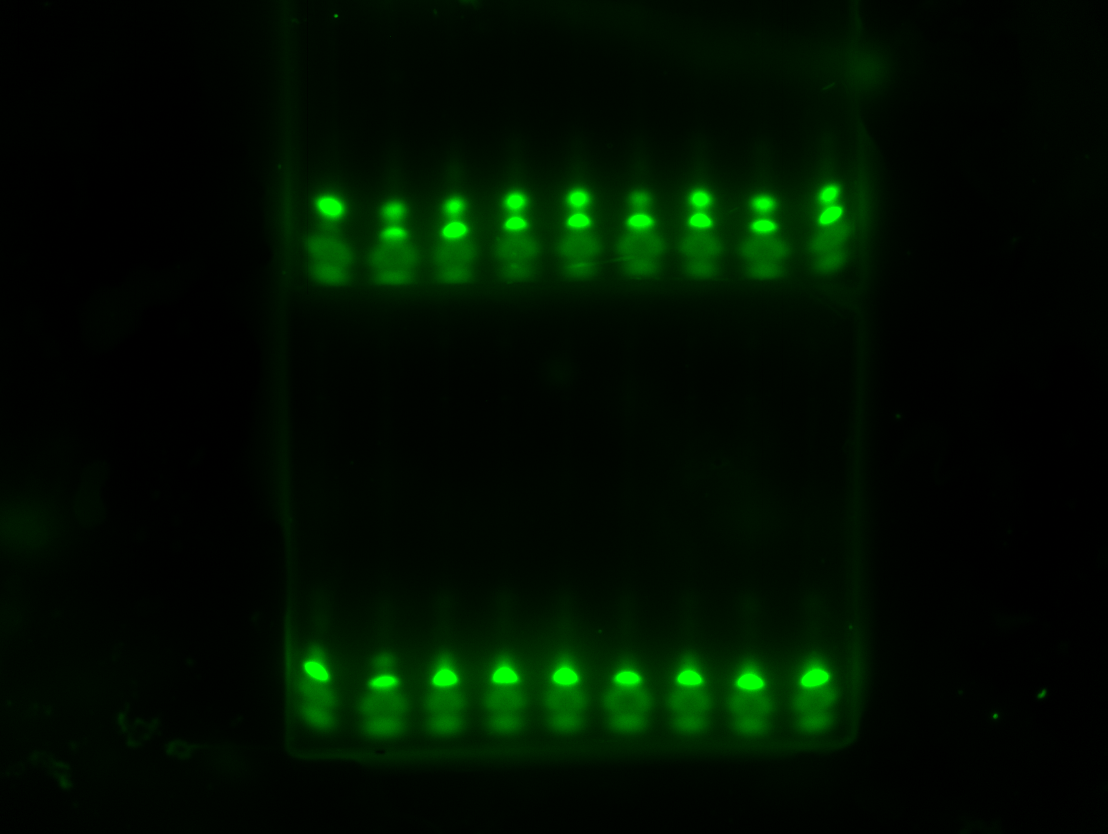

Supplement: Supplementary file 10 — Source data Fig. 6 [file 44318_2025_436_MOESM10_ESM.zip › Figure 6/6D/2024-07-12_Replicate3/contrast/contrast_3.png]

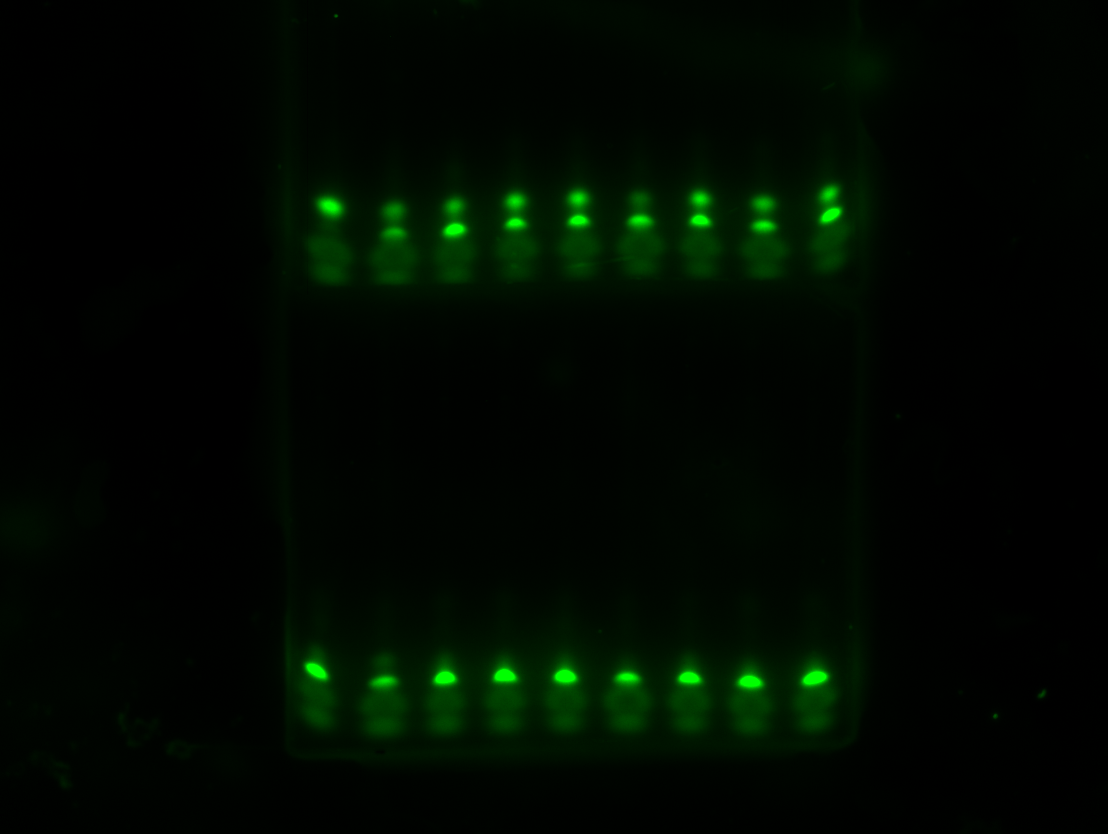

Supplement: Supplementary file 10 — Source data Fig. 6 [file 44318_2025_436_MOESM10_ESM.zip › Figure 6/6D/2024-07-12_Replicate3/contrast/contrast_2.png]

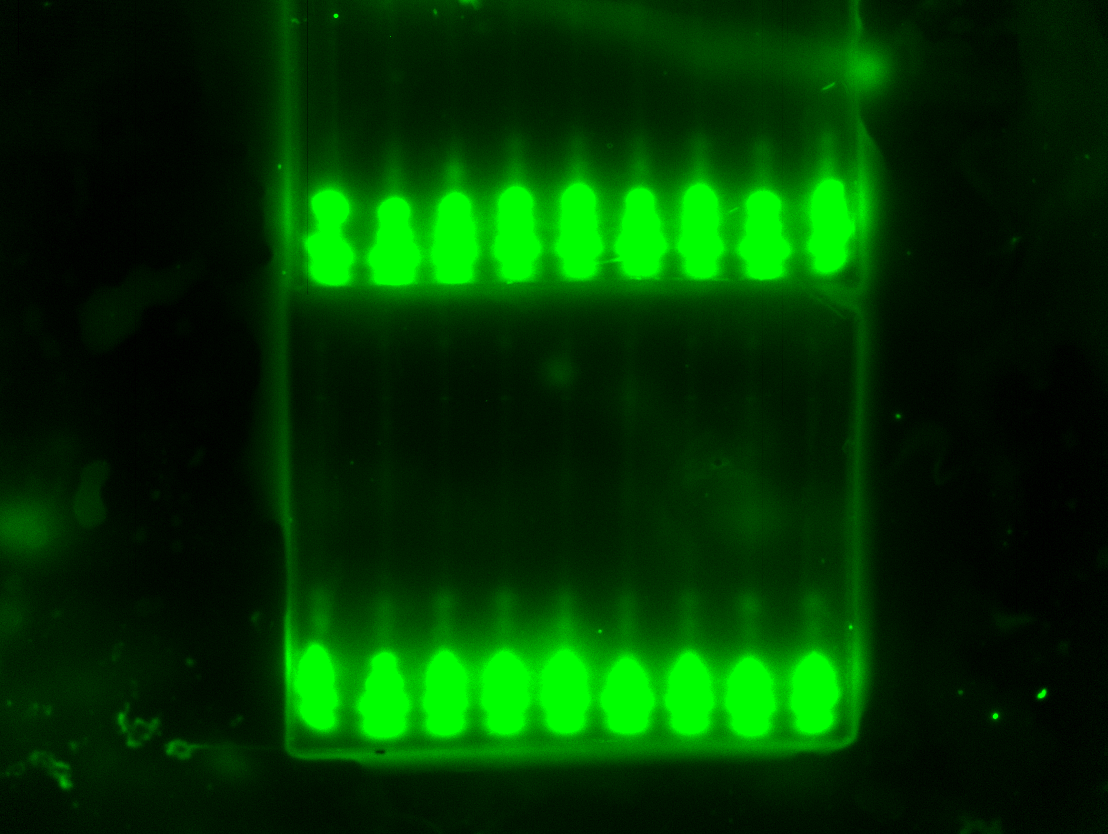

Supplement: Supplementary file 10 — Source data Fig. 6 [file 44318_2025_436_MOESM10_ESM.zip › Figure 6/6D/2024-07-12_Replicate3/contrast/contrast_6.png]

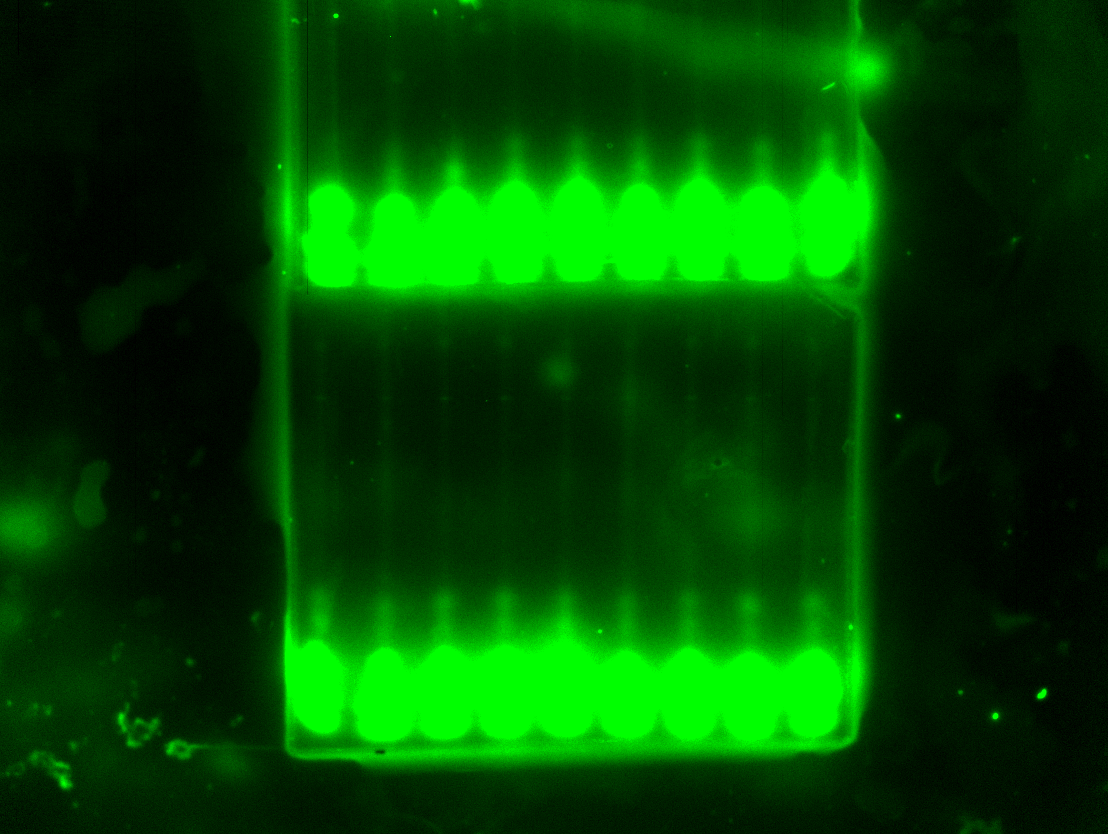

Supplement: Supplementary file 10 — Source data Fig. 6 [file 44318_2025_436_MOESM10_ESM.zip › Figure 6/6D/2024-07-12_Replicate3/contrast/contrast_7.png]

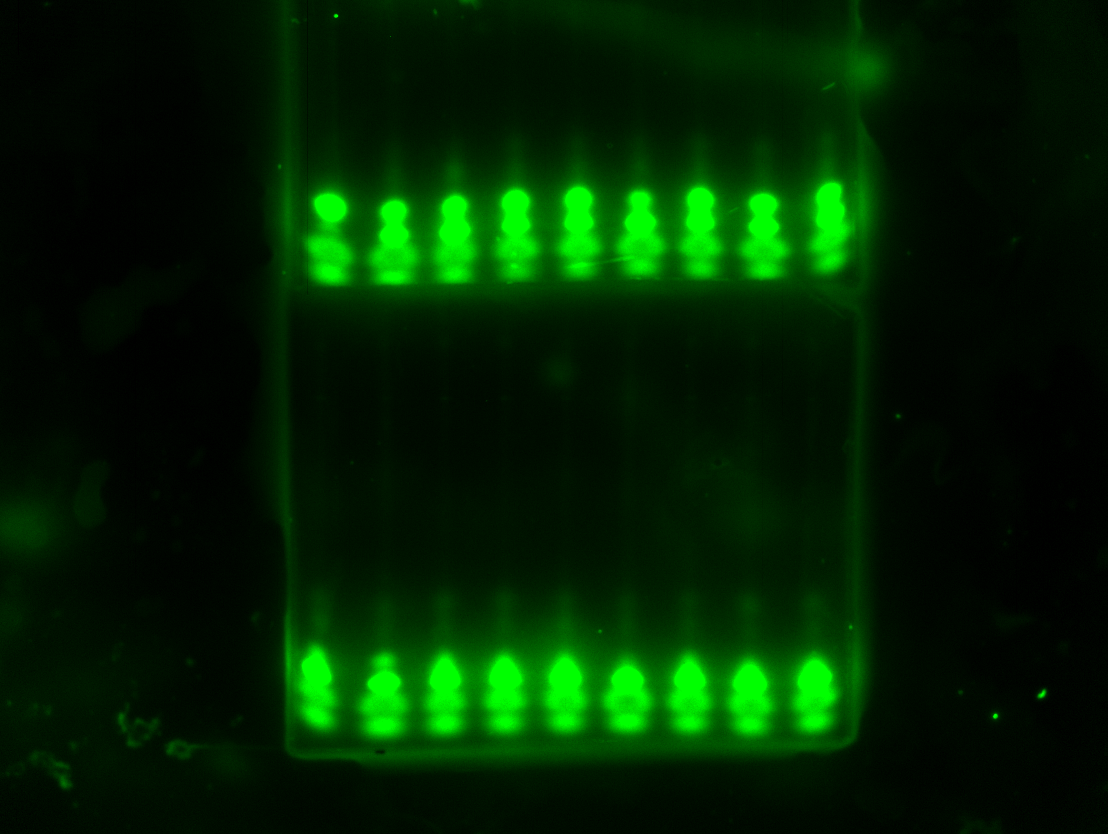

Supplement: Supplementary file 10 — Source data Fig. 6 [file 44318_2025_436_MOESM10_ESM.zip › Figure 6/6D/2024-07-12_Replicate3/contrast/contrast_5.png]

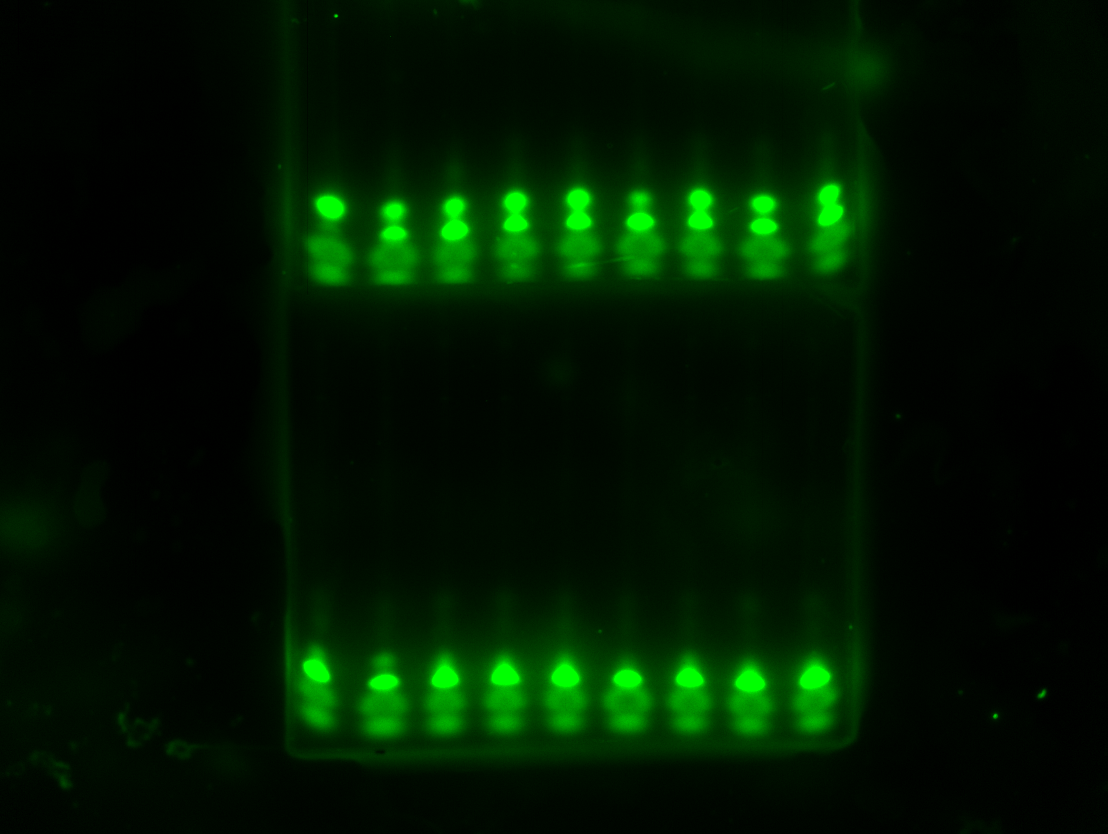

Supplement: Supplementary file 10 — Source data Fig. 6 [file 44318_2025_436_MOESM10_ESM.zip › Figure 6/6D/2024-07-12_Replicate3/contrast/contrast_4.png]

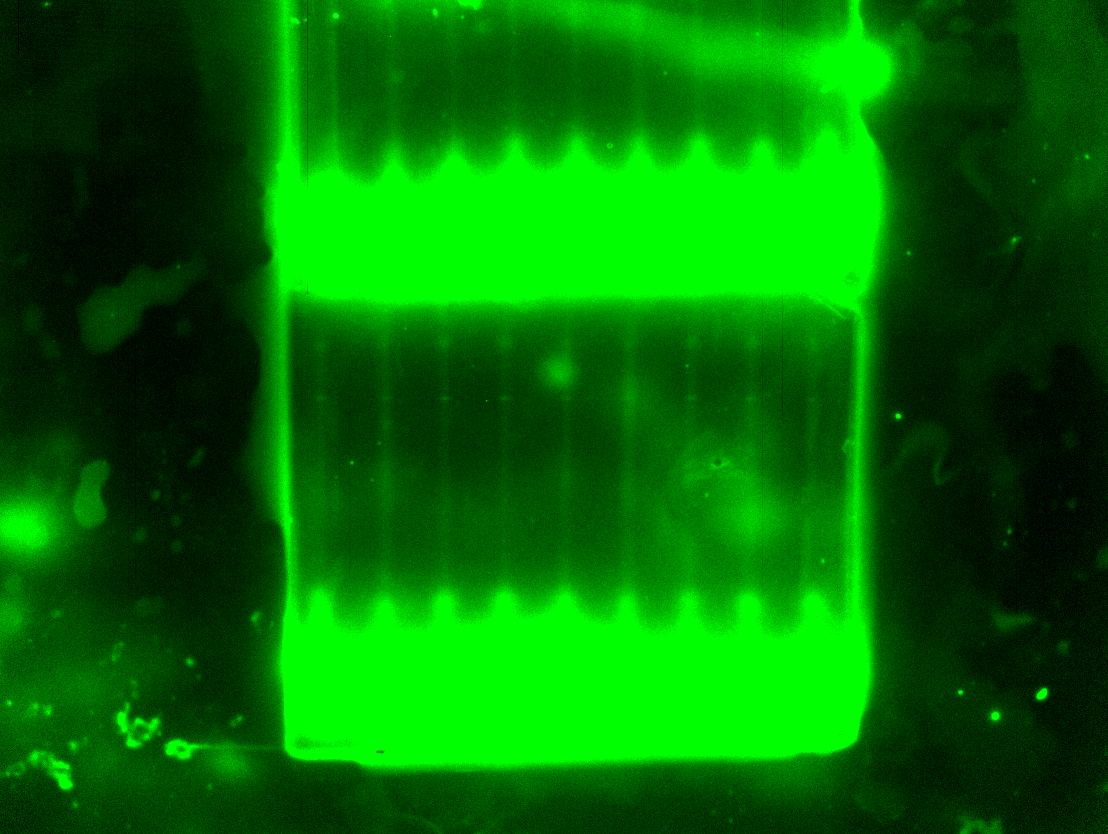

Supplement: Supplementary file 10 — Source data Fig. 6 [file 44318_2025_436_MOESM10_ESM.zip › Figure 6/6D/2024-07-12_Replicate3/contrast/contrast_8.png]

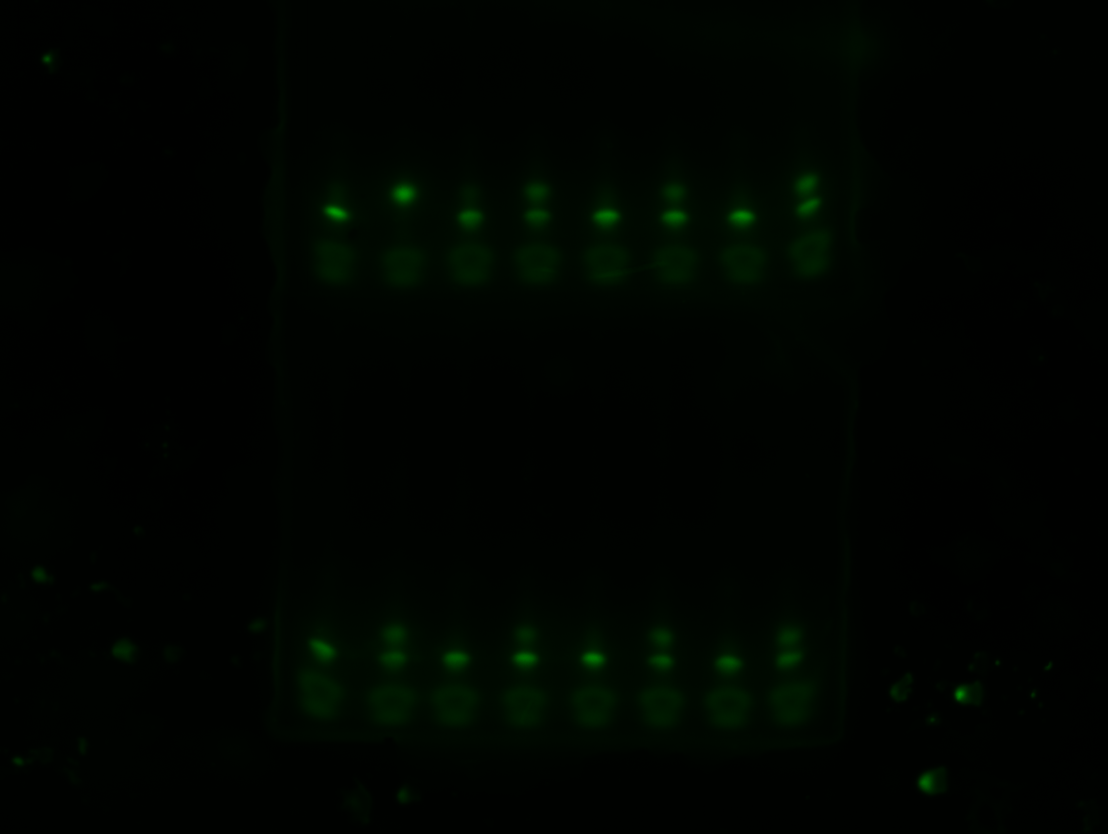

Supplement: Supplementary file 10 — Source data Fig. 6 [file 44318_2025_436_MOESM10_ESM.zip › Figure 6/6D/2024-07-12_Replicate2/contrast/contrast_0.png]

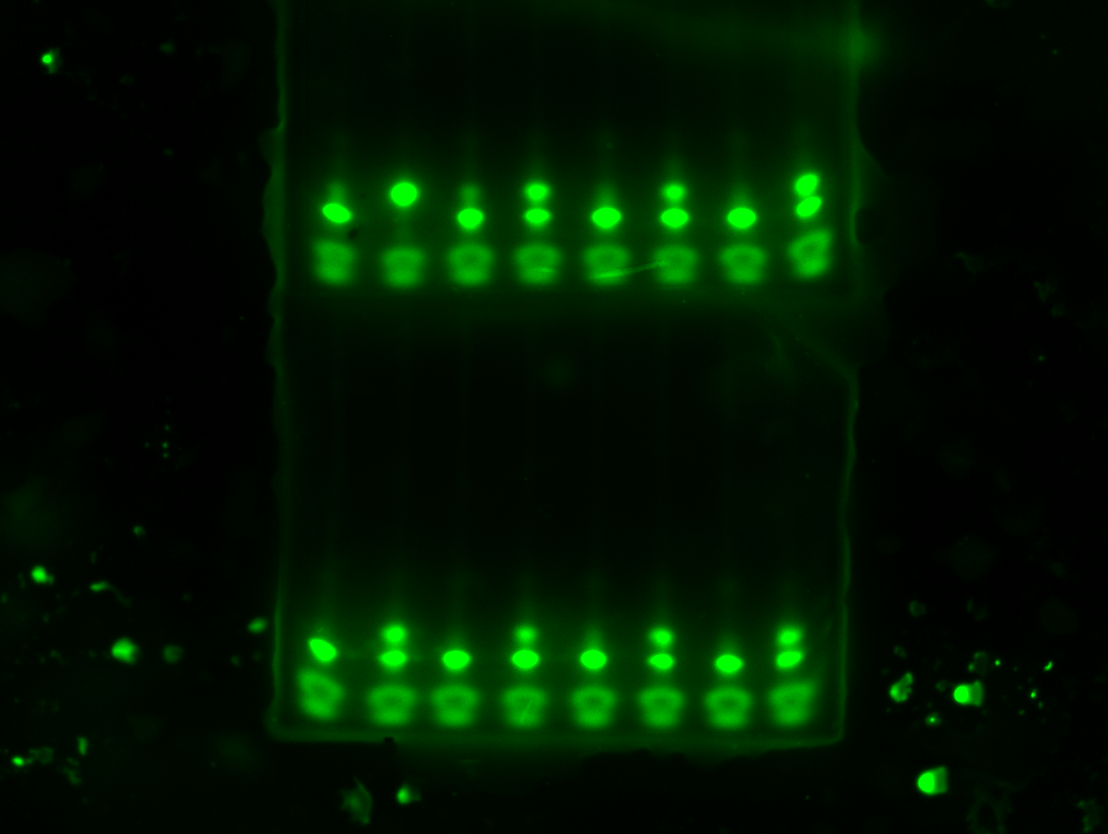

Supplement: Supplementary file 10 — Source data Fig. 6 [file 44318_2025_436_MOESM10_ESM.zip › Figure 6/6D/2024-07-12_Replicate2/contrast/contrast_3.png]

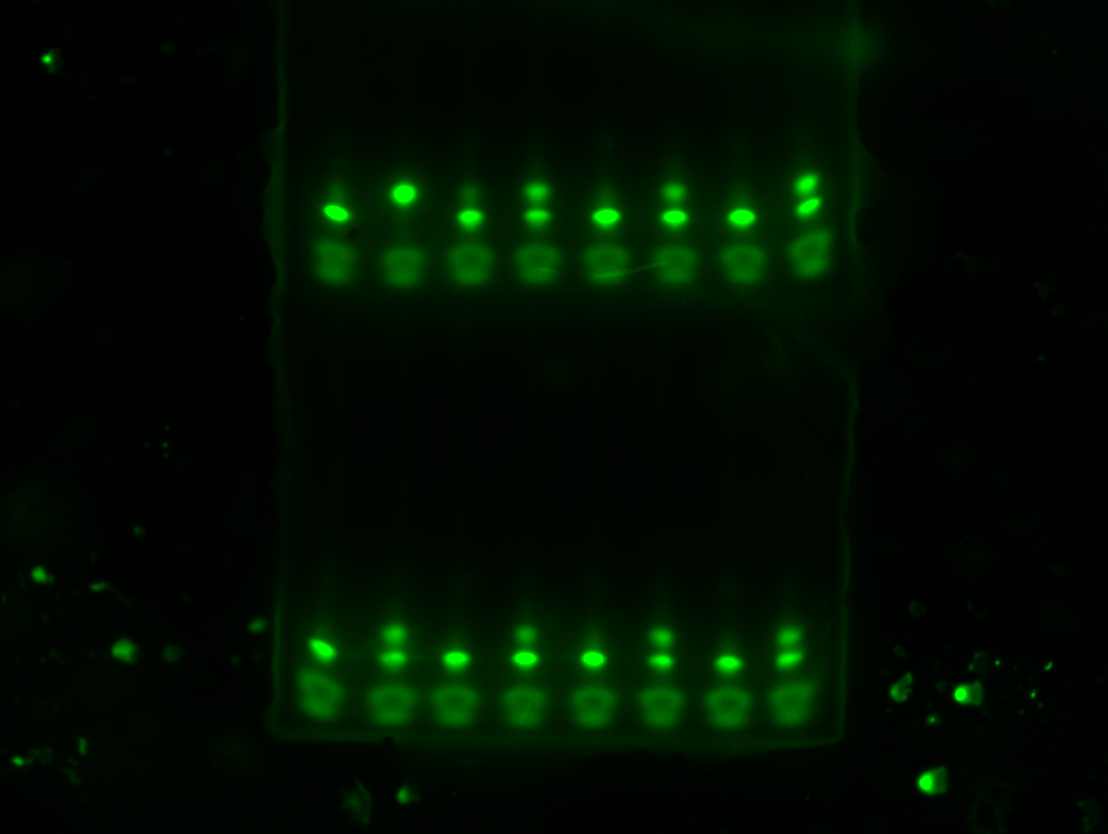

Supplement: Supplementary file 10 — Source data Fig. 6 [file 44318_2025_436_MOESM10_ESM.zip › Figure 6/6D/2024-07-12_Replicate2/contrast/contrast_2.png]

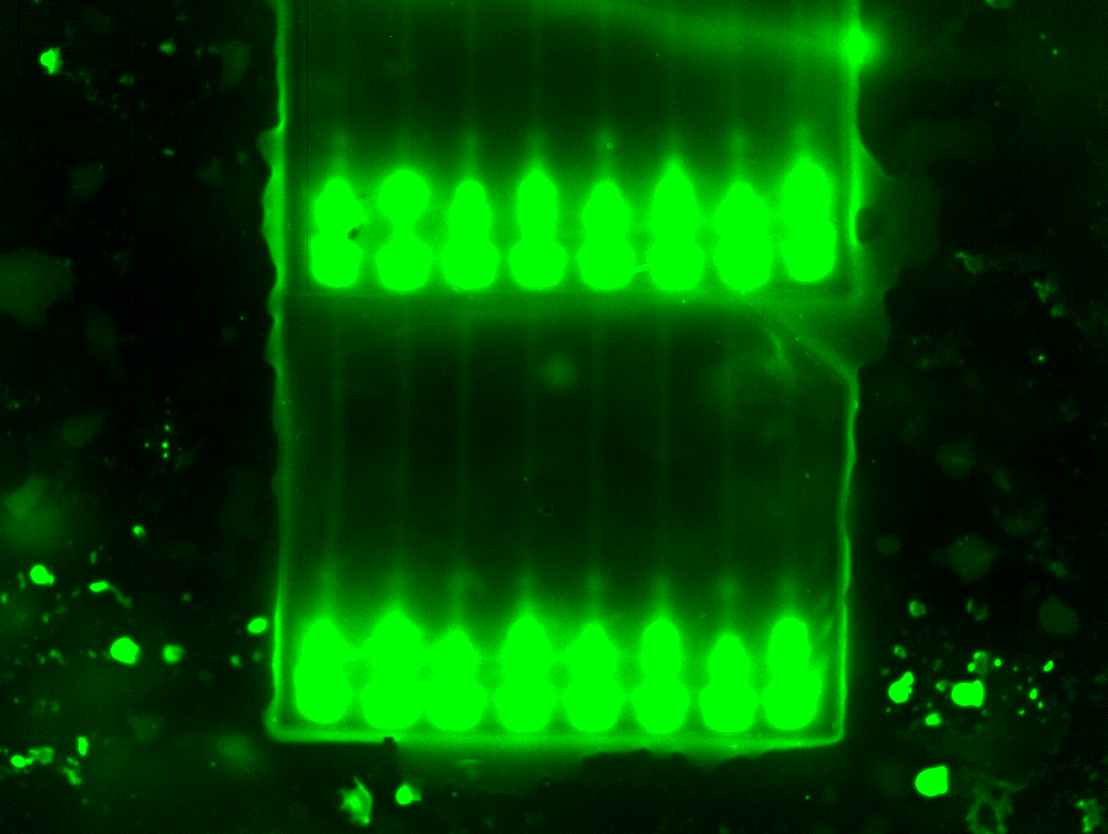

Supplement: Supplementary file 10 — Source data Fig. 6 [file 44318_2025_436_MOESM10_ESM.zip › Figure 6/6D/2024-07-12_Replicate2/contrast/contrast_6.png]

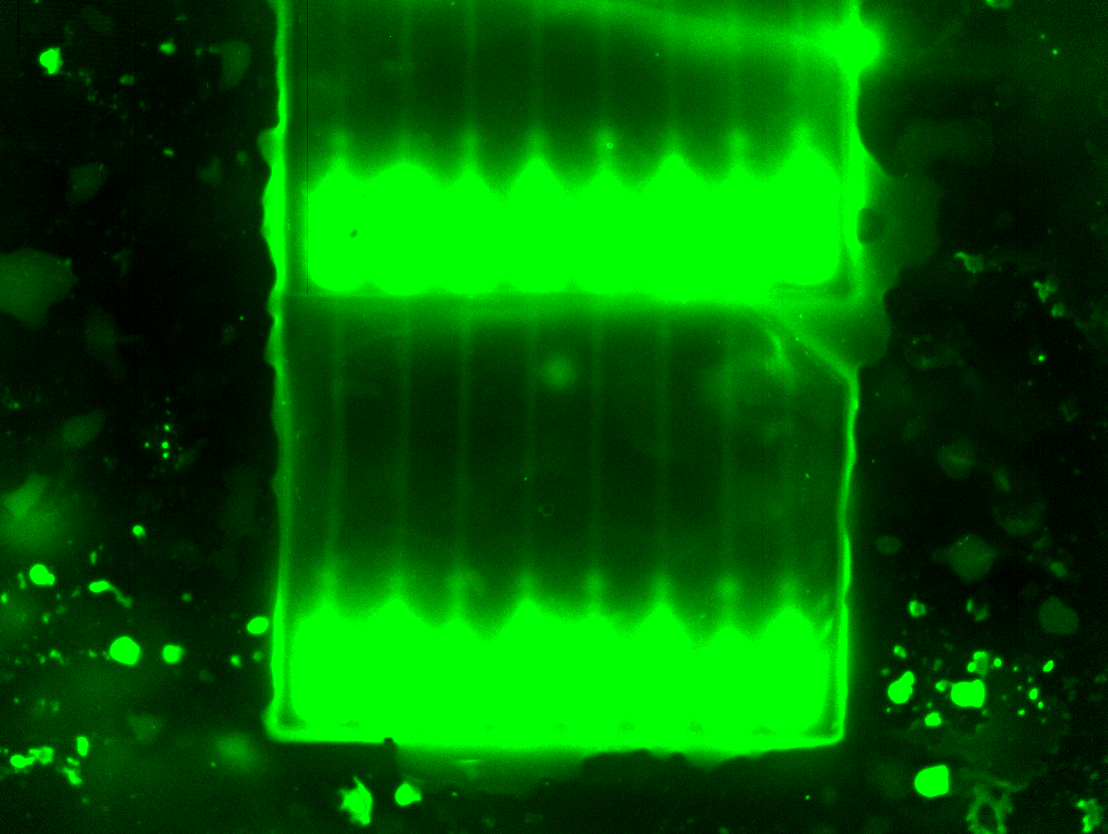

Supplement: Supplementary file 10 — Source data Fig. 6 [file 44318_2025_436_MOESM10_ESM.zip › Figure 6/6D/2024-07-12_Replicate2/contrast/contrast_7.png]

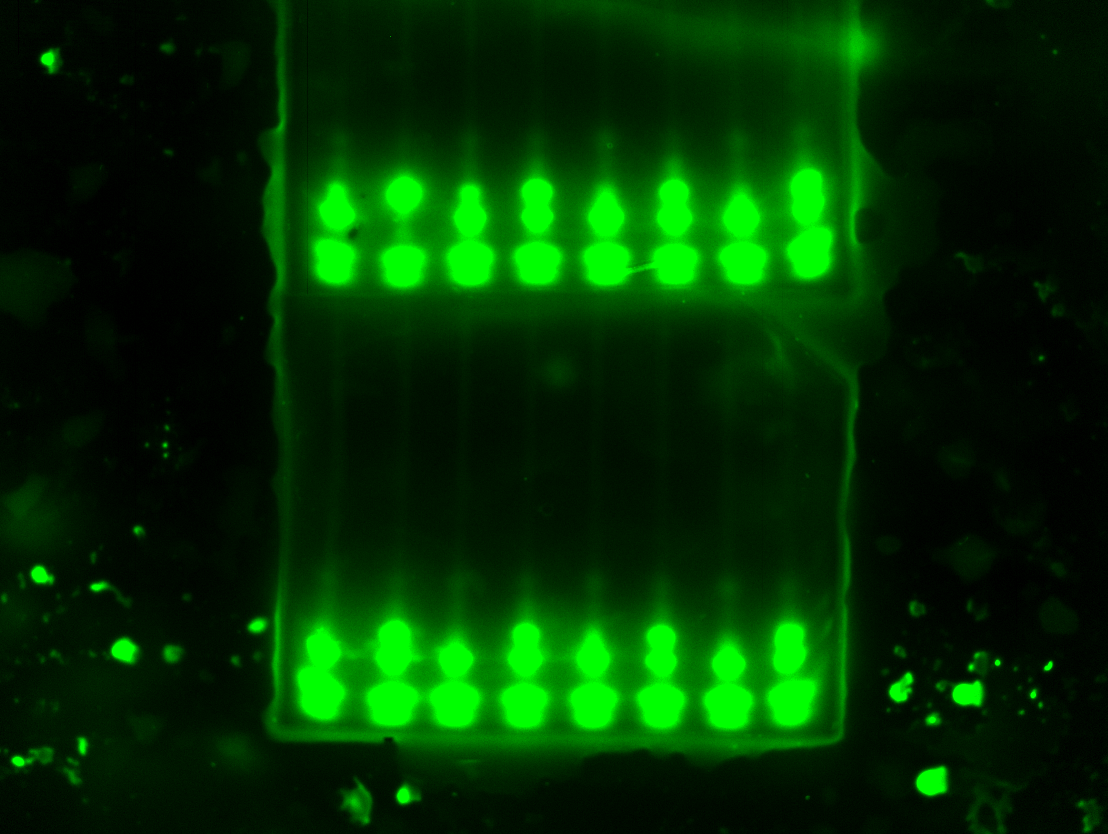

Supplement: Supplementary file 10 — Source data Fig. 6 [file 44318_2025_436_MOESM10_ESM.zip › Figure 6/6D/2024-07-12_Replicate2/contrast/contrast_5.png]

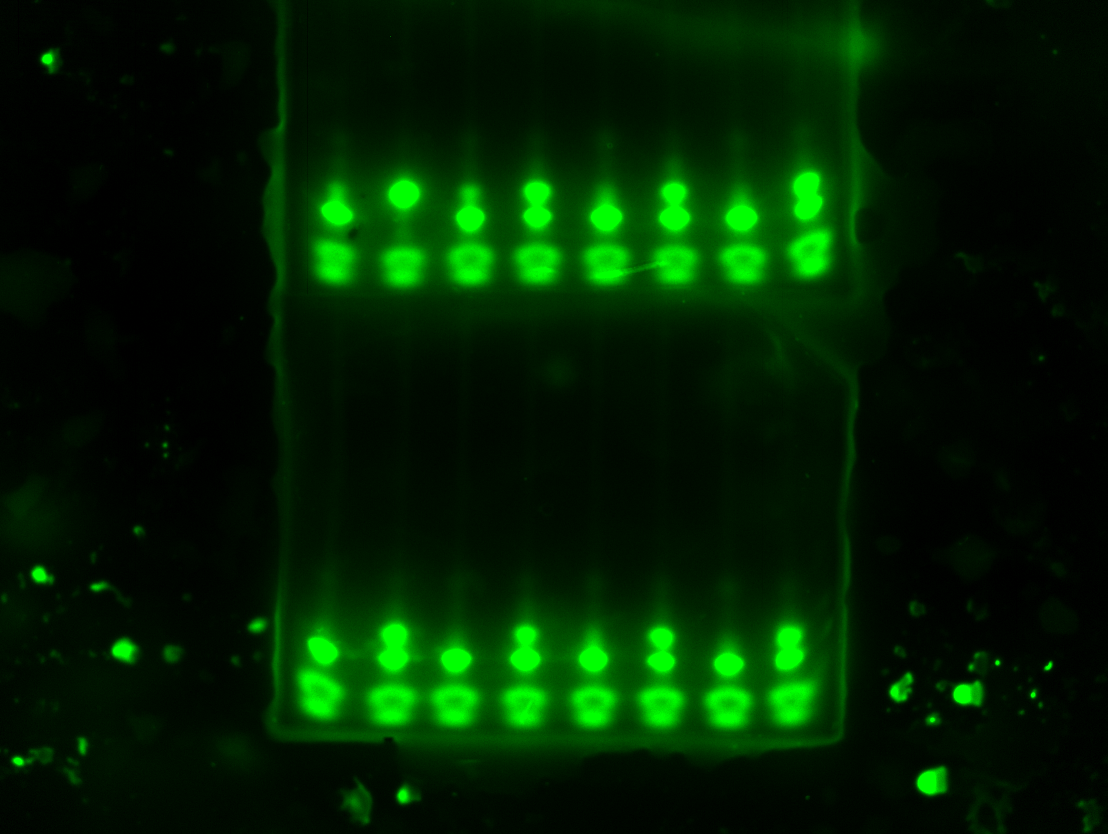

Supplement: Supplementary file 10 — Source data Fig. 6 [file 44318_2025_436_MOESM10_ESM.zip › Figure 6/6D/2024-07-12_Replicate2/contrast/contrast_4.png]

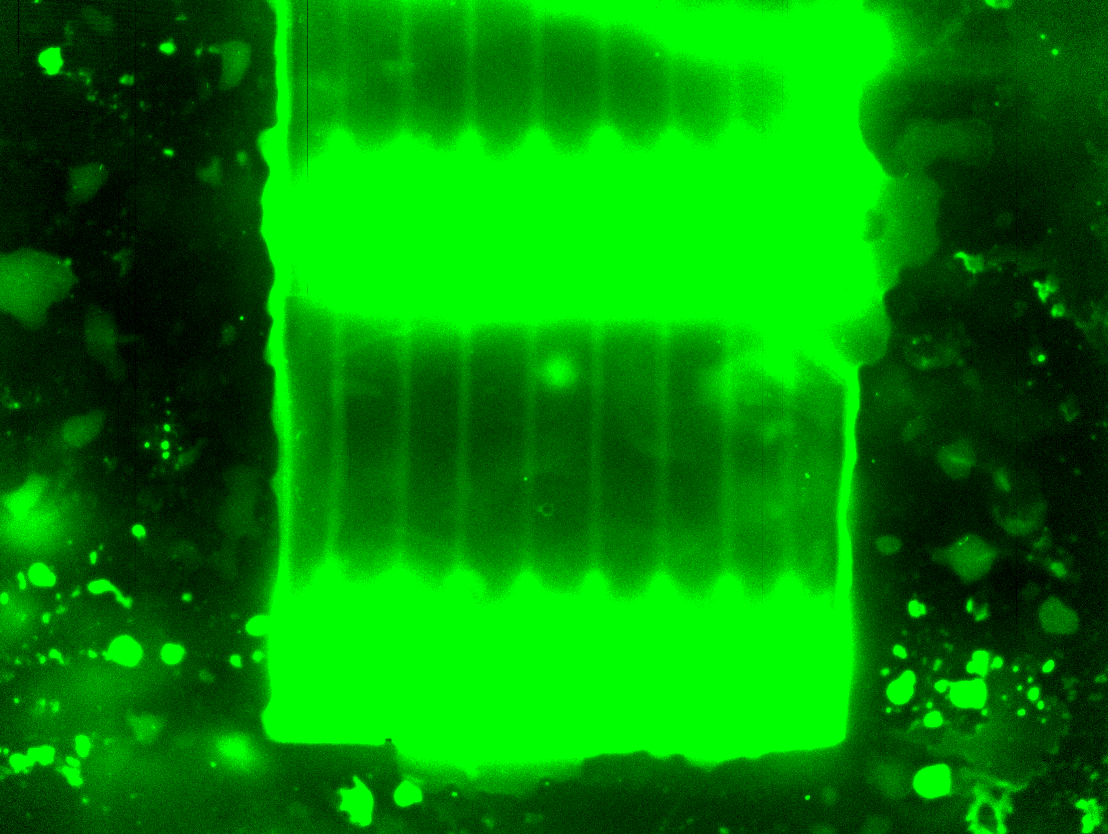

Supplement: Supplementary file 10 — Source data Fig. 6 [file 44318_2025_436_MOESM10_ESM.zip › Figure 6/6D/2024-07-12_Replicate2/contrast/contrast_8.png]
