# Supplementary figures and images for: Autophagosomes anchor an AKAP11-dependent regulatory checkpoint that shapes neuronal PKA signaling (part 2 of 2)
Source: EMBO J. 2025 Apr 22;44(11):3150–79. doi: 10.1038/s44318-025-00436-x (PMC12130464; doi:10.1038/s44318-025-00436-x)

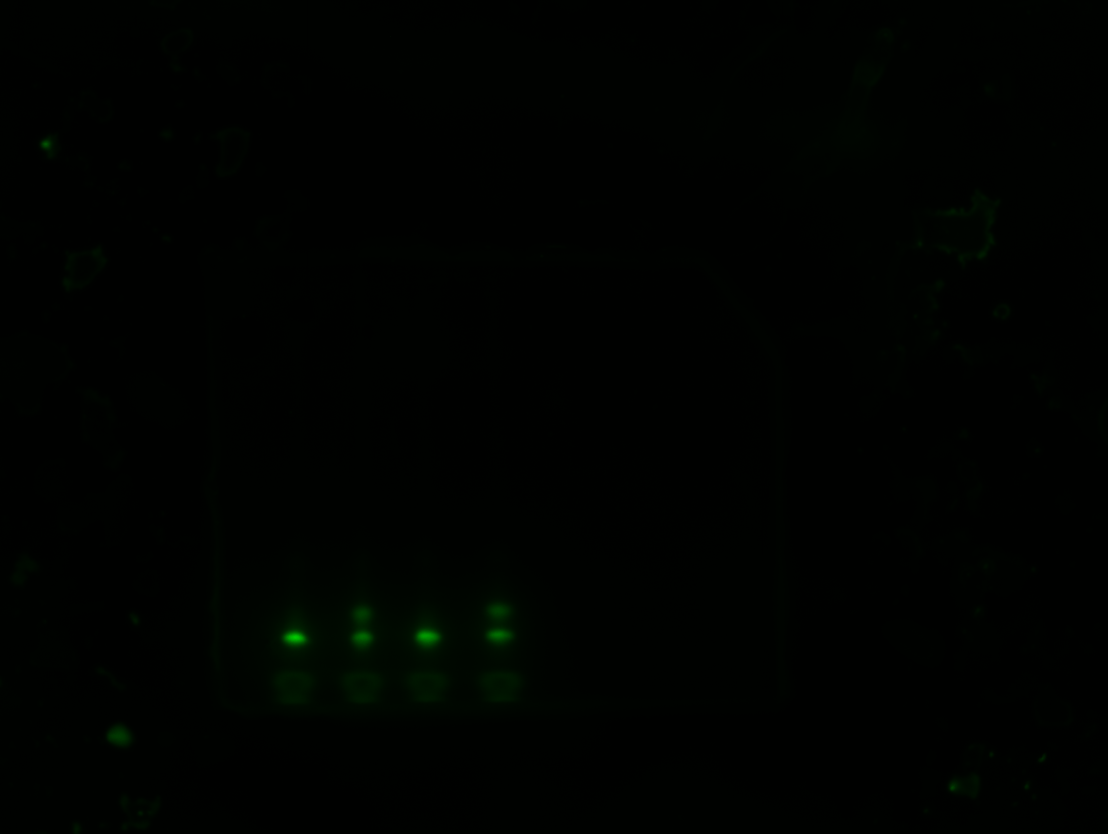

Supplement: Supplementary file 10 — Source data Fig. 6 [file 44318_2025_436_MOESM10_ESM.zip › Figure 6/6D/2024-07-12_Replicate2_3T338D/contrast/contrast_0.png]

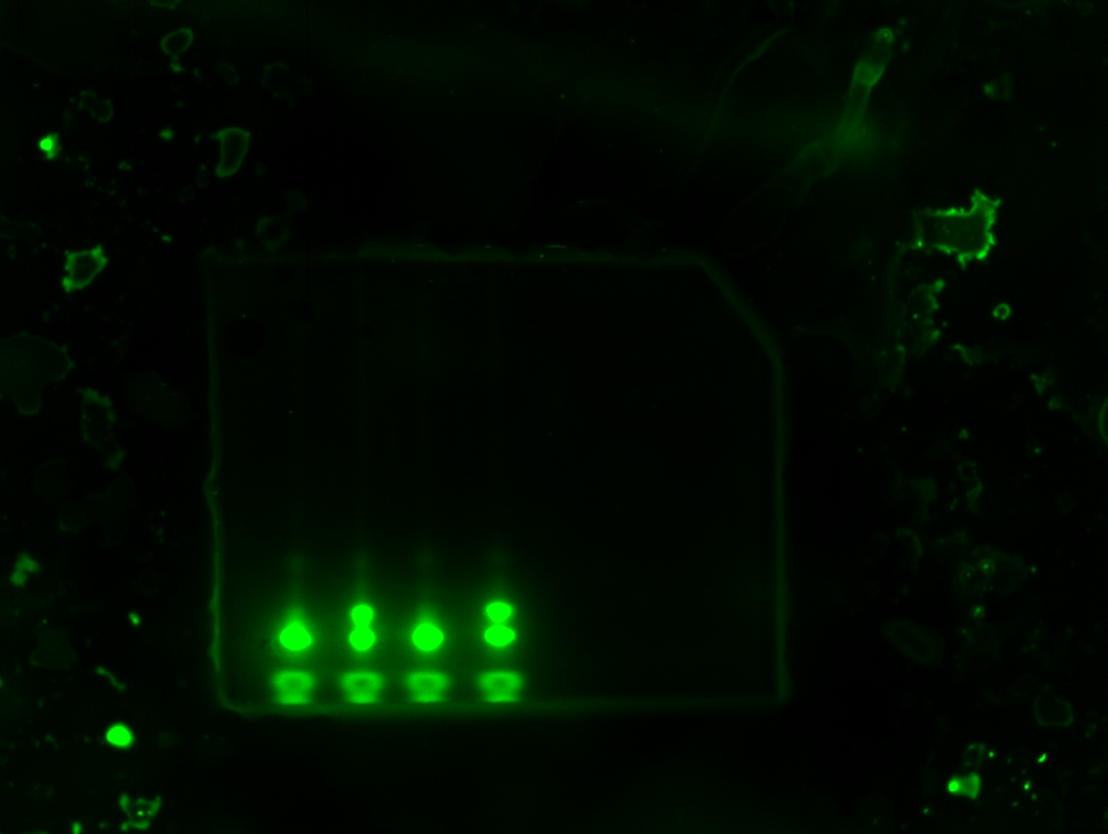

Supplement: Supplementary file 10 — Source data Fig. 6 [file 44318_2025_436_MOESM10_ESM.zip › Figure 6/6D/2024-07-12_Replicate2_3T338D/contrast/contrast_3.png]

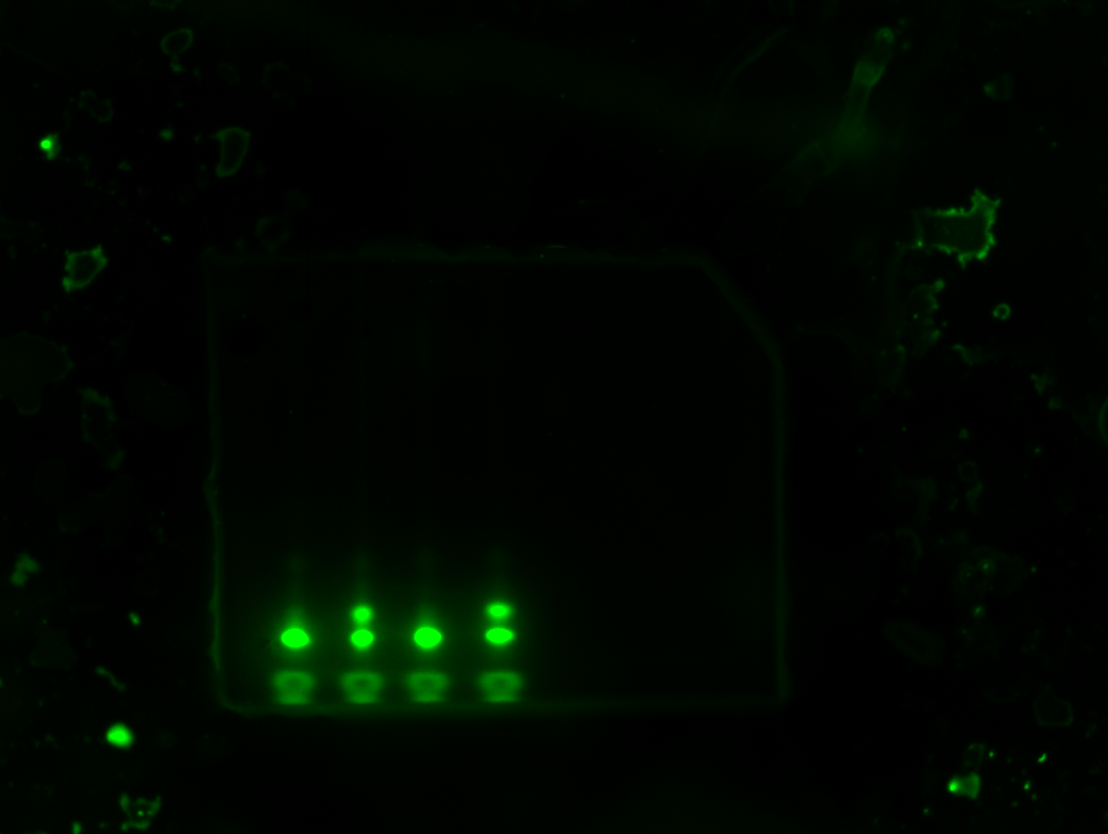

Supplement: Supplementary file 10 — Source data Fig. 6 [file 44318_2025_436_MOESM10_ESM.zip › Figure 6/6D/2024-07-12_Replicate2_3T338D/contrast/contrast_2.png]

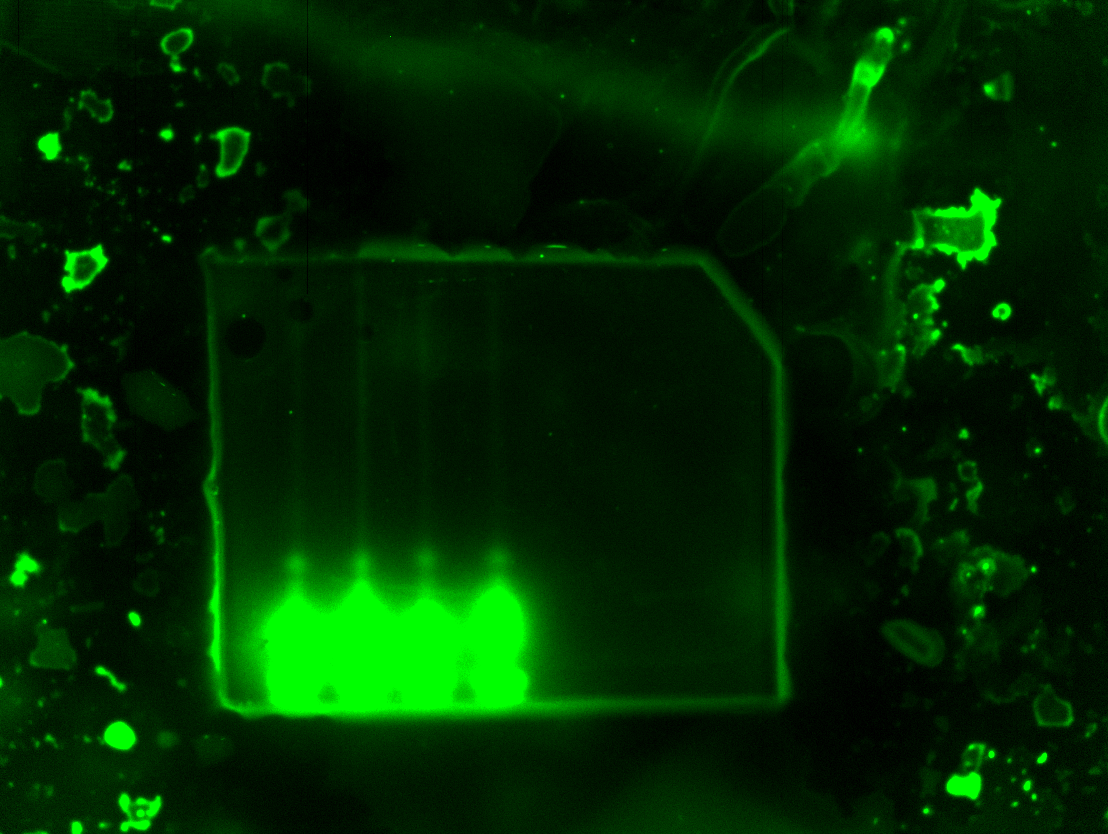

Supplement: Supplementary file 10 — Source data Fig. 6 [file 44318_2025_436_MOESM10_ESM.zip › Figure 6/6D/2024-07-12_Replicate2_3T338D/contrast/contrast_6.png]

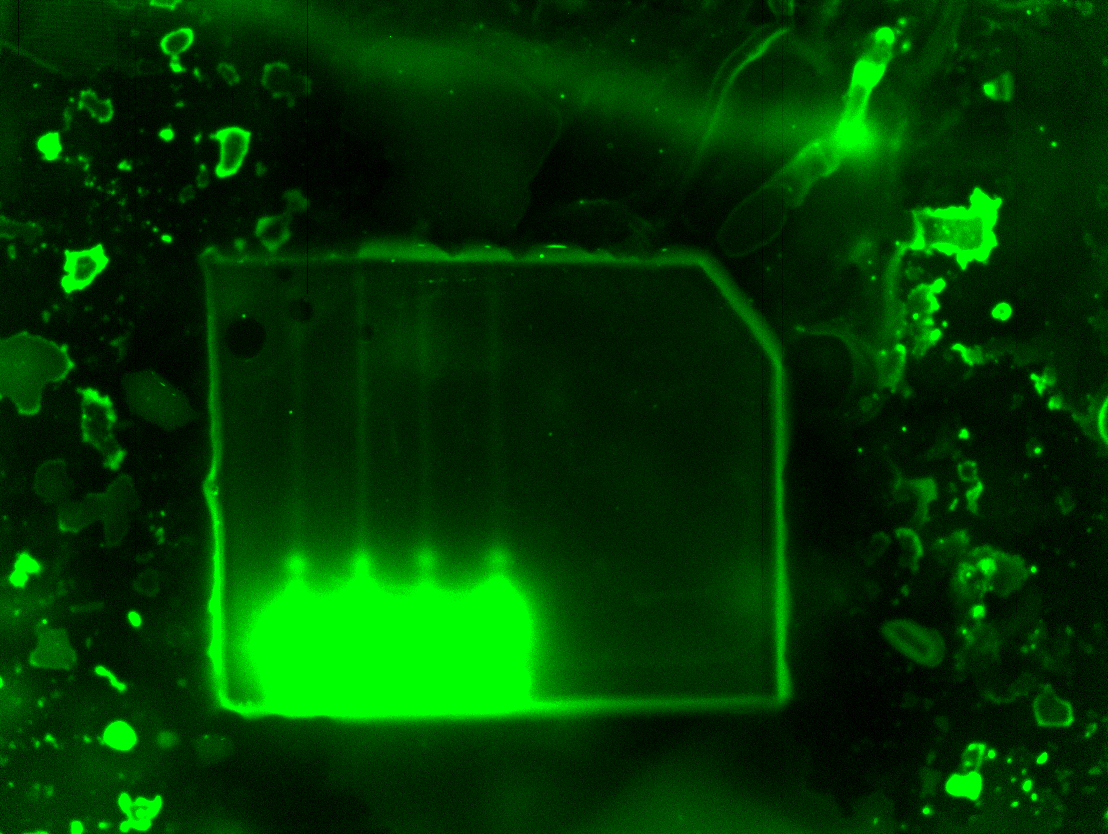

Supplement: Supplementary file 10 — Source data Fig. 6 [file 44318_2025_436_MOESM10_ESM.zip › Figure 6/6D/2024-07-12_Replicate2_3T338D/contrast/contrast_7.png]

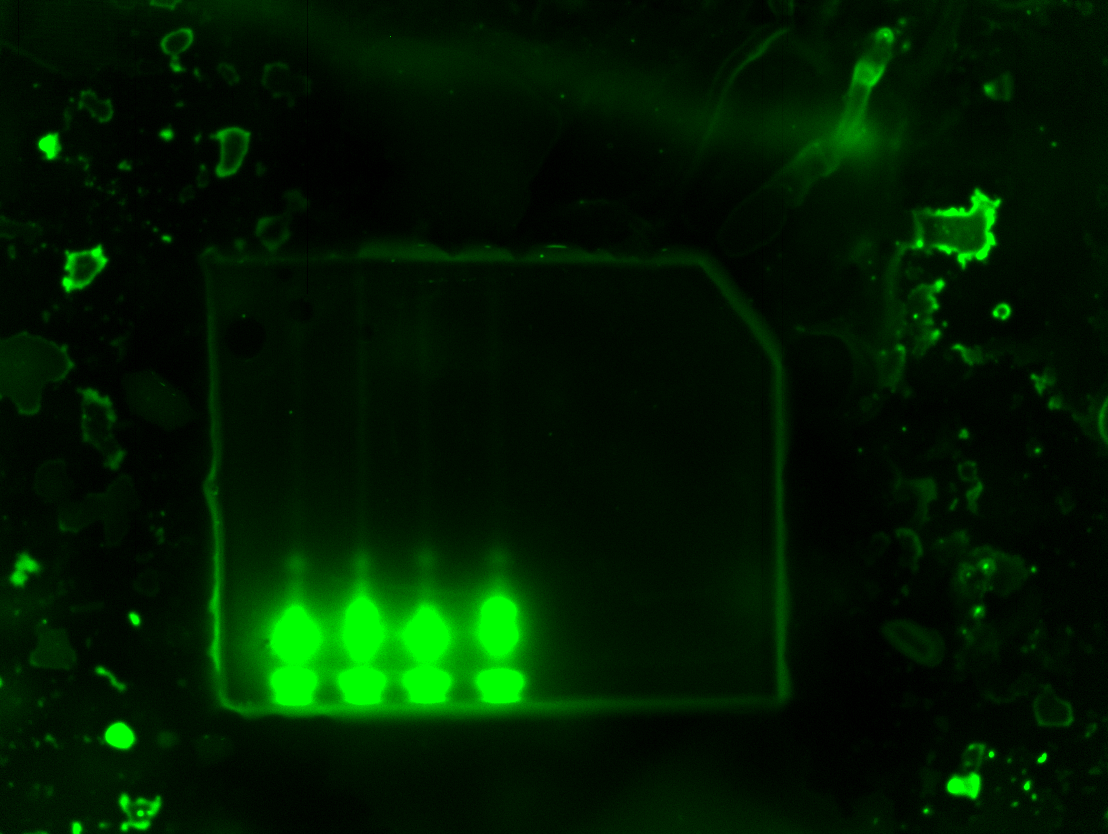

Supplement: Supplementary file 10 — Source data Fig. 6 [file 44318_2025_436_MOESM10_ESM.zip › Figure 6/6D/2024-07-12_Replicate2_3T338D/contrast/contrast_5.png]

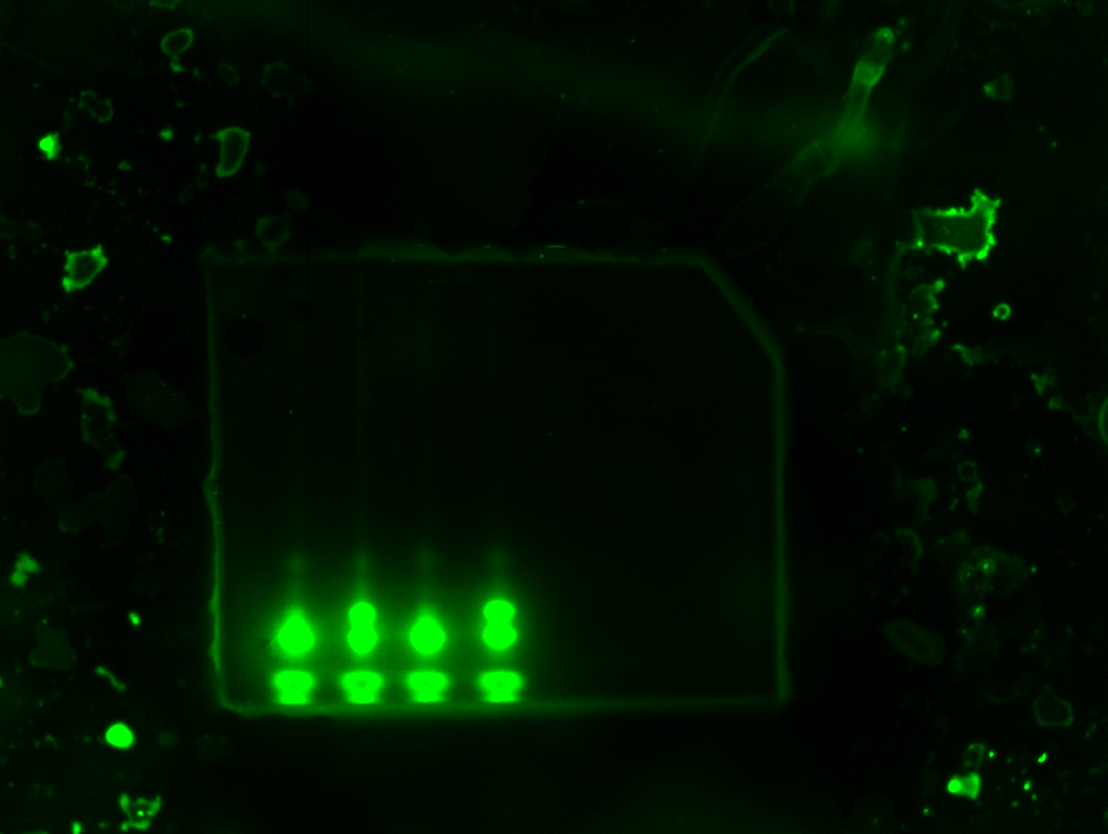

Supplement: Supplementary file 10 — Source data Fig. 6 [file 44318_2025_436_MOESM10_ESM.zip › Figure 6/6D/2024-07-12_Replicate2_3T338D/contrast/contrast_4.png]

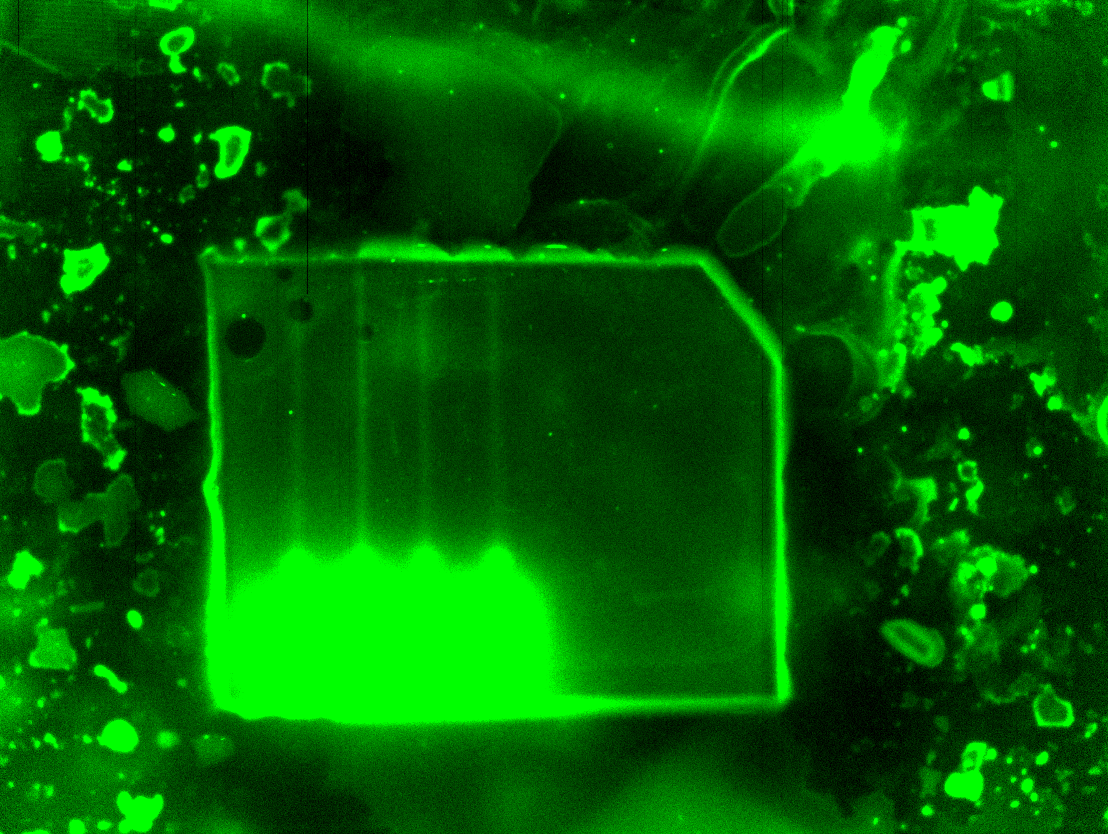

Supplement: Supplementary file 10 — Source data Fig. 6 [file 44318_2025_436_MOESM10_ESM.zip › Figure 6/6D/2024-07-12_Replicate2_3T338D/contrast/contrast_8.png]
