# Supplementary material for: Autophagosomes anchor an AKAP11-dependent regulatory checkpoint that shapes neuronal PKA signaling
Source: EMBO J. 2025 Apr 22;44(11):3150–79. doi: 10.1038/s44318-025-00436-x (PMC12130464; doi:10.1038/s44318-025-00436-x)
Supplement: Supplementary file 11 — Expanded View Figures [file 44318_2025_436_MOESM11_ESM.pdf]

Expanded View Figures

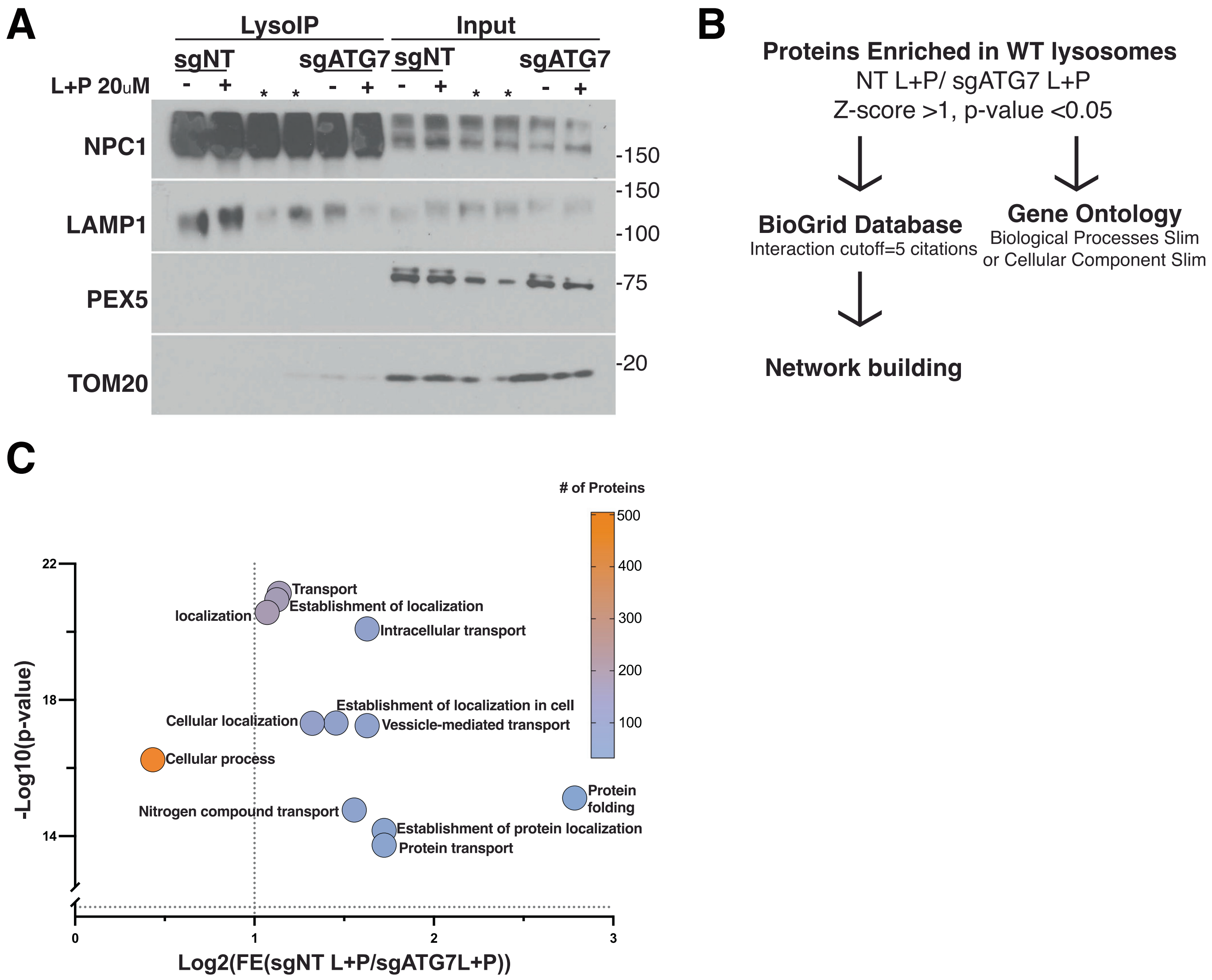

Figure EV1. Lysosomal immunoisolation and network building.

(**A**) Immunoblots of Lysosomal immunoprecipitation and corresponding input from HEK-293T-sgNT or sgATG7 after treatment 20 μM Leupeptin and 20 μM Pepstatin for 24 h to block lysosomal degradation of lysosomal substrates. (**B**) Outline depicting bioinformatic pipeline in which proteins identified as 'Hits' (Log2(FC[sgNT L + P/ sgATG7 L + P]) > 1, *P* value < 0.05, two-tailed *t* test) in LysoIP from WT and ATG7-null cells were subjected to custom-written pipeline where interactors of Hits (using citation cutoff of ≥5 citations) were identified using data from BioGrid. List of protein 'Hits' were also entered in panther to generate a gene ontology analysis for enrichment of biological processes or enrichment of cellular component. (**C**) Volcano plot of "biological processes slim" Go-terms of proteins enriched in wild-type lysosomes compared to autophagy-null lysosomes in 20 μM L + P 24-hour treatment. Protein list obtained from proteins identified as 'Hits'. *P* value calculated using Fishers exact test.

 © The Author(s)

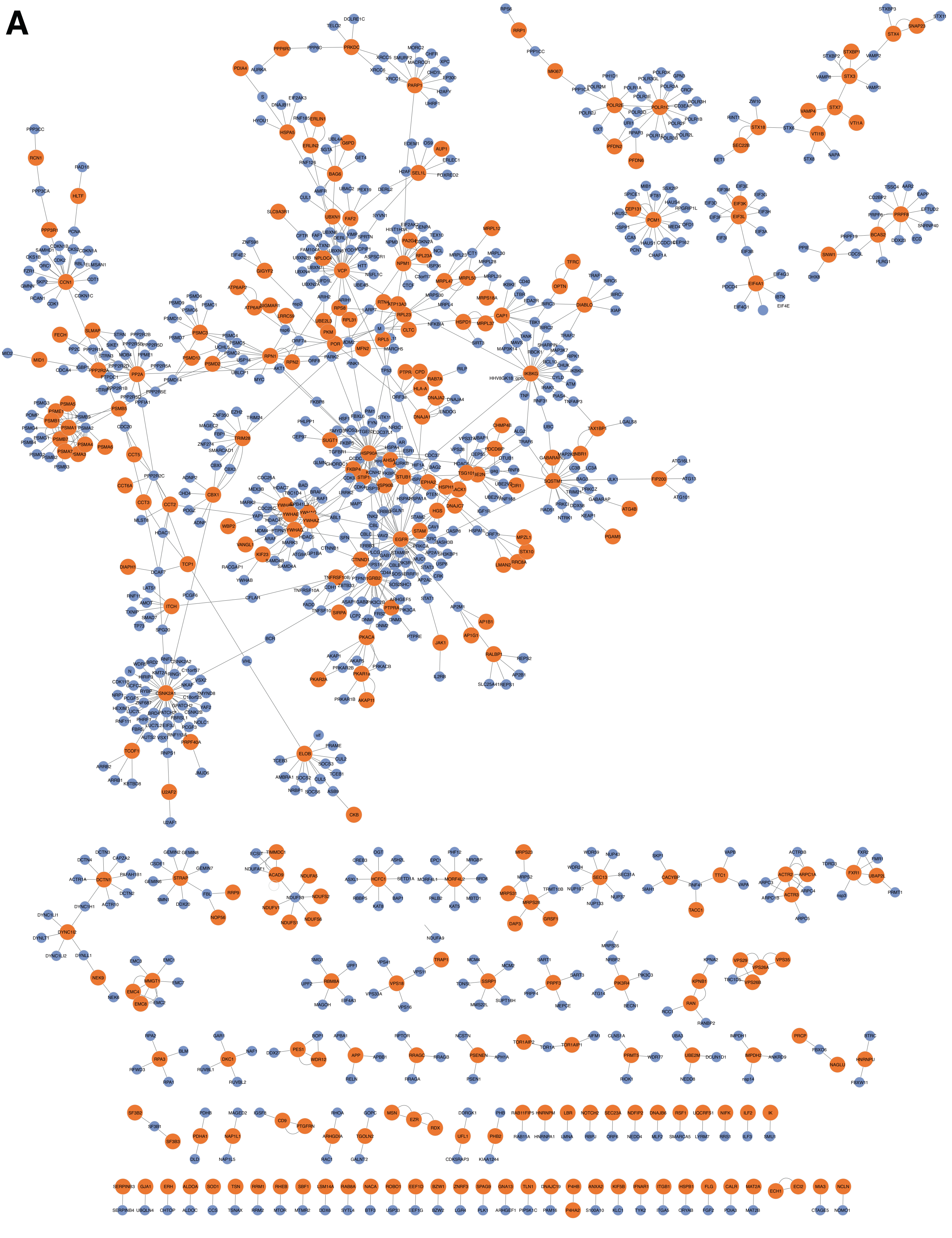
A

◀ **Figure EV2. Network of autophagic substrates.**

(**A**) Network representation of autophagy-dependent substrates (orange). Blue nodes show cited protein–protein interactions to provide context for which multi-protein complex substrates belong to.

 © The Author(s)

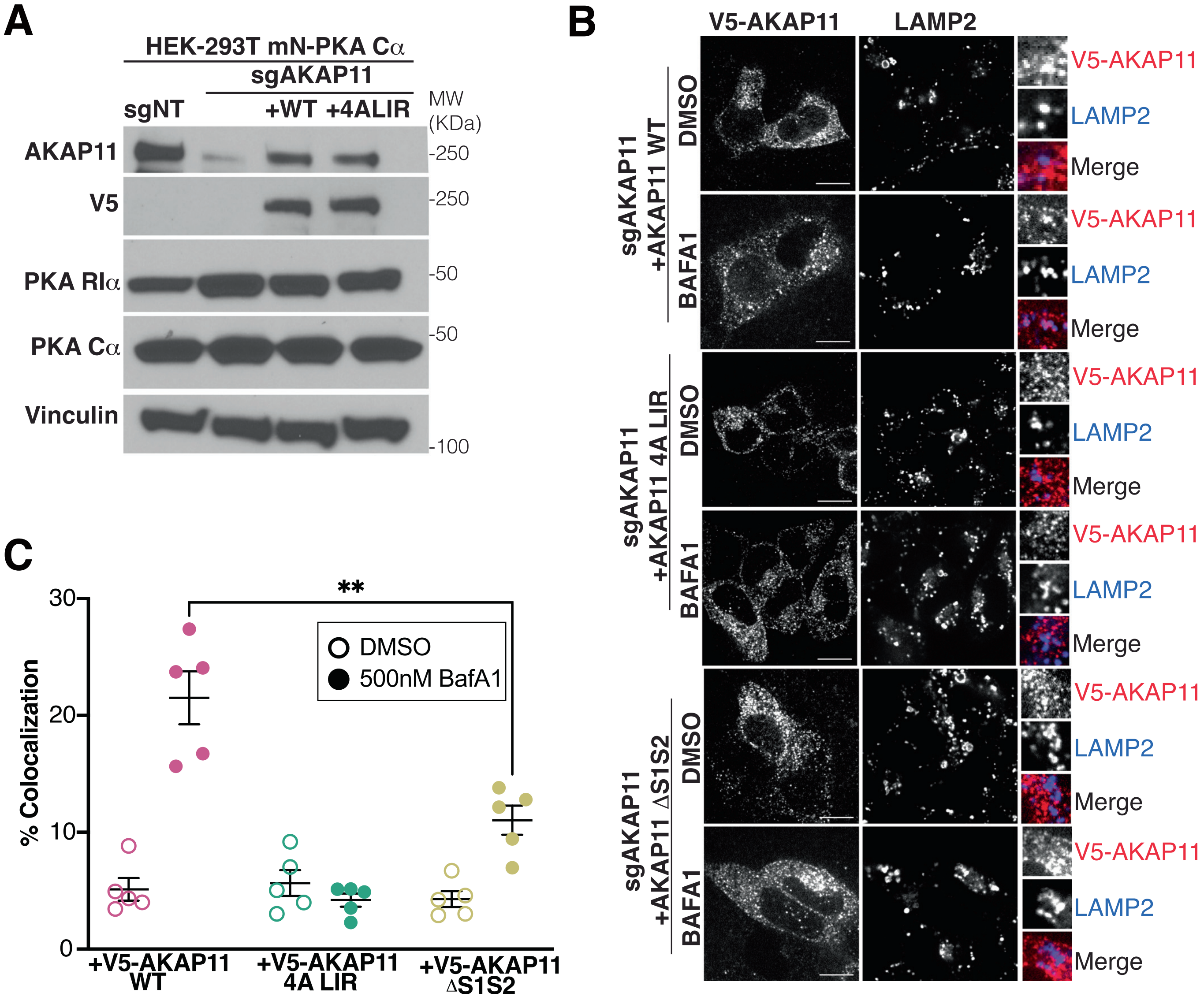

Figure EV3. PKA RIα binding defective AKAP11 has decreased lysosomal localization.

(**A**) Immunoblot validation of AKAP11 knockout using whole cell lysate from HEK293T sgNT or sgAKAP11 and sgAKAP11 with stably expressing the indicated V5-AKAP11 construct. (**B**) Immunofluorescence from HEK293T sgAKAP11 cells stably expressing V5-AKAP11 WT, V5-AKAP11 4ALIR (WSNL > AAAA), or V5-AKAP11 ΔS1S2 (deleted AA 615–628 & 1650–1663) were treated with 500 nM BafA1 or DMSO for 5 h before fixing and immunostaining for V5 and LAMP2. 10 μm scale bar. (**C**) Quantification of V5 and LAMP2 co-localization from 5 non-overlapping fields, with at least 3 cells per field; error bars presented as mean ± SEM, $**P$(adj.)= 0.0037, unpaired t test.

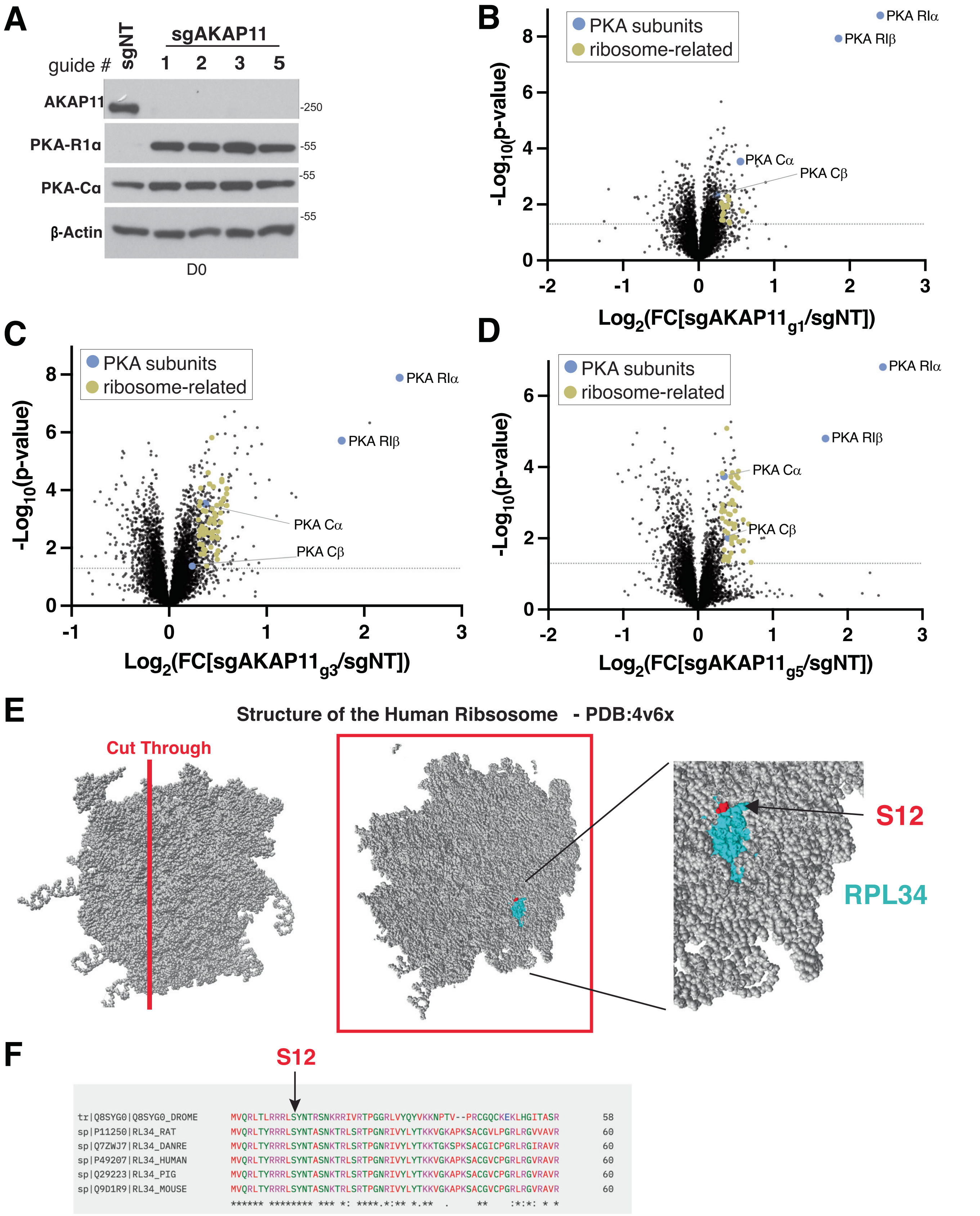
A
guide #
sgNT
sgAKAP11
1 2 3 5
AKAP11
PKA-R1α
PKA-Cα
β-Actin
-250
-55
-55
-55
D0
B
PKA subunits
ribosome-related
PKA RIα
PKA RIβ
PKA Cα
PKA Cβ
-Log10(p-value)
Log2(FC[sgAKAP11g1/sgNT])
C
PKA subunits
ribosome-related
PKA RIα
PKA RIβ
PKA Cα
PKA Cβ
-Log10(p-value)
Log2(FC[sgAKAP11g3/sgNT])
D
PKA subunits
ribosome-related
PKA RIα
PKA RIβ
PKA Cα
PKA Cβ
-Log10(p-value)
Log2(FC[sgAKAP11g5/sgNT])
E
Structure of the Human Ribsosome - PDB:4v6x
Cut Through
S12
RPL34
F
S12
tr|Q8SYG0|Q8SYG0_DROME MVQRLTLRRRLSYNTRSNKRRIVRTPGGRLVYQYVKKNPTV--PRCGQCKEKLHGITASR 58
sp|P11250|RL34_RAT MVQRLTYRRRLSYNTASNKTRLSRTPGNRIVYLYTKKVGKAPKSACGVLPGRLRGVVAVR 60
sp|Q7ZWJ7|RL34_DANRE MVQRLTYRRRLSYNTASNKTRLSRTPGNRIVYLYTKKTGKSPKSACGICPGRLRGIRAVR 60
sp|P49207|RL34_HUMAN MVQRLTYRRRLSYNTASNKTRLSRTPGNRIVYLYTKKVGKAPKSACGVCPGRLRGVRAVR 60
sp|Q29223|RL34_PIG MVQRLTYRRRLSYNTASNKTRLSRTPGNRIVYLYTKKVGKAPKSACGVCPGRLRGVRAVR 60
sp|Q9D1R9|RL34_MOUSE MVQRLTYRRRLSYNTASNKTRLSRTPGNRIVYLYTKKVGKAPKSACGVCPGRLRGVRAVR 60
****** ******** *** *: ****.*:** *.** . ** :*:*: * *

 © The Author(s)

Figure EV4. PKA holocomplex and ribosome related proteins are enriched in AKAP11-null i3 neurons.

(**A**) Immunoblot of whole cell lysate of DIV0 i3 neurons validating degree of knockdown of four sgAKAP11 guides compared to sgNT. (**B–D**) Proteomic analysis of DIV7 i3 neurons in 3 different guides targeting AKAP11. $n = 3$ independent biological replicates for all conditions. *p*-values calculated using two-tailed unpaired *t* test. Blue nodes indicate PKA subunits. Gold nodes indicate ribosome-associated proteins (ribosome, cytoplasmic and ribosome biogenesis). (**E**) The cryoEM structure of the human ribosome with RPL34 (cyan) deeply buried in the complex and Ser12 annotated in red. (**F**) Sequence alignment of RPL34 across species showing conservations of Ser12.

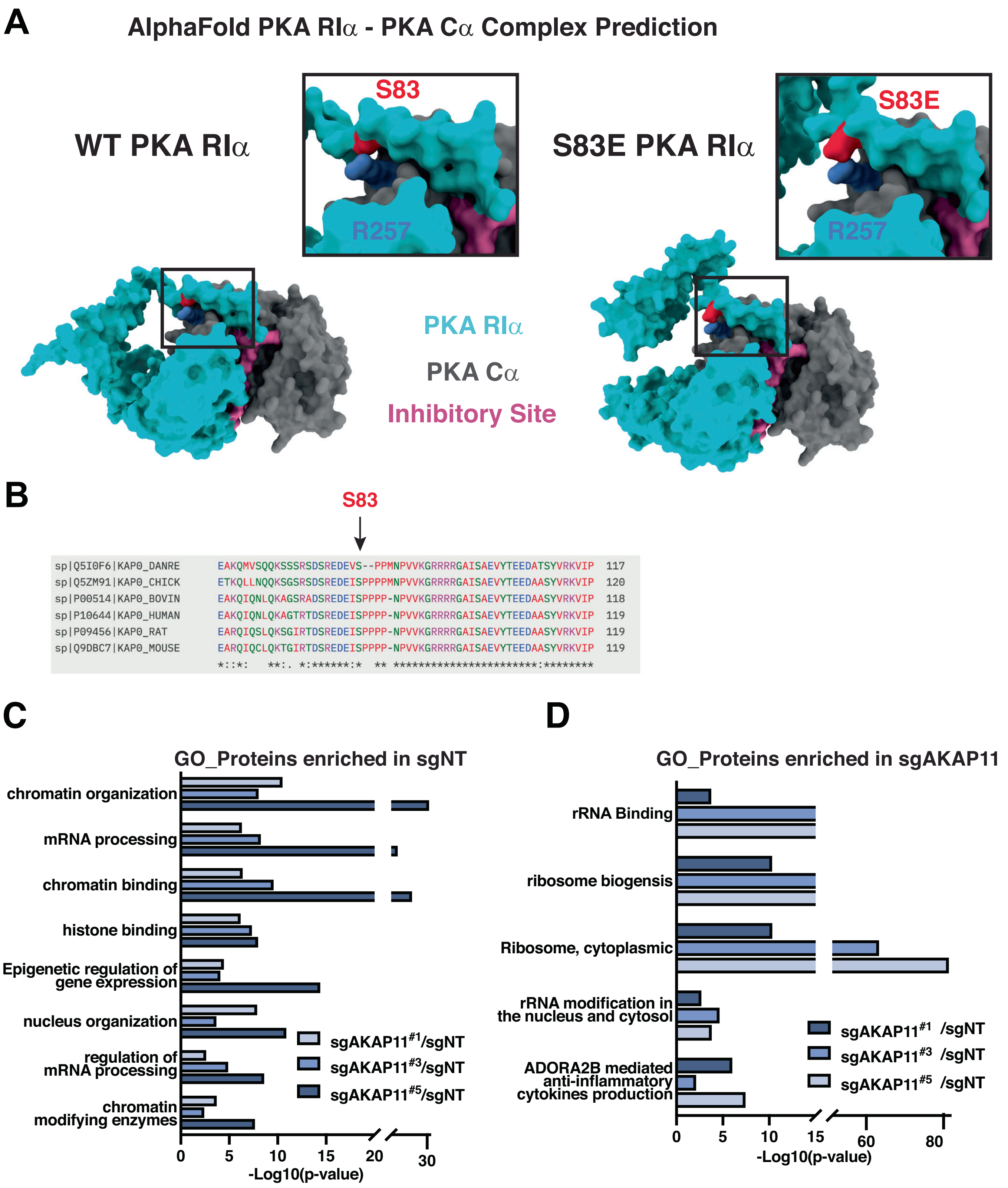
A
AlphaFold PKA RIα - PKA Cα Complex Prediction
WT PKA RIα
S83
R257
S83E PKA RIα
S83E
R257
PKA RIα
PKA Cα
Inhibitory Site
B
S83
sp|Q5I0F6|KAP0_DANRE EAKQMVSQQKSSSRSDSREDEVS--PPMNPVVKGRRRRGAISAEVYTEEDATSYVRKVIP 117
sp|Q5ZM91|KAP0_CHICK ETKQLLNQQKSGSRSDSREDEISPPPPMNPVVKGRRRRGAISAEVYTEEDAASYVRKVIP 120
sp|P00514|KAP0_BOVIN EAKQIQNLQKAGSRADSREDEISPPPP-NPVVKGRRRRGAISAEVYTEEDAASYVRKVIP 118
sp|P10644|KAP0_HUMAN EAKQIQNLQKAGTRTDSREDEISPPPP-NPVVKGRRRRGAISAEVYTEEDAASYVRKVIP 119
sp|P09456|KAP0_RAT EARQIQSLQKSGIRTDSREDEISPPPP-NPVVKGRRRRGAISAEVYTEEDAASYVRKVIP 119
sp|Q9DBC7|KAP0_MOUSE EARQIQCLQKTGIRTDSREDEISPPPP-NPVVKGRRRRGAISAEVYTEEDAASYVRKVIP 119
::: **:. *:******:* ** **********************:********
C
GO_Proteins enriched in sgNT
chromatin organization
mRNA processing
chromatin binding
histone binding
Epigenetic regulation of gene expression
nucleus organization
regulation of mRNA processing
chromatin modifying enzymes
sgAKAP11#1/sgNT
sgAKAP11#3/sgNT
sgAKAP11#5/sgNT
0 5 10 15 20 30
-Log10(p-value)
D
GO_Proteins enriched in sgAKAP11
rRNA Binding
ribosome biogensis
Ribosome, cytoplasmic
rRNA modification in the nucleus and cytosol
ADORA2B mediated anti-inflammatory cytokines production
sgAKAP11#1 /sgNT
sgAKAP11#3 /sgNT
sgAKAP11#5 /sgNT
0 5 10 15 60 80
-Log10(p-value)

© The Author(s)

Figure EV5. Structural and conservation analysis of PKA RIα Ser83.

(**A**) Alphafold prediction of the full length PKA holoenzyme complex, both WT and containing S83E mutant RIα. PKA RIα contains an inhibitory site (pink) that inserts in the active site of PKA Cα (gray). An unstructured loop nearby contains Ser83 (red). The S83E mutation, which mimics phosphorylation, is predicted to form a salt bridge with Arg257 of Cα and induce a significant conformational rearrangement in RIα. (**B**) Sequence alignment of PKA RIα across species showing conservation of Ser83. (**C**, **D**) Pathway enrichment analysis of DIV7 i3 neurons comparing (**C**) pathways enriched in sgNT (**D**) pathways enriched in sgAKAP11. Proteins included in the pathway enrichment analysis had a $-0.2 \geq Log_2FC \geq 0.2$ and *P* value < 0.05, two-tailed *t* test.

© The Author(s)

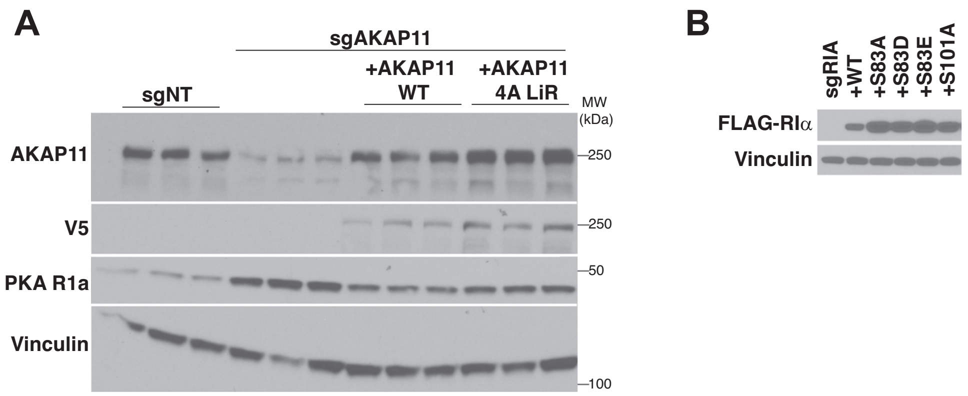

Figure EV6. Validation of knockdown and rescue of AKAP11 and RIa.

(**A**) Immunoblot of whole cell lysate from HEK293T sgNT and sgAKAP11 and sgAKAP11 cells stably expressing the indicated V5-AKAP11 construct in triplicate. Cells were grown in DMEM + 10% dFBS. These samples were used for phosphoproteomic experiments. (**B**) Western blots of sgRIα lysates used in Kemptide kinase assay showing vinculin as a loading control and transient expression of FLAG-RIα rescue constructs.

 © The Author(s)
